# Data on energy consumption in the production of layered double hydroxides

**DOI:** 10.1016/j.dib.2019.104408

**Published:** 2019-08-21

**Authors:** Luis Andrés Leal, Dayana Donneys-Victoria, Fiderman Machuca-Martínez

**Affiliations:** GAOX, Escuela de Ingeniería Química, Universidad del Valle, Santiago de Cali, Colombia

**Keywords:** Clays, Hydrotalcites, Electrocoagulation, Energy consumption, Electrochemical synthesis

## Abstract

Electrocoagulation consists of the in-situ generation of the coagulant by the electro dissolution of sacrificial electrodes (Mg and Al). This technique, besides being normally used for water treatment, can be used to synthesize Layered Double Hydroxides (LDH) or Hydrotalcites (HT) such as green rust, MgAlCl/LDH, and other oxides as Magnetite. The HT has a high tendency for water in the interlayer to be replaced by anions, these exchange characteristics generate a high interest in the fields of drug administration, photodegradation, catalyst supports, supercapacitors, and water oxidation. There are several routes of synthesis for these compounds such as co-precipitation, hydrolysis of urea, hydrothermal treatment and a novel route by electrocoagulation (EC).

This work discloses the data of the energy consumption at laboratory-scale production in the synthesis of hydrotalcite (HT) or Layered Double Hydroxides (LDH) by electrocoagulation, the values obtained through these experiments are intended to provide support due to the lack of information on the energy consumption of this novel production method. Aluminum and AZ31 electrodes were used as a cations source during two- and four-hours operation, at 50 °C with 5 mA cm^−2^ of current density, and 5 minutes of polarity change for Aluminum and 8 minutes for AZ31 (Magnesium alloy).

**Table:** Specifications table

Subject area	*Chemical Engineering.*
More specific subject area	*Nanomaterials*
Type of data	*Table and Figure*
How data was acquired	*Date were obtained by* power source BK Precision DC Regulated, FTIR and XRD.
Data format	*Raw, analyzed and descriptive data.*
Experimental factors	*The experiments were performed in an electrochemical cell with 6 electrodes (three anodes and three cathodes), the voltage was measured directly from the power source. The operational energy consumption was calculated based on the experimental data.*
Experimental features	*Data energy consumption and economic analysis of Mg–Al layered double hydroxides production in bench- scale testing*
Data source location	*Escuela de Ingeniería Química, Universidad del Valle, Cali, Colombia*
Data accessibility	*Data are within article*
Related research article	M. Molano-Mendoza, D. Donneys-Victoria, N. Marriaga-Cabrales, M.A. Mueses, G. Li Puma, F. Machuca-Martínez, Synthesis of Mg–Al layered double hydroxides by electrocoagulation, MethodsX. 5 (2018) 915–923. https://doi.org/10.1016/j.mex.2018.07.019[1]

**Table:** 

**Value of the data** • The economic feasibility of Mg–Al Production by electrocoagulation is shown. • The economic viability of Mg–Al layered double hydroxide production to continue pilot scale or higher is estimated. • The data about the energy consumption of layered double hydroxides by electrocoagulation allowed to validate the reproducibility of the technique. • The effect of the tension and the time in the operational cost of Mg–Al layered double hydroxides production is revealed.

## 1 Data

**Fig. 1:**
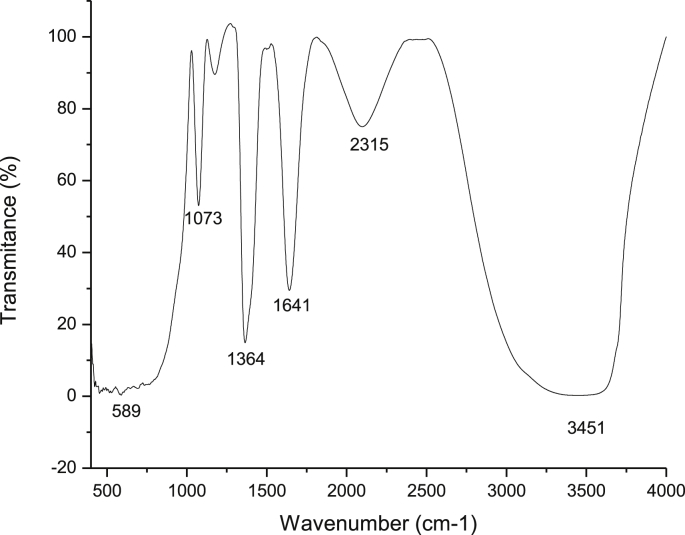
FTIR of a sample of the synthesized products.

**Fig. 2:**
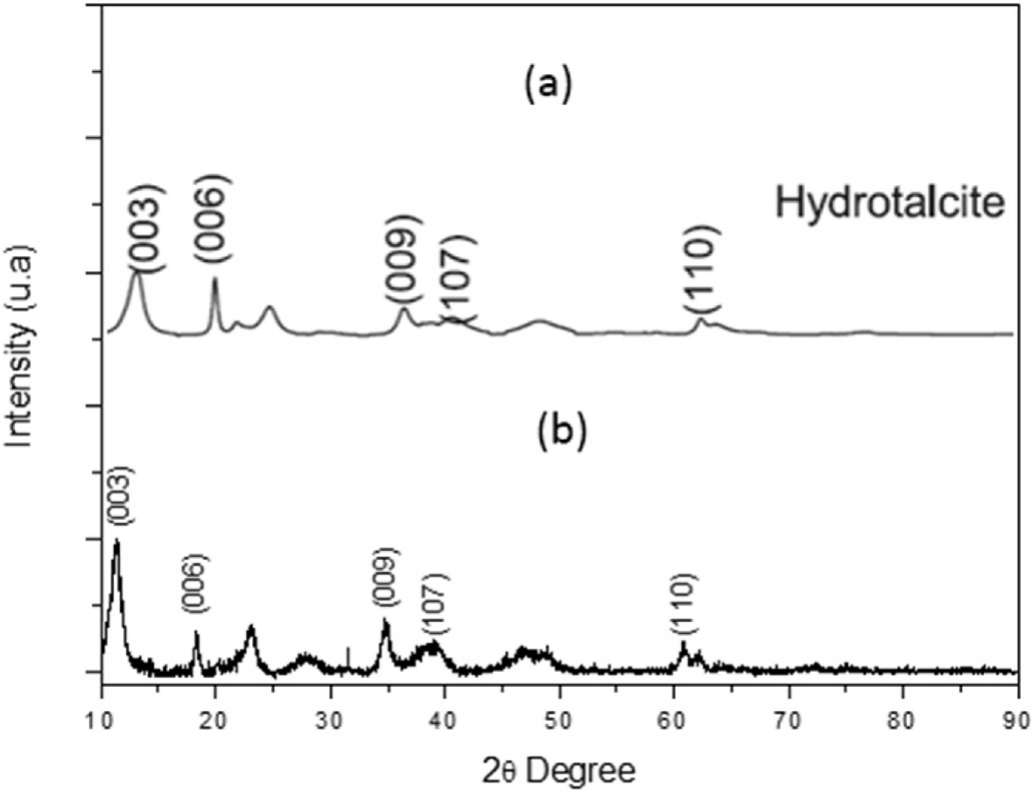
XRD spectra of the synthesized products. (a) Pattern LDH [1]. (b) Sample LDH.

The electrocoagulation (EC) process is generally used as wastewater treatment, this technique produces sludge and this use is related with the waste solid disposal. A new focus in the production of new materials by EC is emerging [2], [3], [4]. The LDH materials was prepared by Electrocoagulation according to methodology reported by Molano et al. [1], Fig. 1, Fig. 2 shows the typical FTIR and XRD spectra of synthesized materials. These raw data can be found in the attached supplementary data.

Table 1, Table 4 below show the values of the tension and power obtained during the synthesis of Mg–Al layered double hydroxides by using electrocoagulation with polarity change. Table 1, Table 2 contains data of nine experiments effectuated for 2 hours of production, while Table 3, Table 4 includes ten experiments made for 4 hours of production in equal operational conditions. The experimental conditions were: 50 °C, 5000 mg L^−1^ of NaCl as electrolyte and 1.84 A.

**Table 1:** Energy consumption for LDH production, 2 hours process time. Experiment 1 to 5.

Time Hours	Experiment 1		Experiment 2		Experiment 3		Experiment 4		Experiment 5	
	Tension V	Power kW (10¯³)	Tension V	Power kW (10¯³)	Tension V	Power kW (10¯³)	Tension V	Power kW (10¯³)	Tension V	Power kW (10¯³)
0,00	1,30	2,39	1,30	2,39	1,00	1,84	0,90	1,66	1,40	2,58
0,08	2,00	3,68	1,90	3,50	1,70	3,13	1,80	3,31	1,80	3,31
0,22	1,00	1,84	0,90	1,66	1,30	2,39	0,90	1,66	0,90	1,66
0,30	1,80	3,31	1,80	3,31	1,70	3,13	1,60	2,94	1,80	3,31
0,43	0,90	1,66	0,90	1,66	0,90	1,66	0,70	1,29	1,00	1,84
0,52	1,80	3,31	1,80	3,31	1,70	3,13	1,60	2,94	1,80	3,31
0,65	1,00	1,84	0,80	1,47	1,60	2,94	0,60	1,10	1,10	2,02
0,73	1,80	3,31	1,80	3,31	1,60	2,94	1,60	2,94	2,00	3,68
0,87	1,00	1,84	1,00	1,84	1,70	3,13	0,70	1,29	1,20	2,21
0,95	1,80	3,31	1,80	3,31	1,70	3,13	1,70	3,13	2,00	3,68
1,08	1,10	2,02	1,10	2,02	0,80	1,47	0,70	1,29	1,20	2,21
1,17	1,80	3,31	1,90	3,50	2,00	3,68	1,70	3,13	2,10	3,86
1,30	1,10	2,02	0,90	1,66	1,30	2,39	0,80	1,47	1,20	2,21
1,38	1,80	3,31	1,80	3,31	1,70	3,13	1,70	3,13	2,10	3,86
1,52	0,90	1,66	1,00	1,84	0,80	1,47	0,70	1,29	1,20	2,21
1,60	1,70	3,13	1,90	3,50	1,70	3,13	1,70	3,13	2,10	3,86
1,73	0,80	1,47	1,10	2,02	1,00	1,84	0,80	1,47	1,20	2,21
1,82	1,80	3,31	1,80	3,31	1,90	3,50	1,70	3,13	2,10	3,86
1,95	0,90	1,66	1,00	1,84	1,00	1,84	0,70	1,29	1,20	2,21
2,03	1,80	3,31	1,80	3,31	1,90	3,50	1,70	3,13	2,10	3,86

**Table 2:** Energy consumption for LDH production, 2 hours process time. Experiment 5 to 9.

Time Hours	Experiment 6		Experiment 7		Experiment 8		Experiment 9	
	Tension V	Power kW (10¯³)	Tension V	Power kW (10¯³)	Tension V	Power kW (10¯³)	Tension V	Power kW (10¯³)
0,00	1,00	1,84	1,40	2,58	1,43	2,63	1,29	2,37
0,08	2,00	3,68	1,90	3,50	1,97	3,62	1,99	3,66
0,22	0,90	1,66	0,90	1,66	0,84	1,55	2,25	4,14
0,30	1,80	3,31	1,80	3,31	1,68	3,09	2,03	3,74
0,43	0,90	1,66	1,00	1,84	1,19	2,19	1,18	2,17
0,52	1,60	2,94	1,80	3,31	1,68	3,09	1,95	3,59
0,65	0,90	1,66	0,80	1,47	0,91	1,67	1,18	2,17
0,73	1,90	3,50	1,80	3,31	1,71	3,15	1,98	3,64
0,87	1,20	2,21	0,80	1,47	0,80	1,47	1,79	3,29
0,95	2,10	3,86	1,80	3,31	1,72	3,16	2,03	3,74
1,08	1,10	2,02	1,10	2,02	0,99	1,82	1,03	1,90
1,17	2,00	3,68	1,81	3,33	1,72	3,16	2,00	3,68
1,30	1,10	2,02	1,02	1,88	0,86	1,58	1,46	2,69
1,38	2,10	3,86	1,86	3,42	1,69	3,11	2,04	3,75
1,52	1,20	2,21	1,05	1,93	0,79	1,45	0,98	1,80
1,60	2,10	3,86	1,84	3,39	1,73	3,18	2,11	3,88
1,73	1,20	2,21	1,08	1,99	0,98	1,80	0,96	1,77
1,82	–	–	1,81	3,33	1,58	2,91	2,05	3,77
1,95	–	–	0,96	1,77	0,80	1,47	1,07	1,97
2,03	–	–	1,81	3,33	1,58	2,91	–	–

**Table 3:** Energy consumption for LDH production, 4 hours process time. Experiment 10 to 14.

Time Hours	Experiment 10		Experiment 11		Experiment 12		Experiment 13		Experiment 14	
	Tension V	Power kW (10¯³)	Tension V	Power kW (10¯³)	Tension V	Power kW (10¯³)	Tension V	Power kW (10¯³)	Tension V	Power kW (10¯³)
0,00	1,18	2,17	1,16	2,13	1,26	2,32	1,81	3,33	1,68	3,09
0,08	1,86	3,42	2,00	3,68	2,02	3,72	2,60	4,78	2,16	3,97
0,22	1,53	2,82	1,09	2,01	1,53	2,82	1,11	2,04	1,25	2,30
0,30	1,81	3,33	1,96	3,61	2,00	3,68	2,05	3,77	1,97	3,62
0,43	1,18	2,17	1,05	1,93	1,20	2,21	1,14	2,10	1,28	2,36
0,52	1,74	3,20	1,92	3,53	2,07	3,81	2,17	3,99	2,07	3,81
0,65	1,90	3,50	1,02	1,88	1,01	1,86	1,04	1,91	1,21	2,23
0,73	1,80	3,31	1,95	3,59	1,95	3,59	2,12	3,90	2,04	3,75
0,87	1,97	3,62	1,10	2,02	1,27	2,34	1,03	1,90	1,47	2,70
0,95	1,80	3,31	1,97	3,62	2,04	3,75	2,02	3,72	2,10	3,86
1,08	0,92	1,69	1,35	2,48	1,13	2,08	1,12	2,06	1,35	2,48
1,17	1,61	2,96	2,00	3,68	1,99	3,66	2,19	4,03	2,31	4,25
1,30	0,90	1,66	1,05	1,93	1,04	1,91	1,10	2,02	1,64	3,02
1,38	1,75	3,22	1,99	3,66	2,15	3,96	2,00	3,68	2,08	3,83
1,52	0,97	1,78	1,09	2,01	1,11	2,04	1,11	2,04	1,66	3,05
1,60	1,74	3,20	2,04	3,75	2,04	3,75	1,93	3,55	2,28	4,20
1,73	1,04	1,91	1,42	2,61	1,04	1,91	1,14	2,10	1,52	2,80
1,82	1,75	3,22	1,97	3,62	2,09	3,85	1,83	3,37	2,19	4,03
1,95	0,94	1,73	1,22	2,24	1,20	2,21	1,72	3,16	1,42	2,61
2,03	1,77	3,26	1,99	3,66	1,97	3,62	1,48	2,72	2,33	4,29
2,17	0,93	1,71	1,22	2,24	1,11	2,04	2,21	4,07	1,51	2,78
2,25	1,73	3,18	1,99	3,66	2,03	3,74	1,11	2,04	2,16	3,97
2,38	0,90	1,66	1,16	2,13	1,06	1,95	2,25	4,14	1,54	2,83
2,47	1,78	3,28	2,00	3,68	2,05	3,77	1,45	2,67	2,23	4,10
2,60	1,37	2,52	1,15	2,12	1,10	2,02	1,40	2,58	1,49	2,74
2,68	1,93	3,55	2,00	3,68	2,09	3,85	2,10	3,86	2,23	4,10
2,82	0,98	1,80	1,38	2,54	1,15	2,12	1,39	2,56	1,24	2,28
2,90	1,82	3,35	2,07	3,81	2,16	3,97	2,30	4,23	2,35	4,32
3,03	0,86	1,58	1,34	2,47	1,09	2,01	2,23	4,10	1,33	2,45
3,12	1,85	3,40	2,02	3,72	1,98	3,64	2,47	4,54	2,30	4,23
3,25	0,97	1,78	1,32	2,43	1,95	3,59	1,25	2,30	1,56	2,87
3,33	2,03	3,74	1,94	3,57	1,99	3,66	1,99	3,66	2,36	4,34
3,47	1,10	2,02	1,19	2,19	1,09	2,01	1,41	2,59	1,52	2,80
3,55	1,18	2,17	2,00	3,68	1,96	3,61	2,24	4,12	2,30	4,23
3,68	0,95	1,75	1,15	2,12	1,12	2,06	1,49	2,74	1,55	2,85
3,77	2,05	3,77	2,00	3,68	2,02	3,72	2,27	4,18	–	–
3,90	0,96	1,77	1,32	2,43	1,13	2,08	1,52	2,80	–	–
3,98	1,95	3,59	1,94	3,57	2,05	3,77	2,30	4,23	–	–

**Table 4:** Energy consumption for LDH production, 4 hours process time. Experiment 15 to 19.

Time Hours	Experiment 15		Experiment 16		Experiment 17		Experiment 18		Experiment 19	
	Tension V	Power kW (10¯³)	Tension V	Power kW (10¯³)	Tension V	Power kW (10¯³)	Tension V	Power kW (10¯³)	Tension V	Power kW (10¯³)
0,00	1,68	3,09	1,37	2,52	0,98	1,80	0,98	1,80	0,88	1,62
0,08	2,16	3,97	2,01	3,70	1,74	3,20	1,88	3,46	1,64	3,02
0,22	1,25	2,30	2,07	3,81	0,82	1,51	1,34	2,47	0,75	1,38
0,30	1,97	3,62	1,91	3,51	1,76	3,24	1,88	3,46	1,73	3,18
0,43	1,28	2,36	0,99	1,82	0,83	1,53	0,88	1,62	0,86	1,58
0,52	2,07	3,81	1,93	3,55	1,76	3,24	1,81	3,33	1,70	3,13
0,65	1,21	2,23	1,34	2,47	0,80	1,47	1,09	2,01	0,90	1,66
0,73	2,04	3,75	1,98	3,64	1,66	3,05	1,80	3,31	1,70	3,13
0,87	1,47	2,70	1,03	1,90	0,70	1,29	1,34	2,47	0,80	1,47
0,95	2,10	3,86	1,88	3,46	1,68	3,09	1,81	3,33	1,65	3,04
1,08	1,35	2,48	1,12	2,06	1,94	3,57	1,17	2,15	1,18	2,17
1,17	2,31	4,25	1,99	3,66	1,78	3,28	1,18	2,17	1,72	3,16
1,30	1,64	3,02	1,18	2,17	1,07	1,97	2,23	4,10	1,10	2,02
1,38	2,08	3,83	1,93	3,55	1,75	3,22	1,83	3,37	1,62	2,98
1,52	1,66	3,05	1,18	2,17	2,09	3,85	1,40	2,58	1,11	2,04
1,60	2,28	4,20	1,87	3,44	1,79	3,29	1,87	3,44	1,76	3,24
1,73	1,52	2,80	1,13	2,08	0,85	1,56	1,04	1,91	1,24	2,28
1,82	2,19	4,03	1,86	3,42	1,76	3,24	1,81	3,33	1,78	3,28
1,95	1,42	2,61	0,98	1,80	0,86	1,58	1,32	2,43	1,12	2,06
2,03	2,33	4,29	1,67	3,07	1,71	3,15	1,78	3,28	1,74	3,20
2,17	1,51	2,78	1,21	2,23	1,02	1,88	1,08	1,99	0,93	1,71
2,25	2,16	3,97	1,92	3,53	1,78	3,28	1,81	3,33	1,77	3,26
2,38	1,54	2,83	0,97	1,78	0,93	1,71	1,09	2,01	0,97	1,78
2,47	2,23	4,10	1,91	3,51	1,74	3,20	1,84	3,39	1,81	3,33
2,60	1,49	2,74	1,40	2,58	1,15	2,12	1,15	2,12	1,03	1,90
2,68	2,23	4,10	1,96	3,61	1,80	3,31	1,84	3,39	1,29	2,37
2,82	1,24	2,28	1,19	2,19	0,90	1,66	0,96	1,77	0,97	1,78
2,90	2,35	4,32	2,00	3,68	1,77	3,26	1,85	3,40	1,74	3,20
3,03	1,33	2,45	1,07	1,97	0,78	1,44	1,37	2,52	0,80	1,47
3,12	2,30	4,23	1,86	3,42	1,76	3,24	1,85	3,40	1,88	3,46
3,25	1,56	2,87	1,70	3,13	1,12	2,06	0,99	1,82	1,06	1,95
3,33	2,36	4,34	1,90	3,50	1,77	3,26	1,74	3,20	1,87	3,44
3,47	1,52	2,80	1,64	3,02	1,05	1,93	1,36	2,50	1,31	2,41
3,55	2,30	4,23	1,94	3,57	1,84	3,39	1,84	3,39	1,87	3,44
3,68	1,55	2,85	1,70	3,13	1,26	2,32	0,93	1,71	1,11	2,04
3,77	–	–	1,93	3,55	1,85	3,40	1,76	3,24	–	–
3,90	–	–	1,28	2,36	–	–	–	–	–	–
3,98	–	–	–	–	–	–	–	–	–	–

Table 5 shows the weight of LDH obtained during each experiment on the other hand, Table 6 presents the total operational energy consumption in bench-scale testing.

**Table 5:** Weight production of LDH.

Experiment	Weight (g)
1	8.29
2	8.50
3	7.75
4	7.78
5	8.04
6	8.09
7	8.48
8	8.82
9	8.00
**Total (2 h)**	**73.75**
10	14.97
11	16.66
12	15.50
13	15.95
14	13.50
15	13.60
16	16.76
17	13.42
18	13.32
19	13.60
**Total (4 h)**	**147.28**

**Table 6:** Total operational cost in bench-scale testing.

Conditions	Energy operational Kwh/Kg	
*AZ3*1-Al Electrodes at 50 °C, 1,84 A, 5000 mg L^−1^ NaCl	2 Hours	0.641
	4 Hours	0.741

## 2 Experimental design, materials, and methods

NaCl solution was prepared by dissolving 5000 mg of sodium chloride (Sigma Aldrich-reagent grade) in 1 L of purified water obtained by drinking water distillation, previously filtered (0.45 μm) and subjected to adsorption with activated carbon, in a Water Pro PS Labconco equipment. To measure the pH and electrical conductivity (mS cm^−1^ at 25 °C) a multiparameter sensor Thermo Scientific Orion Star A329 was used [5], [6].

### 2.1 Experimental assembly

A 2000 mL beaker was used as an electrolytic cell with 1400 mL of initial solution volume. AZ31 alloy (weight composition: 95.56% Magnesium, 3.0% Aluminum, 1.0% Zinc, 0.043% Manganese, 0.01% Silicon, Copper <0.01%, nickel <0.001% and 0.003% Iron) and Aluminum were used as electrodes. These plates, with an effective area of 168 cm^2^, respectively, were suspended and clamped together using polyethylene belts, guaranteeing an interelectrode distance of 0.5 cm. For heating and stirring, a Thermo Scientific brand plate model SP131635Q was used, with a 3 cm long and 0.5 cm in diameter magnetic stirrer, and a Brisco thermometer. The electrodes were connected to a direct current power source BK Precision DC Regulated Power Supply model 1665 with a maximum amperage of 5 A, in a monopolar arrangement connected to a polarity inverter followed by the power source [6].

Before each experimental test, the electrodes were manually polish with sandpaper gauge 150, 400 and 600. The cell tension, pH, temperature, and electrical conductivity were continuously monitored.

### 2.2 Characterization

The HT synthesized were characterized by X-ray diffraction (XRD) and Fourier Transform Infrared (FTIR), the FTIR spectra were recorded with a JASCO FT/IR-4100 brand equipment, in a range between 500 and 4000 cm^−1^, the X-ray diffraction pattern (XRD) was performed with an analytical diffractometer called X'pert PRO - PANalytical at conditions of 45 kV, 40 mA, monochromatic CuKα radiation at a λ = 0.1542 nm and in a 2θ range of 4°–90° [1].
