# Supplementary material for: Data on energy consumption in the production of layered double hydroxides
Source: Data Brief. 2019 Aug 21;26:104408. doi: 10.1016/j.dib.2019.104408 (PMC6743027; doi:10.1016/j.dib.2019.104408)
Supplement: Multimedia component 1 [file mmc1.docx]

*Data on energy consumption in the production of layered double hydroxides*

*Luis Andrés Leal^1^, Dayana Donneys-Victoria^1^ and Fiderman Machuca-Martínez^1^.*

*^1^Escuela de Ingeniería Química, Universidad del Valle, Santiago de Cali, Colombia*

Email: [luis.leal@correounivalle.edu.co](mailto:luis.leal@correounivalle.edu.co), [dayana.donneys@correounivalle.edu.co](mailto:dayana.donneys@correounivalle.edu.co), [fiderman.machuca@correounivalle](mailto:fiderman.machuca@correounivalle).co

**Supplementary data**

Table S1 and S2 show the raw data of the FTIR and DRX from Figure 1 and Figure 2.

Table S1. Data FTIR of LHD .

| Wavenumber cm^-1^ | Transmitance % |
| --- | --- |
| 349,1 | 9,2 |
| 350,0 | 9,2 |
| 351,0 | 11,0 |
| 351,9 | 13,8 |
| 352,9 | 16,7 |
| 353,9 | 19,4 |
| 354,8 | 21,2 |
| 355,8 | 21,6 |
| 356,8 | 21,6 |
| 357,7 | 21,6 |
| 358,7 | 21,6 |
| 359,7 | 21,6 |
| 360,6 | 21,6 |
| 361,6 | 22,1 |
| 362,6 | 24,8 |
| 363,5 | 29,3 |
| 364,5 | 34,6 |
| 365,4 | 38,3 |
| 366,4 | 37,8 |
| 367,4 | 36,5 |
| 368,3 | 38,0 |
| 369,3 | 38,7 |
| 370,3 | 38,8 |
| 371,2 | 38,8 |
| 372,2 | 38,9 |
| 373,2 | 39,5 |
| 374,1 | 40,4 |
| 375,1 | 39,4 |
| 376,1 | 37,1 |
| 377,0 | 34,1 |
| 378,0 | 31,5 |
| 378,9 | 29,7 |
| 379,9 | 29,3 |
| 380,9 | 30,0 |
| 381,8 | 31,2 |
| 382,8 | 32,9 |
| 383,8 | 34,6 |
| 384,7 | 36,0 |
| 385,7 | 36,2 |
| 386,7 | 33,6 |
| 387,6 | 29,0 |
| 388,6 | 23,8 |
| 389,6 | 18,9 |
| 390,5 | 15,4 |
| 391,5 | 13,5 |
| 392,4 | 13,0 |
| 393,4 | 13,4 |
| 394,4 | 14,4 |
| 395,3 | 15,2 |
| 396,3 | 15,8 |
| 397,3 | 15,8 |
| 398,2 | 15,4 |
| 399,2 | 15,0 |
| 400,2 | 14,7 |
| 401,1 | 14,7 |
| 402,1 | 14,7 |
| 403,0 | 14,7 |
| 404,0 | 14,6 |
| 405,0 | 13,9 |
| 405,9 | 13,1 |
| 406,9 | 12,0 |
| 407,9 | 10,8 |
| 408,8 | 9,7 |
| 409,8 | 9,1 |
| 410,8 | 9,0 |
| 411,7 | 9,0 |
| 412,7 | 9,0 |
| 413,7 | 9,0 |
| 414,6 | 8,7 |
| 415,6 | 8,1 |
| 416,5 | 7,1 |
| 417,5 | 6,1 |
| 418,5 | 5,1 |
| 419,4 | 4,2 |
| 420,4 | 3,5 |
| 421,4 | 3,0 |
| 422,3 | 2,7 |
| 423,3 | 2,7 |
| 424,3 | 2,9 |
| 425,2 | 3,3 |
| 426,2 | 3,7 |
| 427,2 | 4,0 |
| 428,1 | 4,2 |
| 429,1 | 4,2 |
| 430,0 | 4,0 |
| 431,0 | 3,4 |
| 432,0 | 3,0 |
| 432,9 | 2,7 |
| 433,9 | 2,7 |
| 434,9 | 2,7 |
| 435,8 | 2,7 |
| 436,8 | 2,7 |
| 437,8 | 2,7 |
| 438,7 | 2,7 |
| 439,7 | 2,7 |
| 440,7 | 2,7 |
| 441,6 | 2,7 |
| 442,6 | 2,7 |
| 443,5 | 2,8 |
| 444,5 | 2,9 |
| 445,5 | 2,9 |
| 446,4 | 2,8 |
| 447,4 | 2,5 |
| 448,4 | 2,1 |
| 449,3 | 1,7 |
| 450,3 | 1,3 |
| 451,3 | 1,1 |
| 452,2 | 0,9 |
| 453,2 | 0,8 |
| 454,2 | 0,9 |
| 455,1 | 1,0 |
| 456,1 | 1,1 |
| 457,0 | 1,1 |
| 458,0 | 1,1 |
| 459,0 | 1,2 |
| 459,9 | 1,3 |
| 460,9 | 1,4 |
| 461,9 | 1,5 |
| 462,8 | 1,6 |
| 463,8 | 1,7 |
| 464,8 | 1,7 |
| 465,7 | 1,7 |
| 466,7 | 1,7 |
| 467,7 | 1,6 |
| 468,6 | 1,5 |
| 469,6 | 1,4 |
| 470,5 | 1,4 |
| 471,5 | 1,5 |
| 472,5 | 1,7 |
| 473,4 | 1,9 |
| 474,4 | 2,1 |
| 475,4 | 2,3 |
| 476,3 | 2,4 |
| 477,3 | 2,3 |
| 478,3 | 2,2 |
| 479,2 | 2,0 |
| 480,2 | 1,9 |
| 481,2 | 1,8 |
| 482,1 | 1,8 |
| 483,1 | 1,8 |
| 484,0 | 1,8 |
| 485,0 | 1,8 |
| 486,0 | 1,8 |
| 486,9 | 1,9 |
| 487,9 | 2,0 |
| 488,9 | 2,2 |
| 489,8 | 2,3 |
| 490,8 | 2,4 |
| 491,8 | 2,4 |
| 492,7 | 2,4 |
| 493,7 | 2,4 |
| 494,7 | 2,3 |
| 495,6 | 2,2 |
| 496,6 | 1,9 |
| 497,5 | 1,7 |
| 498,5 | 1,6 |
| 499,5 | 1,6 |
| 500,4 | 1,6 |
| 501,4 | 1,8 |
| 502,4 | 1,9 |
| 503,3 | 2,0 |
| 504,3 | 2,0 |
| 505,3 | 2,0 |
| 506,2 | 1,9 |
| 507,2 | 1,9 |
| 508,2 | 1,9 |
| 509,1 | 1,8 |
| 510,1 | 1,7 |
| 511,0 | 1,6 |
| 512,0 | 1,4 |
| 513,0 | 1,2 |
| 513,9 | 1,1 |
| 514,9 | 1,0 |
| 515,9 | 1,0 |
| 516,8 | 1,0 |
| 517,8 | 1,1 |
| 518,8 | 1,2 |
| 519,7 | 1,3 |
| 520,7 | 1,4 |
| 521,7 | 1,4 |
| 522,6 | 1,5 |
| 523,6 | 1,4 |
| 524,5 | 1,3 |
| 525,5 | 1,2 |
| 526,5 | 1,1 |
| 527,4 | 1,0 |
| 528,4 | 1,0 |
| 529,4 | 1,0 |
| 530,3 | 1,1 |
| 531,3 | 1,1 |
| 532,3 | 1,1 |
| 533,2 | 1,2 |
| 534,2 | 1,3 |
| 535,1 | 1,4 |
| 536,1 | 1,5 |
| 537,1 | 1,6 |
| 538,0 | 1,7 |
| 539,0 | 1,7 |
| 540,0 | 1,8 |
| 540,9 | 1,9 |
| 541,9 | 1,9 |
| 542,9 | 2,0 |
| 543,8 | 2,1 |
| 544,8 | 2,1 |
| 545,8 | 2,1 |
| 546,7 | 2,1 |
| 547,7 | 2,2 |
| 548,6 | 2,2 |
| 549,6 | 2,3 |
| 550,6 | 2,4 |
| 551,5 | 2,5 |
| 552,5 | 2,6 |
| 553,5 | 2,6 |
| 554,4 | 2,6 |
| 555,4 | 2,6 |
| 556,4 | 2,5 |
| 557,3 | 2,4 |
| 558,3 | 2,3 |
| 559,3 | 2,2 |
| 560,2 | 2,1 |
| 561,2 | 2,1 |
| 562,1 | 2,1 |
| 563,1 | 2,1 |
| 564,1 | 2,0 |
| 565,0 | 2,0 |
| 566,0 | 1,9 |
| 567,0 | 1,8 |
| 567,9 | 1,7 |
| 568,9 | 1,5 |
| 569,9 | 1,4 |
| 570,8 | 1,3 |
| 571,8 | 1,2 |
| 572,8 | 1,1 |
| 573,7 | 1,0 |
| 574,7 | 0,9 |
| 575,6 | 0,8 |
| 576,6 | 0,7 |
| 577,6 | 0,6 |
| 578,5 | 0,6 |
| 579,5 | 0,6 |
| 580,5 | 0,6 |
| 581,4 | 0,6 |
| 582,4 | 0,7 |
| 583,4 | 0,6 |
| 584,3 | 0,6 |
| 585,3 | 0,5 |
| 586,3 | 0,4 |
| 587,2 | 0,4 |
| 588,2 | 0,3 |
| 589,1 | 0,3 |
| 590,1 | 0,3 |
| 591,1 | 0,4 |
| 592,0 | 0,5 |
| 593,0 | 0,6 |
| 594,0 | 0,7 |
| 594,9 | 0,8 |
| 595,9 | 0,9 |
| 596,9 | 1,0 |
| 597,8 | 1,1 |
| 598,8 | 1,1 |
| 599,8 | 1,2 |
| 600,7 | 1,2 |
| 601,7 | 1,2 |
| 602,6 | 1,2 |
| 603,6 | 1,2 |
| 604,6 | 1,1 |
| 605,5 | 1,1 |
| 606,5 | 1,1 |
| 607,5 | 1,2 |
| 608,4 | 1,3 |
| 609,4 | 1,4 |
| 610,4 | 1,5 |
| 611,3 | 1,6 |
| 612,3 | 1,7 |
| 613,3 | 1,7 |
| 614,2 | 1,8 |
| 615,2 | 1,8 |
| 616,1 | 1,8 |
| 617,1 | 1,8 |
| 618,1 | 1,8 |
| 619,0 | 1,8 |
| 620,0 | 1,8 |
| 621,0 | 1,9 |
| 621,9 | 1,9 |
| 622,9 | 2,0 |
| 623,9 | 2,0 |
| 624,8 | 2,1 |
| 625,8 | 2,2 |
| 626,8 | 2,2 |
| 627,7 | 2,3 |
| 628,7 | 2,4 |
| 629,6 | 2,4 |
| 630,6 | 2,4 |
| 631,6 | 2,4 |
| 632,5 | 2,4 |
| 633,5 | 2,4 |
| 634,5 | 2,4 |
| 635,4 | 2,4 |
| 636,4 | 2,4 |
| 637,4 | 2,4 |
| 638,3 | 2,4 |
| 639,3 | 2,3 |
| 640,3 | 2,3 |
| 641,2 | 2,3 |
| 642,2 | 2,3 |
| 643,1 | 2,2 |
| 644,1 | 2,2 |
| 645,1 | 2,2 |
| 646,0 | 2,2 |
| 647,0 | 2,2 |
| 648,0 | 2,2 |
| 648,9 | 2,2 |
| 649,9 | 2,2 |
| 650,9 | 2,3 |
| 651,8 | 2,3 |
| 652,8 | 2,4 |
| 653,8 | 2,4 |
| 654,7 | 2,5 |
| 655,7 | 2,5 |
| 656,6 | 2,5 |
| 657,6 | 2,5 |
| 658,6 | 2,6 |
| 659,5 | 2,6 |
| 660,5 | 2,6 |
| 661,5 | 2,7 |
| 662,4 | 2,7 |
| 663,4 | 2,8 |
| 664,4 | 2,8 |
| 665,3 | 2,8 |
| 666,3 | 2,8 |
| 667,2 | 2,8 |
| 668,2 | 2,8 |
| 669,2 | 2,8 |
| 670,1 | 2,7 |
| 671,1 | 2,7 |
| 672,1 | 2,6 |
| 673,0 | 2,6 |
| 674,0 | 2,6 |
| 675,0 | 2,5 |
| 675,9 | 2,5 |
| 676,9 | 2,5 |
| 677,9 | 2,5 |
| 678,8 | 2,5 |
| 679,8 | 2,4 |
| 680,7 | 2,4 |
| 681,7 | 2,4 |
| 682,7 | 2,4 |
| 683,6 | 2,3 |
| 684,6 | 2,3 |
| 685,6 | 2,3 |
| 686,5 | 2,3 |
| 687,5 | 2,3 |
| 688,5 | 2,2 |
| 689,4 | 2,2 |
| 690,4 | 2,2 |
| 691,4 | 2,2 |
| 692,3 | 2,2 |
| 693,3 | 2,2 |
| 694,2 | 2,2 |
| 695,2 | 2,2 |
| 696,2 | 2,3 |
| 697,1 | 2,3 |
| 698,1 | 2,3 |
| 699,1 | 2,3 |
| 700,0 | 2,3 |
| 701,0 | 2,4 |
| 702,0 | 2,4 |
| 702,9 | 2,5 |
| 703,9 | 2,7 |
| 704,9 | 2,8 |
| 705,8 | 2,9 |
| 706,8 | 3,1 |
| 707,7 | 3,2 |
| 708,7 | 3,2 |
| 709,7 | 3,3 |
| 710,6 | 3,3 |
| 711,6 | 3,3 |
| 712,6 | 3,3 |
| 713,5 | 3,3 |
| 714,5 | 3,4 |
| 715,5 | 3,4 |
| 716,4 | 3,4 |
| 717,4 | 3,4 |
| 718,4 | 3,5 |
| 719,3 | 3,5 |
| 720,3 | 3,6 |
| 721,2 | 3,7 |
| 722,2 | 3,7 |
| 723,2 | 3,7 |
| 724,1 | 3,7 |
| 725,1 | 3,7 |
| 726,1 | 3,6 |
| 727,0 | 3,5 |
| 728,0 | 3,4 |
| 729,0 | 3,3 |
| 729,9 | 3,3 |
| 730,9 | 3,2 |
| 731,9 | 3,1 |
| 732,8 | 3,1 |
| 733,8 | 3,1 |
| 734,7 | 3,1 |
| 735,7 | 3,1 |
| 736,7 | 3,1 |
| 737,6 | 3,1 |
| 738,6 | 3,2 |
| 739,6 | 3,2 |
| 740,5 | 3,2 |
| 741,5 | 3,3 |
| 742,5 | 3,3 |
| 743,4 | 3,3 |
| 744,4 | 3,3 |
| 745,4 | 3,3 |
| 746,3 | 3,3 |
| 747,3 | 3,3 |
| 748,2 | 3,3 |
| 749,2 | 3,3 |
| 750,2 | 3,3 |
| 751,1 | 3,3 |
| 752,1 | 3,3 |
| 753,1 | 3,3 |
| 754,0 | 3,3 |
| 755,0 | 3,3 |
| 756,0 | 3,3 |
| 756,9 | 3,3 |
| 757,9 | 3,3 |
| 758,9 | 3,4 |
| 759,8 | 3,4 |
| 760,8 | 3,4 |
| 761,7 | 3,4 |
| 762,7 | 3,4 |
| 763,7 | 3,3 |
| 764,6 | 3,3 |
| 765,6 | 3,3 |
| 766,6 | 3,3 |
| 767,5 | 3,3 |
| 768,5 | 3,3 |
| 769,5 | 3,4 |
| 770,4 | 3,4 |
| 771,4 | 3,5 |
| 772,4 | 3,5 |
| 773,3 | 3,6 |
| 774,3 | 3,7 |
| 775,2 | 3,8 |
| 776,2 | 3,8 |
| 777,2 | 3,9 |
| 778,1 | 3,9 |
| 779,1 | 4,0 |
| 780,1 | 4,0 |
| 781,0 | 4,0 |
| 782,0 | 4,0 |
| 783,0 | 4,0 |
| 783,9 | 4,0 |
| 784,9 | 4,0 |
| 785,9 | 4,1 |
| 786,8 | 4,1 |
| 787,8 | 4,2 |
| 788,7 | 4,3 |
| 789,7 | 4,3 |
| 790,7 | 4,4 |
| 791,6 | 4,5 |
| 792,6 | 4,5 |
| 793,6 | 4,6 |
| 794,5 | 4,6 |
| 795,5 | 4,6 |
| 796,5 | 4,7 |
| 797,4 | 4,7 |
| 798,4 | 4,7 |
| 799,3 | 4,7 |
| 800,3 | 4,7 |
| 801,3 | 4,8 |
| 802,2 | 4,8 |
| 803,2 | 4,8 |
| 804,2 | 4,9 |
| 805,1 | 4,9 |
| 806,1 | 5,0 |
| 807,1 | 5,0 |
| 808,0 | 5,1 |
| 809,0 | 5,2 |
| 810,0 | 5,3 |
| 810,9 | 5,3 |
| 811,9 | 5,4 |
| 812,8 | 5,4 |
| 813,8 | 5,4 |
| 814,8 | 5,5 |
| 815,7 | 5,5 |
| 816,7 | 5,6 |
| 817,7 | 5,7 |
| 818,6 | 5,9 |
| 819,6 | 6,0 |
| 820,6 | 6,1 |
| 821,5 | 6,2 |
| 822,5 | 6,3 |
| 823,5 | 6,4 |
| 824,4 | 6,5 |
| 825,4 | 6,6 |
| 826,3 | 6,7 |
| 827,3 | 6,9 |
| 828,3 | 7,0 |
| 829,2 | 7,1 |
| 830,2 | 7,3 |
| 831,2 | 7,4 |
| 832,1 | 7,5 |
| 833,1 | 7,6 |
| 834,1 | 7,7 |
| 835,0 | 7,8 |
| 836,0 | 7,9 |
| 837,0 | 8,0 |
| 837,9 | 8,2 |
| 838,9 | 8,3 |
| 839,8 | 8,4 |
| 840,8 | 8,4 |
| 841,8 | 8,5 |
| 842,7 | 8,6 |
| 843,7 | 8,7 |
| 844,7 | 8,8 |
| 845,6 | 8,9 |
| 846,6 | 9,1 |
| 847,6 | 9,2 |
| 848,5 | 9,3 |
| 849,5 | 9,4 |
| 850,5 | 9,5 |
| 851,4 | 9,6 |
| 852,4 | 9,7 |
| 853,3 | 9,9 |
| 854,3 | 10,0 |
| 855,3 | 10,1 |
| 856,2 | 10,3 |
| 857,2 | 10,4 |
| 858,2 | 10,6 |
| 859,1 | 10,7 |
| 860,1 | 10,9 |
| 861,1 | 11,0 |
| 862,0 | 11,2 |
| 863,0 | 11,4 |
| 864,0 | 11,5 |
| 864,9 | 11,7 |
| 865,9 | 11,9 |
| 866,8 | 12,0 |
| 867,8 | 12,1 |
| 868,8 | 12,2 |
| 869,7 | 12,4 |
| 870,7 | 12,5 |
| 871,7 | 12,7 |
| 872,6 | 12,9 |
| 873,6 | 13,1 |
| 874,6 | 13,3 |
| 875,5 | 13,4 |
| 876,5 | 13,6 |
| 877,5 | 13,8 |
| 878,4 | 13,9 |
| 879,4 | 14,1 |
| 880,3 | 14,2 |
| 881,3 | 14,4 |
| 882,3 | 14,6 |
| 883,2 | 14,8 |
| 884,2 | 15,1 |
| 885,2 | 15,3 |
| 886,1 | 15,5 |
| 887,1 | 15,8 |
| 888,1 | 16,0 |
| 889,0 | 16,2 |
| 890,0 | 16,5 |
| 891,0 | 16,8 |
| 891,9 | 17,0 |
| 892,9 | 17,3 |
| 893,8 | 17,6 |
| 894,8 | 17,9 |
| 895,8 | 18,1 |
| 896,7 | 18,4 |
| 897,7 | 18,7 |
| 898,7 | 19,0 |
| 899,6 | 19,3 |
| 900,6 | 19,6 |
| 901,6 | 19,9 |
| 902,5 | 20,2 |
| 903,5 | 20,5 |
| 904,5 | 20,8 |
| 905,4 | 21,1 |
| 906,4 | 21,4 |
| 907,3 | 21,7 |
| 908,3 | 22,0 |
| 909,3 | 22,3 |
| 910,2 | 22,6 |
| 911,2 | 22,9 |
| 912,2 | 23,2 |
| 913,1 | 23,5 |
| 914,1 | 23,9 |
| 915,1 | 24,2 |
| 916,0 | 24,5 |
| 917,0 | 24,9 |
| 918,0 | 25,2 |
| 918,9 | 25,5 |
| 919,9 | 25,8 |
| 920,8 | 26,0 |
| 921,8 | 26,3 |
| 922,8 | 26,6 |
| 923,7 | 26,9 |
| 924,7 | 27,2 |
| 925,7 | 27,5 |
| 926,6 | 27,9 |
| 927,6 | 28,2 |
| 928,6 | 28,5 |
| 929,5 | 28,9 |
| 930,5 | 29,2 |
| 931,4 | 29,5 |
| 932,4 | 29,8 |
| 933,4 | 30,1 |
| 934,3 | 30,4 |
| 935,3 | 30,6 |
| 936,3 | 30,9 |
| 937,2 | 31,2 |
| 938,2 | 31,4 |
| 939,2 | 31,6 |
| 940,1 | 31,9 |
| 941,1 | 32,2 |
| 942,1 | 32,5 |
| 943,0 | 32,8 |
| 944,0 | 33,2 |
| 944,9 | 33,5 |
| 945,9 | 33,8 |
| 946,9 | 34,0 |
| 947,8 | 34,3 |
| 948,8 | 34,6 |
| 949,8 | 34,8 |
| 950,7 | 35,1 |
| 951,7 | 35,4 |
| 952,7 | 35,7 |
| 953,6 | 36,0 |
| 954,6 | 36,3 |
| 955,6 | 36,6 |
| 956,5 | 37,0 |
| 957,5 | 37,3 |
| 958,4 | 37,6 |
| 959,4 | 38,0 |
| 960,4 | 38,3 |
| 961,3 | 38,7 |
| 962,3 | 39,1 |
| 963,3 | 39,5 |
| 964,2 | 39,9 |
| 965,2 | 40,3 |
| 966,2 | 40,7 |
| 967,1 | 41,1 |
| 968,1 | 41,6 |
| 969,1 | 42,1 |
| 970,0 | 42,6 |
| 971,0 | 43,1 |
| 971,9 | 43,7 |
| 972,9 | 44,3 |
| 973,9 | 44,8 |
| 974,8 | 45,4 |
| 975,8 | 45,9 |
| 976,8 | 46,5 |
| 977,7 | 47,1 |
| 978,7 | 47,8 |
| 979,7 | 48,4 |
| 980,6 | 49,1 |
| 981,6 | 49,8 |
| 982,6 | 50,6 |
| 983,5 | 51,3 |
| 984,5 | 52,1 |
| 985,4 | 52,9 |
| 986,4 | 53,7 |
| 987,4 | 54,6 |
| 988,3 | 55,5 |
| 989,3 | 56,4 |
| 990,3 | 57,3 |
| 991,2 | 58,2 |
| 992,2 | 59,1 |
| 993,2 | 60,0 |
| 994,1 | 60,9 |
| 995,1 | 61,8 |
| 996,1 | 62,8 |
| 997,0 | 63,8 |
| 998,0 | 64,9 |
| 998,9 | 66,0 |
| 999,9 | 67,1 |
| 1000,9 | 68,3 |
| 1001,8 | 69,4 |
| 1002,8 | 70,5 |
| 1003,8 | 71,7 |
| 1004,7 | 72,8 |
| 1005,7 | 74,0 |
| 1006,7 | 75,2 |
| 1007,6 | 76,4 |
| 1008,6 | 77,6 |
| 1009,6 | 78,8 |
| 1010,5 | 80,0 |
| 1011,5 | 81,3 |
| 1012,4 | 82,4 |
| 1013,4 | 83,6 |
| 1014,4 | 84,8 |
| 1015,3 | 86,0 |
| 1016,3 | 87,3 |
| 1017,3 | 88,5 |
| 1018,2 | 89,8 |
| 1019,2 | 90,9 |
| 1020,2 | 91,9 |
| 1021,1 | 92,7 |
| 1022,1 | 93,5 |
| 1023,1 | 94,1 |
| 1024,0 | 94,6 |
| 1025,0 | 95,1 |
| 1025,9 | 95,5 |
| 1026,9 | 95,8 |
| 1027,9 | 96,1 |
| 1028,8 | 96,2 |
| 1029,8 | 96,2 |
| 1030,8 | 96,0 |
| 1031,7 | 95,7 |
| 1032,7 | 95,4 |
| 1033,7 | 94,9 |
| 1034,6 | 94,3 |
| 1035,6 | 93,7 |
| 1036,6 | 93,0 |
| 1037,5 | 92,2 |
| 1038,5 | 91,3 |
| 1039,4 | 90,2 |
| 1040,4 | 89,1 |
| 1041,4 | 87,8 |
| 1042,3 | 86,5 |
| 1043,3 | 85,1 |
| 1044,3 | 83,8 |
| 1045,2 | 82,4 |
| 1046,2 | 81,1 |
| 1047,2 | 79,7 |
| 1048,1 | 78,3 |
| 1049,1 | 77,0 |
| 1050,1 | 75,5 |
| 1051,0 | 74,1 |
| 1052,0 | 72,6 |
| 1052,9 | 71,2 |
| 1053,9 | 69,8 |
| 1054,9 | 68,4 |
| 1055,8 | 67,2 |
| 1056,8 | 65,9 |
| 1057,8 | 64,7 |
| 1058,7 | 63,4 |
| 1059,7 | 62,2 |
| 1060,7 | 61,1 |
| 1061,6 | 59,9 |
| 1062,6 | 58,9 |
| 1063,5 | 57,9 |
| 1064,5 | 57,0 |
| 1065,5 | 56,2 |
| 1066,4 | 55,5 |
| 1067,4 | 54,8 |
| 1068,4 | 54,3 |
| 1069,3 | 53,9 |
| 1070,3 | 53,5 |
| 1071,3 | 53,3 |
| 1072,2 | 53,1 |
| 1073,2 | 53,1 |
| 1074,2 | 53,1 |
| 1075,1 | 53,2 |
| 1076,1 | 53,5 |
| 1077,0 | 53,8 |
| 1078,0 | 54,3 |
| 1079,0 | 54,8 |
| 1079,9 | 55,4 |
| 1080,9 | 56,0 |
| 1081,9 | 56,8 |
| 1082,8 | 57,6 |
| 1083,8 | 58,5 |
| 1084,8 | 59,5 |
| 1085,7 | 60,5 |
| 1086,7 | 61,5 |
| 1087,7 | 62,6 |
| 1088,6 | 63,7 |
| 1089,6 | 64,9 |
| 1090,5 | 66,1 |
| 1091,5 | 67,3 |
| 1092,5 | 68,5 |
| 1093,4 | 69,7 |
| 1094,4 | 71,0 |
| 1095,4 | 72,3 |
| 1096,3 | 73,6 |
| 1097,3 | 74,9 |
| 1098,3 | 76,2 |
| 1099,2 | 77,5 |
| 1100,2 | 78,8 |
| 1101,2 | 80,1 |
| 1102,1 | 81,3 |
| 1103,1 | 82,6 |
| 1104,0 | 83,8 |
| 1105,0 | 85,0 |
| 1106,0 | 86,2 |
| 1106,9 | 87,3 |
| 1107,9 | 88,4 |
| 1108,9 | 89,5 |
| 1109,8 | 90,5 |
| 1110,8 | 91,5 |
| 1111,8 | 92,5 |
| 1112,7 | 93,3 |
| 1113,7 | 94,2 |
| 1114,7 | 94,9 |
| 1115,6 | 95,6 |
| 1116,6 | 96,3 |
| 1117,5 | 96,8 |
| 1118,5 | 97,3 |
| 1119,5 | 97,8 |
| 1120,4 | 98,1 |
| 1121,4 | 98,5 |
| 1122,4 | 98,7 |
| 1123,3 | 99,0 |
| 1124,3 | 99,1 |
| 1125,3 | 99,2 |
| 1126,2 | 99,3 |
| 1127,2 | 99,3 |
| 1128,2 | 99,3 |
| 1129,1 | 99,2 |
| 1130,1 | 99,1 |
| 1131,0 | 98,9 |
| 1132,0 | 98,7 |
| 1133,0 | 98,4 |
| 1133,9 | 98,2 |
| 1134,9 | 97,9 |
| 1135,9 | 97,6 |
| 1136,8 | 97,3 |
| 1137,8 | 97,0 |
| 1138,8 | 96,7 |
| 1139,7 | 96,4 |
| 1140,7 | 96,1 |
| 1141,7 | 95,8 |
| 1142,6 | 95,5 |
| 1143,6 | 95,2 |
| 1144,5 | 94,9 |
| 1145,5 | 94,6 |
| 1146,5 | 94,3 |
| 1147,4 | 94,0 |
| 1148,4 | 93,7 |
| 1149,4 | 93,4 |
| 1150,3 | 93,1 |
| 1151,3 | 92,9 |
| 1152,3 | 92,6 |
| 1153,2 | 92,4 |
| 1154,2 | 92,1 |
| 1155,2 | 91,9 |
| 1156,1 | 91,7 |
| 1157,1 | 91,5 |
| 1158,0 | 91,3 |
| 1159,0 | 91,1 |
| 1160,0 | 90,9 |
| 1160,9 | 90,8 |
| 1161,9 | 90,6 |
| 1162,9 | 90,5 |
| 1163,8 | 90,3 |
| 1164,8 | 90,2 |
| 1165,8 | 90,1 |
| 1166,7 | 90,0 |
| 1167,7 | 89,9 |
| 1168,7 | 89,8 |
| 1169,6 | 89,7 |
| 1170,6 | 89,7 |
| 1171,5 | 89,6 |
| 1172,5 | 89,6 |
| 1173,5 | 89,6 |
| 1174,4 | 89,6 |
| 1175,4 | 89,6 |
| 1176,4 | 89,6 |
| 1177,3 | 89,6 |
| 1178,3 | 89,6 |
| 1179,3 | 89,7 |
| 1180,2 | 89,8 |
| 1181,2 | 89,9 |
| 1182,2 | 89,9 |
| 1183,1 | 90,0 |
| 1184,1 | 90,2 |
| 1185,0 | 90,3 |
| 1186,0 | 90,4 |
| 1187,0 | 90,6 |
| 1187,9 | 90,7 |
| 1188,9 | 90,9 |
| 1189,9 | 91,1 |
| 1190,8 | 91,3 |
| 1191,8 | 91,5 |
| 1192,8 | 91,7 |
| 1193,7 | 92,0 |
| 1194,7 | 92,2 |
| 1195,6 | 92,4 |
| 1196,6 | 92,7 |
| 1197,6 | 92,9 |
| 1198,5 | 93,2 |
| 1199,5 | 93,4 |
| 1200,5 | 93,7 |
| 1201,4 | 94,0 |
| 1202,4 | 94,2 |
| 1203,4 | 94,5 |
| 1204,3 | 94,7 |
| 1205,3 | 95,0 |
| 1206,3 | 95,2 |
| 1207,2 | 95,4 |
| 1208,2 | 95,7 |
| 1209,1 | 95,9 |
| 1210,1 | 96,1 |
| 1211,1 | 96,4 |
| 1212,0 | 96,6 |
| 1213,0 | 96,8 |
| 1214,0 | 97,0 |
| 1214,9 | 97,2 |
| 1215,9 | 97,4 |
| 1216,9 | 97,6 |
| 1217,8 | 97,8 |
| 1218,8 | 98,0 |
| 1219,8 | 98,2 |
| 1220,7 | 98,4 |
| 1221,7 | 98,6 |
| 1222,6 | 98,8 |
| 1223,6 | 99,0 |
| 1224,6 | 99,1 |
| 1225,5 | 99,3 |
| 1226,5 | 99,5 |
| 1227,5 | 99,6 |
| 1228,4 | 99,8 |
| 1229,4 | 100,0 |
| 1230,4 | 100,1 |
| 1231,3 | 100,3 |
| 1232,3 | 100,5 |
| 1233,3 | 100,6 |
| 1234,2 | 100,7 |
| 1235,2 | 100,9 |
| 1236,1 | 101,0 |
| 1237,1 | 101,2 |
| 1238,1 | 101,3 |
| 1239,0 | 101,4 |
| 1240,0 | 101,5 |
| 1241,0 | 101,6 |
| 1241,9 | 101,8 |
| 1242,9 | 101,9 |
| 1243,9 | 102,0 |
| 1244,8 | 102,1 |
| 1245,8 | 102,2 |
| 1246,8 | 102,3 |
| 1247,7 | 102,4 |
| 1248,7 | 102,5 |
| 1249,6 | 102,6 |
| 1250,6 | 102,6 |
| 1251,6 | 102,7 |
| 1252,5 | 102,8 |
| 1253,5 | 102,9 |
| 1254,5 | 102,9 |
| 1255,4 | 103,0 |
| 1256,4 | 103,1 |
| 1257,4 | 103,1 |
| 1258,3 | 103,2 |
| 1259,3 | 103,3 |
| 1260,3 | 103,3 |
| 1261,2 | 103,4 |
| 1262,2 | 103,4 |
| 1263,1 | 103,4 |
| 1264,1 | 103,5 |
| 1265,1 | 103,5 |
| 1266,0 | 103,6 |
| 1267,0 | 103,6 |
| 1268,0 | 103,6 |
| 1268,9 | 103,6 |
| 1269,9 | 103,6 |
| 1270,9 | 103,7 |
| 1271,8 | 103,7 |
| 1272,8 | 103,7 |
| 1273,8 | 103,7 |
| 1274,7 | 103,6 |
| 1275,7 | 103,6 |
| 1276,6 | 103,6 |
| 1277,6 | 103,5 |
| 1278,6 | 103,5 |
| 1279,5 | 103,4 |
| 1280,5 | 103,4 |
| 1281,5 | 103,3 |
| 1282,4 | 103,2 |
| 1283,4 | 103,1 |
| 1284,4 | 103,0 |
| 1285,3 | 102,9 |
| 1286,3 | 102,9 |
| 1287,3 | 102,8 |
| 1288,2 | 102,8 |
| 1289,2 | 102,8 |
| 1290,1 | 102,7 |
| 1291,1 | 102,7 |
| 1292,1 | 102,7 |
| 1293,0 | 102,7 |
| 1294,0 | 102,7 |
| 1295,0 | 102,7 |
| 1295,9 | 102,7 |
| 1296,9 | 102,6 |
| 1297,9 | 102,6 |
| 1298,8 | 102,6 |
| 1299,8 | 102,6 |
| 1300,8 | 102,5 |
| 1301,7 | 102,5 |
| 1302,7 | 102,4 |
| 1303,6 | 102,3 |
| 1304,6 | 102,1 |
| 1305,6 | 102,0 |
| 1306,5 | 101,8 |
| 1307,5 | 101,6 |
| 1308,5 | 101,3 |
| 1309,4 | 100,9 |
| 1310,4 | 100,5 |
| 1311,4 | 100,0 |
| 1312,3 | 99,4 |
| 1313,3 | 98,7 |
| 1314,3 | 98,0 |
| 1315,2 | 97,1 |
| 1316,2 | 96,2 |
| 1317,1 | 95,2 |
| 1318,1 | 94,1 |
| 1319,1 | 92,8 |
| 1320,0 | 91,5 |
| 1321,0 | 90,1 |
| 1322,0 | 88,5 |
| 1322,9 | 86,9 |
| 1323,9 | 85,2 |
| 1324,9 | 83,3 |
| 1325,8 | 81,4 |
| 1326,8 | 79,3 |
| 1327,7 | 77,1 |
| 1328,7 | 74,8 |
| 1329,7 | 72,4 |
| 1330,6 | 70,0 |
| 1331,6 | 67,5 |
| 1332,6 | 65,0 |
| 1333,5 | 62,5 |
| 1334,5 | 59,9 |
| 1335,5 | 57,3 |
| 1336,4 | 54,7 |
| 1337,4 | 52,1 |
| 1338,4 | 49,5 |
| 1339,3 | 47,0 |
| 1340,3 | 44,5 |
| 1341,2 | 42,0 |
| 1342,2 | 39,7 |
| 1343,2 | 37,4 |
| 1344,1 | 35,2 |
| 1345,1 | 33,1 |
| 1346,1 | 31,1 |
| 1347,0 | 29,2 |
| 1348,0 | 27,4 |
| 1349,0 | 25,8 |
| 1349,9 | 24,2 |
| 1350,9 | 22,8 |
| 1351,9 | 21,6 |
| 1352,8 | 20,5 |
| 1353,8 | 19,5 |
| 1354,7 | 18,6 |
| 1355,7 | 17,8 |
| 1356,7 | 17,1 |
| 1357,6 | 16,6 |
| 1358,6 | 16,1 |
| 1359,6 | 15,7 |
| 1360,5 | 15,4 |
| 1361,5 | 15,2 |
| 1362,5 | 15,0 |
| 1363,4 | 14,9 |
| 1364,4 | 14,9 |
| 1365,4 | 14,9 |
| 1366,3 | 15,0 |
| 1367,3 | 15,1 |
| 1368,2 | 15,3 |
| 1369,2 | 15,5 |
| 1370,2 | 15,7 |
| 1371,1 | 16,0 |
| 1372,1 | 16,3 |
| 1373,1 | 16,6 |
| 1374,0 | 17,0 |
| 1375,0 | 17,3 |
| 1376,0 | 17,7 |
| 1376,9 | 18,1 |
| 1377,9 | 18,4 |
| 1378,9 | 18,8 |
| 1379,8 | 19,2 |
| 1380,8 | 19,6 |
| 1381,7 | 20,0 |
| 1382,7 | 20,4 |
| 1383,7 | 20,8 |
| 1384,6 | 21,2 |
| 1385,6 | 21,6 |
| 1386,6 | 21,9 |
| 1387,5 | 22,3 |
| 1388,5 | 22,7 |
| 1389,5 | 23,0 |
| 1390,4 | 23,4 |
| 1391,4 | 23,7 |
| 1392,4 | 24,1 |
| 1393,3 | 24,4 |
| 1394,3 | 24,7 |
| 1395,2 | 25,1 |
| 1396,2 | 25,4 |
| 1397,2 | 25,8 |
| 1398,1 | 26,2 |
| 1399,1 | 26,5 |
| 1400,1 | 26,9 |
| 1401,0 | 27,3 |
| 1402,0 | 27,7 |
| 1403,0 | 28,2 |
| 1403,9 | 28,6 |
| 1404,9 | 29,1 |
| 1405,9 | 29,6 |
| 1406,8 | 30,1 |
| 1407,8 | 30,7 |
| 1408,7 | 31,3 |
| 1409,7 | 32,0 |
| 1410,7 | 32,7 |
| 1411,6 | 33,4 |
| 1412,6 | 34,2 |
| 1413,6 | 35,0 |
| 1414,5 | 35,8 |
| 1415,5 | 36,7 |
| 1416,5 | 37,6 |
| 1417,4 | 38,5 |
| 1418,4 | 39,5 |
| 1419,4 | 40,5 |
| 1420,3 | 41,5 |
| 1421,3 | 42,6 |
| 1422,2 | 43,7 |
| 1423,2 | 44,8 |
| 1424,2 | 46,0 |
| 1425,1 | 47,2 |
| 1426,1 | 48,4 |
| 1427,1 | 49,7 |
| 1428,0 | 51,0 |
| 1429,0 | 52,3 |
| 1430,0 | 53,7 |
| 1430,9 | 55,0 |
| 1431,9 | 56,4 |
| 1432,9 | 57,7 |
| 1433,8 | 59,1 |
| 1434,8 | 60,4 |
| 1435,7 | 61,8 |
| 1436,7 | 63,1 |
| 1437,7 | 64,5 |
| 1438,6 | 65,9 |
| 1439,6 | 67,3 |
| 1440,6 | 68,6 |
| 1441,5 | 70,0 |
| 1442,5 | 71,3 |
| 1443,5 | 72,6 |
| 1444,4 | 73,8 |
| 1445,4 | 75,1 |
| 1446,4 | 76,3 |
| 1447,3 | 77,6 |
| 1448,3 | 78,8 |
| 1449,2 | 79,9 |
| 1450,2 | 81,0 |
| 1451,2 | 82,1 |
| 1452,1 | 83,1 |
| 1453,1 | 84,1 |
| 1454,1 | 85,0 |
| 1455,0 | 85,9 |
| 1456,0 | 86,8 |
| 1457,0 | 87,6 |
| 1457,9 | 88,4 |
| 1458,9 | 89,1 |
| 1459,8 | 89,8 |
| 1460,8 | 90,5 |
| 1461,8 | 91,1 |
| 1462,7 | 91,6 |
| 1463,7 | 92,1 |
| 1464,7 | 92,6 |
| 1465,6 | 93,0 |
| 1466,6 | 93,5 |
| 1467,6 | 93,9 |
| 1468,5 | 94,2 |
| 1469,5 | 94,6 |
| 1470,5 | 94,9 |
| 1471,4 | 95,1 |
| 1472,4 | 95,4 |
| 1473,3 | 95,6 |
| 1474,3 | 95,8 |
| 1475,3 | 95,9 |
| 1476,2 | 96,1 |
| 1477,2 | 96,2 |
| 1478,2 | 96,3 |
| 1479,1 | 96,4 |
| 1480,1 | 96,4 |
| 1481,1 | 96,5 |
| 1482,0 | 96,6 |
| 1483,0 | 96,6 |
| 1484,0 | 96,7 |
| 1484,9 | 96,7 |
| 1485,9 | 96,8 |
| 1486,8 | 96,8 |
| 1487,8 | 96,8 |
| 1488,8 | 96,9 |
| 1489,7 | 96,9 |
| 1490,7 | 96,9 |
| 1491,7 | 96,9 |
| 1492,6 | 96,9 |
| 1493,6 | 96,9 |
| 1494,6 | 96,8 |
| 1495,5 | 96,8 |
| 1496,5 | 96,7 |
| 1497,5 | 96,6 |
| 1498,4 | 96,6 |
| 1499,4 | 96,5 |
| 1500,3 | 96,5 |
| 1501,3 | 96,6 |
| 1502,3 | 96,6 |
| 1503,2 | 96,6 |
| 1504,2 | 96,7 |
| 1505,2 | 96,7 |
| 1506,1 | 96,7 |
| 1507,1 | 96,7 |
| 1508,1 | 96,7 |
| 1509,0 | 96,7 |
| 1510,0 | 96,8 |
| 1511,0 | 96,8 |
| 1511,9 | 96,8 |
| 1512,9 | 96,8 |
| 1513,8 | 96,9 |
| 1514,8 | 96,9 |
| 1515,8 | 97,0 |
| 1516,7 | 97,0 |
| 1517,7 | 97,1 |
| 1518,7 | 97,2 |
| 1519,6 | 97,4 |
| 1520,6 | 97,5 |
| 1521,6 | 97,6 |
| 1522,5 | 97,8 |
| 1523,5 | 97,9 |
| 1524,5 | 97,9 |
| 1525,4 | 98,0 |
| 1526,4 | 98,0 |
| 1527,3 | 98,1 |
| 1528,3 | 98,1 |
| 1529,3 | 98,0 |
| 1530,2 | 97,9 |
| 1531,2 | 97,9 |
| 1532,2 | 97,8 |
| 1533,1 | 97,8 |
| 1534,1 | 97,7 |
| 1535,1 | 97,7 |
| 1536,0 | 97,6 |
| 1537,0 | 97,6 |
| 1538,0 | 97,5 |
| 1538,9 | 97,4 |
| 1539,9 | 97,3 |
| 1540,8 | 97,1 |
| 1541,8 | 96,9 |
| 1542,8 | 96,7 |
| 1543,7 | 96,5 |
| 1544,7 | 96,3 |
| 1545,7 | 96,0 |
| 1546,6 | 95,8 |
| 1547,6 | 95,4 |
| 1548,6 | 95,1 |
| 1549,5 | 94,7 |
| 1550,5 | 94,4 |
| 1551,5 | 94,1 |
| 1552,4 | 93,8 |
| 1553,4 | 93,5 |
| 1554,3 | 93,3 |
| 1555,3 | 93,0 |
| 1556,3 | 92,6 |
| 1557,2 | 92,3 |
| 1558,2 | 91,9 |
| 1559,2 | 91,5 |
| 1560,1 | 91,0 |
| 1561,1 | 90,6 |
| 1562,1 | 90,1 |
| 1563,0 | 89,7 |
| 1564,0 | 89,1 |
| 1565,0 | 88,6 |
| 1565,9 | 88,1 |
| 1566,9 | 87,5 |
| 1567,8 | 86,9 |
| 1568,8 | 86,3 |
| 1569,8 | 85,8 |
| 1570,7 | 85,2 |
| 1571,7 | 84,6 |
| 1572,7 | 84,0 |
| 1573,6 | 83,4 |
| 1574,6 | 82,8 |
| 1575,6 | 82,1 |
| 1576,5 | 81,3 |
| 1577,5 | 80,6 |
| 1578,5 | 79,8 |
| 1579,4 | 79,0 |
| 1580,4 | 78,2 |
| 1581,3 | 77,4 |
| 1582,3 | 76,5 |
| 1583,3 | 75,7 |
| 1584,2 | 74,8 |
| 1585,2 | 73,9 |
| 1586,2 | 72,9 |
| 1587,1 | 72,0 |
| 1588,1 | 71,0 |
| 1589,1 | 70,1 |
| 1590,0 | 69,1 |
| 1591,0 | 68,1 |
| 1591,9 | 67,1 |
| 1592,9 | 66,0 |
| 1593,9 | 65,0 |
| 1594,8 | 63,9 |
| 1595,8 | 62,8 |
| 1596,8 | 61,7 |
| 1597,7 | 60,6 |
| 1598,7 | 59,5 |
| 1599,7 | 58,4 |
| 1600,6 | 57,3 |
| 1601,6 | 56,2 |
| 1602,6 | 55,1 |
| 1603,5 | 54,0 |
| 1604,5 | 52,9 |
| 1605,4 | 51,8 |
| 1606,4 | 50,7 |
| 1607,4 | 49,6 |
| 1608,3 | 48,6 |
| 1609,3 | 47,5 |
| 1610,3 | 46,5 |
| 1611,2 | 45,5 |
| 1612,2 | 44,5 |
| 1613,2 | 43,6 |
| 1614,1 | 42,6 |
| 1615,1 | 41,7 |
| 1616,1 | 40,8 |
| 1617,0 | 40,0 |
| 1618,0 | 39,2 |
| 1618,9 | 38,4 |
| 1619,9 | 37,6 |
| 1620,9 | 36,9 |
| 1621,8 | 36,2 |
| 1622,8 | 35,6 |
| 1623,8 | 34,9 |
| 1624,7 | 34,3 |
| 1625,7 | 33,7 |
| 1626,7 | 33,2 |
| 1627,6 | 32,7 |
| 1628,6 | 32,2 |
| 1629,6 | 31,8 |
| 1630,5 | 31,5 |
| 1631,5 | 31,2 |
| 1632,4 | 30,9 |
| 1633,4 | 30,6 |
| 1634,4 | 30,3 |
| 1635,3 | 30,1 |
| 1636,3 | 29,9 |
| 1637,3 | 29,8 |
| 1638,2 | 29,7 |
| 1639,2 | 29,6 |
| 1640,2 | 29,5 |
| 1641,1 | 29,5 |
| 1642,1 | 29,5 |
| 1643,1 | 29,5 |
| 1644,0 | 29,6 |
| 1645,0 | 29,7 |
| 1645,9 | 29,8 |
| 1646,9 | 29,9 |
| 1647,9 | 30,1 |
| 1648,8 | 30,3 |
| 1649,8 | 30,5 |
| 1650,8 | 30,8 |
| 1651,7 | 31,1 |
| 1652,7 | 31,4 |
| 1653,7 | 31,7 |
| 1654,6 | 32,0 |
| 1655,6 | 32,3 |
| 1656,6 | 32,7 |
| 1657,5 | 33,1 |
| 1658,5 | 33,5 |
| 1659,4 | 34,0 |
| 1660,4 | 34,4 |
| 1661,4 | 34,9 |
| 1662,3 | 35,4 |
| 1663,3 | 35,9 |
| 1664,3 | 36,5 |
| 1665,2 | 37,0 |
| 1666,2 | 37,6 |
| 1667,2 | 38,2 |
| 1668,1 | 38,7 |
| 1669,1 | 39,3 |
| 1670,1 | 39,9 |
| 1671,0 | 40,5 |
| 1672,0 | 41,2 |
| 1672,9 | 41,8 |
| 1673,9 | 42,5 |
| 1674,9 | 43,2 |
| 1675,8 | 44,0 |
| 1676,8 | 44,7 |
| 1677,8 | 45,4 |
| 1678,7 | 46,2 |
| 1679,7 | 46,9 |
| 1680,7 | 47,7 |
| 1681,6 | 48,5 |
| 1682,6 | 49,3 |
| 1683,6 | 50,0 |
| 1684,5 | 50,8 |
| 1685,5 | 51,6 |
| 1686,4 | 52,4 |
| 1687,4 | 53,2 |
| 1688,4 | 54,0 |
| 1689,3 | 54,8 |
| 1690,3 | 55,6 |
| 1691,3 | 56,4 |
| 1692,2 | 57,3 |
| 1693,2 | 58,1 |
| 1694,2 | 58,9 |
| 1695,1 | 59,8 |
| 1696,1 | 60,6 |
| 1697,1 | 61,5 |
| 1698,0 | 62,3 |
| 1699,0 | 63,1 |
| 1699,9 | 63,9 |
| 1700,9 | 64,7 |
| 1701,9 | 65,5 |
| 1702,8 | 66,2 |
| 1703,8 | 67,0 |
| 1704,8 | 67,7 |
| 1705,7 | 68,5 |
| 1706,7 | 69,3 |
| 1707,7 | 70,0 |
| 1708,6 | 70,8 |
| 1709,6 | 71,5 |
| 1710,6 | 72,3 |
| 1711,5 | 73,0 |
| 1712,5 | 73,7 |
| 1713,4 | 74,4 |
| 1714,4 | 75,1 |
| 1715,4 | 75,8 |
| 1716,3 | 76,5 |
| 1717,3 | 77,1 |
| 1718,3 | 77,8 |
| 1719,2 | 78,4 |
| 1720,2 | 78,9 |
| 1721,2 | 79,5 |
| 1722,1 | 80,0 |
| 1723,1 | 80,6 |
| 1724,0 | 81,1 |
| 1725,0 | 81,6 |
| 1726,0 | 82,1 |
| 1726,9 | 82,6 |
| 1727,9 | 83,1 |
| 1728,9 | 83,6 |
| 1729,8 | 84,1 |
| 1730,8 | 84,5 |
| 1731,8 | 84,9 |
| 1732,7 | 85,4 |
| 1733,7 | 85,7 |
| 1734,7 | 86,1 |
| 1735,6 | 86,5 |
| 1736,6 | 86,8 |
| 1737,5 | 87,1 |
| 1738,5 | 87,4 |
| 1739,5 | 87,7 |
| 1740,4 | 88,0 |
| 1741,4 | 88,3 |
| 1742,4 | 88,6 |
| 1743,3 | 89,0 |
| 1744,3 | 89,3 |
| 1745,3 | 89,6 |
| 1746,2 | 89,9 |
| 1747,2 | 90,2 |
| 1748,2 | 90,5 |
| 1749,1 | 90,8 |
| 1750,1 | 91,1 |
| 1751,0 | 91,4 |
| 1752,0 | 91,7 |
| 1753,0 | 91,9 |
| 1753,9 | 92,2 |
| 1754,9 | 92,5 |
| 1755,9 | 92,8 |
| 1756,8 | 93,1 |
| 1757,8 | 93,3 |
| 1758,8 | 93,6 |
| 1759,7 | 93,9 |
| 1760,7 | 94,1 |
| 1761,7 | 94,4 |
| 1762,6 | 94,6 |
| 1763,6 | 94,9 |
| 1764,5 | 95,1 |
| 1765,5 | 95,4 |
| 1766,5 | 95,6 |
| 1767,4 | 95,8 |
| 1768,4 | 96,0 |
| 1769,4 | 96,3 |
| 1770,3 | 96,5 |
| 1771,3 | 96,7 |
| 1772,3 | 96,9 |
| 1773,2 | 97,1 |
| 1774,2 | 97,2 |
| 1775,2 | 97,4 |
| 1776,1 | 97,6 |
| 1777,1 | 97,8 |
| 1778,0 | 97,9 |
| 1779,0 | 98,0 |
| 1780,0 | 98,2 |
| 1780,9 | 98,3 |
| 1781,9 | 98,4 |
| 1782,9 | 98,5 |
| 1783,8 | 98,6 |
| 1784,8 | 98,7 |
| 1785,8 | 98,8 |
| 1786,7 | 98,9 |
| 1787,7 | 99,0 |
| 1788,7 | 99,1 |
| 1789,6 | 99,2 |
| 1790,6 | 99,2 |
| 1791,5 | 99,3 |
| 1792,5 | 99,3 |
| 1793,5 | 99,4 |
| 1794,4 | 99,5 |
| 1795,4 | 99,5 |
| 1796,4 | 99,6 |
| 1797,3 | 99,6 |
| 1798,3 | 99,6 |
| 1799,3 | 99,6 |
| 1800,2 | 99,7 |
| 1801,2 | 99,7 |
| 1802,2 | 99,7 |
| 1803,1 | 99,7 |
| 1804,1 | 99,7 |
| 1805,0 | 99,8 |
| 1806,0 | 99,8 |
| 1807,0 | 99,8 |
| 1807,9 | 99,9 |
| 1808,9 | 99,9 |
| 1809,9 | 99,9 |
| 1810,8 | 99,9 |
| 1811,8 | 99,9 |
| 1812,8 | 99,9 |
| 1813,7 | 99,9 |
| 1814,7 | 99,9 |
| 1815,7 | 99,9 |
| 1816,6 | 99,8 |
| 1817,6 | 99,8 |
| 1818,5 | 99,7 |
| 1819,5 | 99,7 |
| 1820,5 | 99,7 |
| 1821,4 | 99,6 |
| 1822,4 | 99,6 |
| 1823,4 | 99,6 |
| 1824,3 | 99,5 |
| 1825,3 | 99,5 |
| 1826,3 | 99,5 |
| 1827,2 | 99,4 |
| 1828,2 | 99,4 |
| 1829,2 | 99,3 |
| 1830,1 | 99,3 |
| 1831,1 | 99,2 |
| 1832,0 | 99,2 |
| 1833,0 | 99,1 |
| 1834,0 | 99,0 |
| 1834,9 | 98,9 |
| 1835,9 | 98,9 |
| 1836,9 | 98,8 |
| 1837,8 | 98,8 |
| 1838,8 | 98,7 |
| 1839,8 | 98,7 |
| 1840,7 | 98,7 |
| 1841,7 | 98,7 |
| 1842,7 | 98,6 |
| 1843,6 | 98,6 |
| 1844,6 | 98,6 |
| 1845,5 | 98,6 |
| 1846,5 | 98,5 |
| 1847,5 | 98,5 |
| 1848,4 | 98,5 |
| 1849,4 | 98,4 |
| 1850,4 | 98,4 |
| 1851,3 | 98,4 |
| 1852,3 | 98,3 |
| 1853,3 | 98,3 |
| 1854,2 | 98,3 |
| 1855,2 | 98,2 |
| 1856,1 | 98,2 |
| 1857,1 | 98,2 |
| 1858,1 | 98,1 |
| 1859,0 | 98,1 |
| 1860,0 | 98,1 |
| 1861,0 | 98,0 |
| 1861,9 | 98,0 |
| 1862,9 | 97,9 |
| 1863,9 | 97,9 |
| 1864,8 | 97,8 |
| 1865,8 | 97,8 |
| 1866,8 | 97,7 |
| 1867,7 | 97,7 |
| 1868,7 | 97,6 |
| 1869,6 | 97,6 |
| 1870,6 | 97,5 |
| 1871,6 | 97,5 |
| 1872,5 | 97,4 |
| 1873,5 | 97,4 |
| 1874,5 | 97,3 |
| 1875,4 | 97,2 |
| 1876,4 | 97,2 |
| 1877,4 | 97,1 |
| 1878,3 | 97,0 |
| 1879,3 | 96,9 |
| 1880,3 | 96,9 |
| 1881,2 | 96,8 |
| 1882,2 | 96,8 |
| 1883,1 | 96,7 |
| 1884,1 | 96,6 |
| 1885,1 | 96,6 |
| 1886,0 | 96,5 |
| 1887,0 | 96,4 |
| 1888,0 | 96,3 |
| 1888,9 | 96,2 |
| 1889,9 | 96,2 |
| 1890,9 | 96,1 |
| 1891,8 | 96,0 |
| 1892,8 | 95,9 |
| 1893,8 | 95,8 |
| 1894,7 | 95,7 |
| 1895,7 | 95,6 |
| 1896,6 | 95,5 |
| 1897,6 | 95,4 |
| 1898,6 | 95,3 |
| 1899,5 | 95,2 |
| 1900,5 | 95,1 |
| 1901,5 | 95,0 |
| 1902,4 | 94,9 |
| 1903,4 | 94,8 |
| 1904,4 | 94,7 |
| 1905,3 | 94,6 |
| 1906,3 | 94,5 |
| 1907,3 | 94,4 |
| 1908,2 | 94,3 |
| 1909,2 | 94,2 |
| 1910,1 | 94,1 |
| 1911,1 | 94,0 |
| 1912,1 | 93,9 |
| 1913,0 | 93,7 |
| 1914,0 | 93,6 |
| 1915,0 | 93,5 |
| 1915,9 | 93,4 |
| 1916,9 | 93,3 |
| 1917,9 | 93,2 |
| 1918,8 | 93,0 |
| 1919,8 | 92,9 |
| 1920,8 | 92,8 |
| 1921,7 | 92,7 |
| 1922,7 | 92,6 |
| 1923,6 | 92,5 |
| 1924,6 | 92,4 |
| 1925,6 | 92,2 |
| 1926,5 | 92,1 |
| 1927,5 | 92,0 |
| 1928,5 | 91,9 |
| 1929,4 | 91,7 |
| 1930,4 | 91,6 |
| 1931,4 | 91,5 |
| 1932,3 | 91,4 |
| 1933,3 | 91,2 |
| 1934,3 | 91,1 |
| 1935,2 | 91,0 |
| 1936,2 | 90,9 |
| 1937,1 | 90,8 |
| 1938,1 | 90,6 |
| 1939,1 | 90,5 |
| 1940,0 | 90,4 |
| 1941,0 | 90,3 |
| 1942,0 | 90,1 |
| 1942,9 | 90,0 |
| 1943,9 | 89,9 |
| 1944,9 | 89,8 |
| 1945,8 | 89,6 |
| 1946,8 | 89,5 |
| 1947,8 | 89,4 |
| 1948,7 | 89,3 |
| 1949,7 | 89,1 |
| 1950,6 | 89,0 |
| 1951,6 | 88,9 |
| 1952,6 | 88,8 |
| 1953,5 | 88,6 |
| 1954,5 | 88,5 |
| 1955,5 | 88,4 |
| 1956,4 | 88,3 |
| 1957,4 | 88,2 |
| 1958,4 | 88,0 |
| 1959,3 | 87,9 |
| 1960,3 | 87,8 |
| 1961,3 | 87,7 |
| 1962,2 | 87,6 |
| 1963,2 | 87,4 |
| 1964,1 | 87,3 |
| 1965,1 | 87,2 |
| 1966,1 | 87,1 |
| 1967,0 | 87,0 |
| 1968,0 | 86,8 |
| 1969,0 | 86,7 |
| 1969,9 | 86,6 |
| 1970,9 | 86,5 |
| 1971,9 | 86,4 |
| 1972,8 | 86,3 |
| 1973,8 | 86,1 |
| 1974,8 | 86,0 |
| 1975,7 | 85,9 |
| 1976,7 | 85,8 |
| 1977,6 | 85,7 |
| 1978,6 | 85,5 |
| 1979,6 | 85,4 |
| 1980,5 | 85,3 |
| 1981,5 | 85,2 |
| 1982,5 | 85,1 |
| 1983,4 | 85,0 |
| 1984,4 | 84,8 |
| 1985,4 | 84,7 |
| 1986,3 | 84,6 |
| 1987,3 | 84,5 |
| 1988,2 | 84,4 |
| 1989,2 | 84,3 |
| 1990,2 | 84,2 |
| 1991,1 | 84,1 |
| 1992,1 | 83,9 |
| 1993,1 | 83,8 |
| 1994,0 | 83,7 |
| 1995,0 | 83,6 |
| 1996,0 | 83,5 |
| 1996,9 | 83,4 |
| 1997,9 | 83,3 |
| 1998,9 | 83,1 |
| 1999,8 | 83,0 |
| 2000,8 | 82,9 |
| 2001,7 | 82,8 |
| 2002,7 | 82,7 |
| 2003,7 | 82,6 |
| 2004,6 | 82,5 |
| 2005,6 | 82,4 |
| 2006,6 | 82,2 |
| 2007,5 | 82,1 |
| 2008,5 | 82,0 |
| 2009,5 | 81,9 |
| 2010,4 | 81,8 |
| 2011,4 | 81,6 |
| 2012,4 | 81,5 |
| 2013,3 | 81,4 |
| 2014,3 | 81,3 |
| 2015,2 | 81,2 |
| 2016,2 | 81,1 |
| 2017,2 | 80,9 |
| 2018,1 | 80,8 |
| 2019,1 | 80,7 |
| 2020,1 | 80,6 |
| 2021,0 | 80,5 |
| 2022,0 | 80,4 |
| 2023,0 | 80,2 |
| 2023,9 | 80,1 |
| 2024,9 | 80,0 |
| 2025,9 | 79,9 |
| 2026,8 | 79,8 |
| 2027,8 | 79,7 |
| 2028,7 | 79,6 |
| 2029,7 | 79,5 |
| 2030,7 | 79,3 |
| 2031,6 | 79,2 |
| 2032,6 | 79,1 |
| 2033,6 | 79,0 |
| 2034,5 | 78,9 |
| 2035,5 | 78,8 |
| 2036,5 | 78,7 |
| 2037,4 | 78,6 |
| 2038,4 | 78,5 |
| 2039,4 | 78,4 |
| 2040,3 | 78,3 |
| 2041,3 | 78,2 |
| 2042,2 | 78,1 |
| 2043,2 | 78,0 |
| 2044,2 | 77,9 |
| 2045,1 | 77,8 |
| 2046,1 | 77,7 |
| 2047,1 | 77,6 |
| 2048,0 | 77,5 |
| 2049,0 | 77,4 |
| 2050,0 | 77,3 |
| 2050,9 | 77,3 |
| 2051,9 | 77,2 |
| 2052,9 | 77,1 |
| 2053,8 | 77,0 |
| 2054,8 | 76,9 |
| 2055,7 | 76,8 |
| 2056,7 | 76,7 |
| 2057,7 | 76,7 |
| 2058,6 | 76,6 |
| 2059,6 | 76,5 |
| 2060,6 | 76,4 |
| 2061,5 | 76,4 |
| 2062,5 | 76,3 |
| 2063,5 | 76,2 |
| 2064,4 | 76,2 |
| 2065,4 | 76,1 |
| 2066,4 | 76,0 |
| 2067,3 | 76,0 |
| 2068,3 | 75,9 |
| 2069,2 | 75,8 |
| 2070,2 | 75,8 |
| 2071,2 | 75,7 |
| 2072,1 | 75,7 |
| 2073,1 | 75,6 |
| 2074,1 | 75,6 |
| 2075,0 | 75,5 |
| 2076,0 | 75,5 |
| 2077,0 | 75,5 |
| 2077,9 | 75,4 |
| 2078,9 | 75,4 |
| 2079,9 | 75,3 |
| 2080,8 | 75,3 |
| 2081,8 | 75,3 |
| 2082,7 | 75,2 |
| 2083,7 | 75,2 |
| 2084,7 | 75,2 |
| 2085,6 | 75,2 |
| 2086,6 | 75,1 |
| 2087,6 | 75,1 |
| 2088,5 | 75,1 |
| 2089,5 | 75,1 |
| 2090,5 | 75,1 |
| 2091,4 | 75,0 |
| 2092,4 | 75,0 |
| 2093,4 | 75,0 |
| 2094,3 | 75,0 |
| 2095,3 | 75,0 |
| 2096,2 | 75,0 |
| 2097,2 | 75,0 |
| 2098,2 | 75,0 |
| 2099,1 | 75,0 |
| 2100,1 | 75,0 |
| 2101,1 | 75,0 |
| 2102,0 | 75,0 |
| 2103,0 | 75,0 |
| 2104,0 | 75,1 |
| 2104,9 | 75,1 |
| 2105,9 | 75,1 |
| 2106,9 | 75,1 |
| 2107,8 | 75,1 |
| 2108,8 | 75,1 |
| 2109,7 | 75,2 |
| 2110,7 | 75,2 |
| 2111,7 | 75,2 |
| 2112,6 | 75,2 |
| 2113,6 | 75,3 |
| 2114,6 | 75,3 |
| 2115,5 | 75,3 |
| 2116,5 | 75,4 |
| 2117,5 | 75,4 |
| 2118,4 | 75,5 |
| 2119,4 | 75,5 |
| 2120,3 | 75,5 |
| 2121,3 | 75,6 |
| 2122,3 | 75,6 |
| 2123,2 | 75,7 |
| 2124,2 | 75,7 |
| 2125,2 | 75,8 |
| 2126,1 | 75,8 |
| 2127,1 | 75,9 |
| 2128,1 | 75,9 |
| 2129,0 | 76,0 |
| 2130,0 | 76,1 |
| 2131,0 | 76,1 |
| 2131,9 | 76,2 |
| 2132,9 | 76,2 |
| 2133,8 | 76,3 |
| 2134,8 | 76,4 |
| 2135,8 | 76,4 |
| 2136,7 | 76,5 |
| 2137,7 | 76,6 |
| 2138,7 | 76,6 |
| 2139,6 | 76,7 |
| 2140,6 | 76,8 |
| 2141,6 | 76,8 |
| 2142,5 | 76,9 |
| 2143,5 | 77,0 |
| 2144,5 | 77,1 |
| 2145,4 | 77,1 |
| 2146,4 | 77,2 |
| 2147,3 | 77,3 |
| 2148,3 | 77,4 |
| 2149,3 | 77,5 |
| 2150,2 | 77,5 |
| 2151,2 | 77,6 |
| 2152,2 | 77,7 |
| 2153,1 | 77,8 |
| 2154,1 | 77,9 |
| 2155,1 | 78,0 |
| 2156,0 | 78,0 |
| 2157,0 | 78,1 |
| 2158,0 | 78,2 |
| 2158,9 | 78,3 |
| 2159,9 | 78,4 |
| 2160,8 | 78,5 |
| 2161,8 | 78,6 |
| 2162,8 | 78,7 |
| 2163,7 | 78,7 |
| 2164,7 | 78,8 |
| 2165,7 | 78,9 |
| 2166,6 | 79,0 |
| 2167,6 | 79,1 |
| 2168,6 | 79,2 |
| 2169,5 | 79,3 |
| 2170,5 | 79,4 |
| 2171,5 | 79,5 |
| 2172,4 | 79,6 |
| 2173,4 | 79,7 |
| 2174,3 | 79,8 |
| 2175,3 | 79,9 |
| 2176,3 | 80,0 |
| 2177,2 | 80,1 |
| 2178,2 | 80,2 |
| 2179,2 | 80,3 |
| 2180,1 | 80,4 |
| 2181,1 | 80,5 |
| 2182,1 | 80,6 |
| 2183,0 | 80,7 |
| 2184,0 | 80,8 |
| 2185,0 | 80,9 |
| 2185,9 | 81,0 |
| 2186,9 | 81,1 |
| 2187,8 | 81,2 |
| 2188,8 | 81,3 |
| 2189,8 | 81,4 |
| 2190,7 | 81,5 |
| 2191,7 | 81,6 |
| 2192,7 | 81,7 |
| 2193,6 | 81,8 |
| 2194,6 | 81,9 |
| 2195,6 | 82,0 |
| 2196,5 | 82,1 |
| 2197,5 | 82,2 |
| 2198,5 | 82,4 |
| 2199,4 | 82,5 |
| 2200,4 | 82,6 |
| 2201,3 | 82,7 |
| 2202,3 | 82,8 |
| 2203,3 | 82,9 |
| 2204,2 | 83,0 |
| 2205,2 | 83,1 |
| 2206,2 | 83,2 |
| 2207,1 | 83,3 |
| 2208,1 | 83,5 |
| 2209,1 | 83,6 |
| 2210,0 | 83,7 |
| 2211,0 | 83,8 |
| 2212,0 | 83,9 |
| 2212,9 | 84,0 |
| 2213,9 | 84,1 |
| 2214,8 | 84,3 |
| 2215,8 | 84,4 |
| 2216,8 | 84,5 |
| 2217,7 | 84,6 |
| 2218,7 | 84,7 |
| 2219,7 | 84,8 |
| 2220,6 | 85,0 |
| 2221,6 | 85,1 |
| 2222,6 | 85,2 |
| 2223,5 | 85,3 |
| 2224,5 | 85,4 |
| 2225,5 | 85,5 |
| 2226,4 | 85,6 |
| 2227,4 | 85,7 |
| 2228,3 | 85,9 |
| 2229,3 | 86,0 |
| 2230,3 | 86,1 |
| 2231,2 | 86,2 |
| 2232,2 | 86,3 |
| 2233,2 | 86,4 |
| 2234,1 | 86,6 |
| 2235,1 | 86,7 |
| 2236,1 | 86,8 |
| 2237,0 | 86,9 |
| 2238,0 | 87,0 |
| 2239,0 | 87,1 |
| 2239,9 | 87,3 |
| 2240,9 | 87,4 |
| 2241,8 | 87,5 |
| 2242,8 | 87,6 |
| 2243,8 | 87,7 |
| 2244,7 | 87,8 |
| 2245,7 | 87,9 |
| 2246,7 | 88,0 |
| 2247,6 | 88,2 |
| 2248,6 | 88,3 |
| 2249,6 | 88,4 |
| 2250,5 | 88,5 |
| 2251,5 | 88,6 |
| 2252,4 | 88,7 |
| 2253,4 | 88,8 |
| 2254,4 | 89,0 |
| 2255,3 | 89,1 |
| 2256,3 | 89,2 |
| 2257,3 | 89,3 |
| 2258,2 | 89,4 |
| 2259,2 | 89,5 |
| 2260,2 | 89,6 |
| 2261,1 | 89,7 |
| 2262,1 | 89,8 |
| 2263,1 | 89,9 |
| 2264,0 | 90,0 |
| 2265,0 | 90,2 |
| 2265,9 | 90,3 |
| 2266,9 | 90,4 |
| 2267,9 | 90,5 |
| 2268,8 | 90,6 |
| 2269,8 | 90,7 |
| 2270,8 | 90,8 |
| 2271,7 | 90,9 |
| 2272,7 | 91,0 |
| 2273,7 | 91,1 |
| 2274,6 | 91,2 |
| 2275,6 | 91,3 |
| 2276,6 | 91,4 |
| 2277,5 | 91,5 |
| 2278,5 | 91,6 |
| 2279,4 | 91,7 |
| 2280,4 | 91,8 |
| 2281,4 | 91,9 |
| 2282,3 | 92,0 |
| 2283,3 | 92,1 |
| 2284,3 | 92,2 |
| 2285,2 | 92,3 |
| 2286,2 | 92,4 |
| 2287,2 | 92,5 |
| 2288,1 | 92,6 |
| 2289,1 | 92,7 |
| 2290,1 | 92,8 |
| 2291,0 | 92,9 |
| 2292,0 | 93,0 |
| 2292,9 | 93,1 |
| 2293,9 | 93,2 |
| 2294,9 | 93,3 |
| 2295,8 | 93,4 |
| 2296,8 | 93,4 |
| 2297,8 | 93,5 |
| 2298,7 | 93,6 |
| 2299,7 | 93,7 |
| 2300,7 | 93,8 |
| 2301,6 | 93,9 |
| 2302,6 | 94,0 |
| 2303,6 | 94,1 |
| 2304,5 | 94,2 |
| 2305,5 | 94,3 |
| 2306,4 | 94,4 |
| 2307,4 | 94,5 |
| 2308,4 | 94,6 |
| 2309,3 | 94,7 |
| 2310,3 | 94,8 |
| 2311,3 | 94,9 |
| 2312,2 | 95,0 |
| 2313,2 | 95,1 |
| 2314,2 | 95,2 |
| 2315,1 | 95,3 |
| 2316,1 | 95,4 |
| 2317,1 | 95,5 |
| 2318,0 | 95,5 |
| 2319,0 | 95,6 |
| 2319,9 | 95,7 |
| 2320,9 | 95,8 |
| 2321,9 | 95,9 |
| 2322,8 | 96,0 |
| 2323,8 | 96,0 |
| 2324,8 | 96,1 |
| 2325,7 | 96,2 |
| 2326,7 | 96,3 |
| 2327,7 | 96,4 |
| 2328,6 | 96,5 |
| 2329,6 | 96,5 |
| 2330,6 | 96,6 |
| 2331,5 | 96,7 |
| 2332,5 | 96,7 |
| 2333,4 | 96,8 |
| 2334,4 | 96,8 |
| 2335,4 | 96,9 |
| 2336,3 | 96,9 |
| 2337,3 | 97,0 |
| 2338,3 | 97,1 |
| 2339,2 | 97,2 |
| 2340,2 | 97,3 |
| 2341,2 | 97,4 |
| 2342,1 | 97,5 |
| 2343,1 | 97,5 |
| 2344,1 | 97,6 |
| 2345,0 | 97,7 |
| 2346,0 | 97,8 |
| 2346,9 | 97,9 |
| 2347,9 | 97,9 |
| 2348,9 | 98,0 |
| 2349,8 | 98,1 |
| 2350,8 | 98,1 |
| 2351,8 | 98,2 |
| 2352,7 | 98,2 |
| 2353,7 | 98,3 |
| 2354,7 | 98,3 |
| 2355,6 | 98,4 |
| 2356,6 | 98,5 |
| 2357,6 | 98,5 |
| 2358,5 | 98,6 |
| 2359,5 | 98,7 |
| 2360,4 | 98,7 |
| 2361,4 | 98,7 |
| 2362,4 | 98,8 |
| 2363,3 | 98,8 |
| 2364,3 | 98,8 |
| 2365,3 | 98,8 |
| 2366,2 | 98,8 |
| 2367,2 | 98,8 |
| 2368,2 | 98,8 |
| 2369,1 | 98,8 |
| 2370,1 | 98,8 |
| 2371,1 | 98,9 |
| 2372,0 | 98,9 |
| 2373,0 | 98,9 |
| 2373,9 | 98,9 |
| 2374,9 | 98,9 |
| 2375,9 | 99,0 |
| 2376,8 | 99,0 |
| 2377,8 | 99,0 |
| 2378,8 | 99,0 |
| 2379,7 | 99,1 |
| 2380,7 | 99,1 |
| 2381,7 | 99,1 |
| 2382,6 | 99,1 |
| 2383,6 | 99,1 |
| 2384,5 | 99,2 |
| 2385,5 | 99,2 |
| 2386,5 | 99,2 |
| 2387,4 | 99,2 |
| 2388,4 | 99,2 |
| 2389,4 | 99,2 |
| 2390,3 | 99,3 |
| 2391,3 | 99,3 |
| 2392,3 | 99,3 |
| 2393,2 | 99,3 |
| 2394,2 | 99,3 |
| 2395,2 | 99,3 |
| 2396,1 | 99,3 |
| 2397,1 | 99,3 |
| 2398,0 | 99,3 |
| 2399,0 | 99,3 |
| 2400,0 | 99,2 |
| 2400,9 | 99,2 |
| 2401,9 | 99,2 |
| 2402,9 | 99,2 |
| 2403,8 | 99,2 |
| 2404,8 | 99,2 |
| 2405,8 | 99,2 |
| 2406,7 | 99,2 |
| 2407,7 | 99,2 |
| 2408,7 | 99,2 |
| 2409,6 | 99,2 |
| 2410,6 | 99,2 |
| 2411,5 | 99,2 |
| 2412,5 | 99,2 |
| 2413,5 | 99,2 |
| 2414,4 | 99,2 |
| 2415,4 | 99,2 |
| 2416,4 | 99,2 |
| 2417,3 | 99,2 |
| 2418,3 | 99,2 |
| 2419,3 | 99,2 |
| 2420,2 | 99,2 |
| 2421,2 | 99,2 |
| 2422,2 | 99,2 |
| 2423,1 | 99,2 |
| 2424,1 | 99,2 |
| 2425,0 | 99,2 |
| 2426,0 | 99,2 |
| 2427,0 | 99,2 |
| 2427,9 | 99,2 |
| 2428,9 | 99,2 |
| 2429,9 | 99,2 |
| 2430,8 | 99,2 |
| 2431,8 | 99,2 |
| 2432,8 | 99,3 |
| 2433,7 | 99,3 |
| 2434,7 | 99,3 |
| 2435,7 | 99,3 |
| 2436,6 | 99,3 |
| 2437,6 | 99,3 |
| 2438,5 | 99,3 |
| 2439,5 | 99,3 |
| 2440,5 | 99,3 |
| 2441,4 | 99,2 |
| 2442,4 | 99,2 |
| 2443,4 | 99,2 |
| 2444,3 | 99,2 |
| 2445,3 | 99,2 |
| 2446,3 | 99,2 |
| 2447,2 | 99,2 |
| 2448,2 | 99,2 |
| 2449,2 | 99,2 |
| 2450,1 | 99,2 |
| 2451,1 | 99,2 |
| 2452,0 | 99,2 |
| 2453,0 | 99,2 |
| 2454,0 | 99,2 |
| 2454,9 | 99,2 |
| 2455,9 | 99,2 |
| 2456,9 | 99,2 |
| 2457,8 | 99,2 |
| 2458,8 | 99,2 |
| 2459,8 | 99,2 |
| 2460,7 | 99,2 |
| 2461,7 | 99,2 |
| 2462,7 | 99,2 |
| 2463,6 | 99,2 |
| 2464,6 | 99,2 |
| 2465,5 | 99,2 |
| 2466,5 | 99,2 |
| 2467,5 | 99,2 |
| 2468,4 | 99,2 |
| 2469,4 | 99,2 |
| 2470,4 | 99,2 |
| 2471,3 | 99,2 |
| 2472,3 | 99,2 |
| 2473,3 | 99,2 |
| 2474,2 | 99,2 |
| 2475,2 | 99,2 |
| 2476,2 | 99,2 |
| 2477,1 | 99,2 |
| 2478,1 | 99,2 |
| 2479,0 | 99,2 |
| 2480,0 | 99,2 |
| 2481,0 | 99,2 |
| 2481,9 | 99,2 |
| 2482,9 | 99,2 |
| 2483,9 | 99,2 |
| 2484,8 | 99,2 |
| 2485,8 | 99,2 |
| 2486,8 | 99,2 |
| 2487,7 | 99,2 |
| 2488,7 | 99,2 |
| 2489,7 | 99,2 |
| 2490,6 | 99,3 |
| 2491,6 | 99,3 |
| 2492,5 | 99,3 |
| 2493,5 | 99,3 |
| 2494,5 | 99,3 |
| 2495,4 | 99,3 |
| 2496,4 | 99,3 |
| 2497,4 | 99,3 |
| 2498,3 | 99,3 |
| 2499,3 | 99,4 |
| 2500,3 | 99,4 |
| 2501,2 | 99,4 |
| 2502,2 | 99,4 |
| 2503,2 | 99,4 |
| 2504,1 | 99,4 |
| 2505,1 | 99,5 |
| 2506,0 | 99,5 |
| 2507,0 | 99,5 |
| 2508,0 | 99,5 |
| 2508,9 | 99,5 |
| 2509,9 | 99,5 |
| 2510,9 | 99,5 |
| 2511,8 | 99,5 |
| 2512,8 | 99,5 |
| 2513,8 | 99,5 |
| 2514,7 | 99,5 |
| 2515,7 | 99,4 |
| 2516,6 | 99,4 |
| 2517,6 | 99,4 |
| 2518,6 | 99,4 |
| 2519,5 | 99,4 |
| 2520,5 | 99,4 |
| 2521,5 | 99,3 |
| 2522,4 | 99,3 |
| 2523,4 | 99,3 |
| 2524,4 | 99,3 |
| 2525,3 | 99,2 |
| 2526,3 | 99,2 |
| 2527,3 | 99,2 |
| 2528,2 | 99,1 |
| 2529,2 | 99,1 |
| 2530,1 | 99,1 |
| 2531,1 | 99,0 |
| 2532,1 | 99,0 |
| 2533,0 | 98,9 |
| 2534,0 | 98,9 |
| 2535,0 | 98,8 |
| 2535,9 | 98,8 |
| 2536,9 | 98,7 |
| 2537,9 | 98,7 |
| 2538,8 | 98,6 |
| 2539,8 | 98,6 |
| 2540,8 | 98,5 |
| 2541,7 | 98,5 |
| 2542,7 | 98,4 |
| 2543,6 | 98,3 |
| 2544,6 | 98,3 |
| 2545,6 | 98,2 |
| 2546,5 | 98,2 |
| 2547,5 | 98,1 |
| 2548,5 | 98,0 |
| 2549,4 | 98,0 |
| 2550,4 | 97,9 |
| 2551,4 | 97,8 |
| 2552,3 | 97,7 |
| 2553,3 | 97,7 |
| 2554,3 | 97,6 |
| 2555,2 | 97,5 |
| 2556,2 | 97,4 |
| 2557,1 | 97,4 |
| 2558,1 | 97,3 |
| 2559,1 | 97,2 |
| 2560,0 | 97,1 |
| 2561,0 | 97,0 |
| 2562,0 | 96,9 |
| 2562,9 | 96,9 |
| 2563,9 | 96,8 |
| 2564,9 | 96,7 |
| 2565,8 | 96,6 |
| 2566,8 | 96,5 |
| 2567,8 | 96,4 |
| 2568,7 | 96,3 |
| 2569,7 | 96,2 |
| 2570,6 | 96,1 |
| 2571,6 | 96,0 |
| 2572,6 | 95,9 |
| 2573,5 | 95,8 |
| 2574,5 | 95,7 |
| 2575,5 | 95,6 |
| 2576,4 | 95,5 |
| 2577,4 | 95,4 |
| 2578,4 | 95,3 |
| 2579,3 | 95,2 |
| 2580,3 | 95,1 |
| 2581,3 | 95,0 |
| 2582,2 | 94,9 |
| 2583,2 | 94,8 |
| 2584,1 | 94,6 |
| 2585,1 | 94,5 |
| 2586,1 | 94,4 |
| 2587,0 | 94,3 |
| 2588,0 | 94,2 |
| 2589,0 | 94,1 |
| 2589,9 | 94,0 |
| 2590,9 | 93,8 |
| 2591,9 | 93,7 |
| 2592,8 | 93,6 |
| 2593,8 | 93,5 |
| 2594,8 | 93,3 |
| 2595,7 | 93,2 |
| 2596,7 | 93,1 |
| 2597,6 | 93,0 |
| 2598,6 | 92,8 |
| 2599,6 | 92,7 |
| 2600,5 | 92,6 |
| 2601,5 | 92,4 |
| 2602,5 | 92,3 |
| 2603,4 | 92,2 |
| 2604,4 | 92,0 |
| 2605,4 | 91,9 |
| 2606,3 | 91,8 |
| 2607,3 | 91,6 |
| 2608,3 | 91,5 |
| 2609,2 | 91,4 |
| 2610,2 | 91,2 |
| 2611,1 | 91,1 |
| 2612,1 | 90,9 |
| 2613,1 | 90,8 |
| 2614,0 | 90,7 |
| 2615,0 | 90,5 |
| 2616,0 | 90,4 |
| 2616,9 | 90,2 |
| 2617,9 | 90,1 |
| 2618,9 | 89,9 |
| 2619,8 | 89,8 |
| 2620,8 | 89,6 |
| 2621,8 | 89,5 |
| 2622,7 | 89,3 |
| 2623,7 | 89,2 |
| 2624,6 | 89,0 |
| 2625,6 | 88,8 |
| 2626,6 | 88,7 |
| 2627,5 | 88,5 |
| 2628,5 | 88,4 |
| 2629,5 | 88,2 |
| 2630,4 | 88,0 |
| 2631,4 | 87,9 |
| 2632,4 | 87,7 |
| 2633,3 | 87,6 |
| 2634,3 | 87,4 |
| 2635,3 | 87,2 |
| 2636,2 | 87,0 |
| 2637,2 | 86,9 |
| 2638,1 | 86,7 |
| 2639,1 | 86,5 |
| 2640,1 | 86,4 |
| 2641,0 | 86,2 |
| 2642,0 | 86,0 |
| 2643,0 | 85,8 |
| 2643,9 | 85,7 |
| 2644,9 | 85,5 |
| 2645,9 | 85,3 |
| 2646,8 | 85,1 |
| 2647,8 | 84,9 |
| 2648,7 | 84,8 |
| 2649,7 | 84,6 |
| 2650,7 | 84,4 |
| 2651,6 | 84,2 |
| 2652,6 | 84,0 |
| 2653,6 | 83,8 |
| 2654,5 | 83,7 |
| 2655,5 | 83,5 |
| 2656,5 | 83,3 |
| 2657,4 | 83,1 |
| 2658,4 | 82,9 |
| 2659,4 | 82,7 |
| 2660,3 | 82,5 |
| 2661,3 | 82,3 |
| 2662,2 | 82,1 |
| 2663,2 | 81,9 |
| 2664,2 | 81,8 |
| 2665,1 | 81,6 |
| 2666,1 | 81,4 |
| 2667,1 | 81,2 |
| 2668,0 | 81,0 |
| 2669,0 | 80,8 |
| 2670,0 | 80,6 |
| 2670,9 | 80,4 |
| 2671,9 | 80,2 |
| 2672,9 | 80,0 |
| 2673,8 | 79,8 |
| 2674,8 | 79,5 |
| 2675,7 | 79,3 |
| 2676,7 | 79,1 |
| 2677,7 | 78,9 |
| 2678,6 | 78,7 |
| 2679,6 | 78,5 |
| 2680,6 | 78,3 |
| 2681,5 | 78,1 |
| 2682,5 | 77,9 |
| 2683,5 | 77,7 |
| 2684,4 | 77,4 |
| 2685,4 | 77,2 |
| 2686,4 | 77,0 |
| 2687,3 | 76,8 |
| 2688,3 | 76,6 |
| 2689,2 | 76,4 |
| 2690,2 | 76,1 |
| 2691,2 | 75,9 |
| 2692,1 | 75,7 |
| 2693,1 | 75,5 |
| 2694,1 | 75,3 |
| 2695,0 | 75,0 |
| 2696,0 | 74,8 |
| 2697,0 | 74,6 |
| 2697,9 | 74,4 |
| 2698,9 | 74,2 |
| 2699,9 | 73,9 |
| 2700,8 | 73,7 |
| 2701,8 | 73,5 |
| 2702,7 | 73,2 |
| 2703,7 | 73,0 |
| 2704,7 | 72,8 |
| 2705,6 | 72,6 |
| 2706,6 | 72,3 |
| 2707,6 | 72,1 |
| 2708,5 | 71,9 |
| 2709,5 | 71,7 |
| 2710,5 | 71,4 |
| 2711,4 | 71,2 |
| 2712,4 | 71,0 |
| 2713,4 | 70,7 |
| 2714,3 | 70,5 |
| 2715,3 | 70,3 |
| 2716,2 | 70,0 |
| 2717,2 | 69,8 |
| 2718,2 | 69,6 |
| 2719,1 | 69,3 |
| 2720,1 | 69,1 |
| 2721,1 | 68,9 |
| 2722,0 | 68,6 |
| 2723,0 | 68,4 |
| 2724,0 | 68,1 |
| 2724,9 | 67,9 |
| 2725,9 | 67,7 |
| 2726,9 | 67,4 |
| 2727,8 | 67,2 |
| 2728,8 | 67,0 |
| 2729,7 | 66,7 |
| 2730,7 | 66,5 |
| 2731,7 | 66,2 |
| 2732,6 | 66,0 |
| 2733,6 | 65,8 |
| 2734,6 | 65,5 |
| 2735,5 | 65,3 |
| 2736,5 | 65,0 |
| 2737,5 | 64,8 |
| 2738,4 | 64,5 |
| 2739,4 | 64,3 |
| 2740,4 | 64,1 |
| 2741,3 | 63,8 |
| 2742,3 | 63,6 |
| 2743,2 | 63,3 |
| 2744,2 | 63,1 |
| 2745,2 | 62,8 |
| 2746,1 | 62,6 |
| 2747,1 | 62,3 |
| 2748,1 | 62,1 |
| 2749,0 | 61,8 |
| 2750,0 | 61,6 |
| 2751,0 | 61,3 |
| 2751,9 | 61,1 |
| 2752,9 | 60,8 |
| 2753,9 | 60,6 |
| 2754,8 | 60,3 |
| 2755,8 | 60,1 |
| 2756,7 | 59,9 |
| 2757,7 | 59,6 |
| 2758,7 | 59,4 |
| 2759,6 | 59,1 |
| 2760,6 | 58,9 |
| 2761,6 | 58,6 |
| 2762,5 | 58,4 |
| 2763,5 | 58,1 |
| 2764,5 | 57,9 |
| 2765,4 | 57,6 |
| 2766,4 | 57,4 |
| 2767,4 | 57,1 |
| 2768,3 | 56,9 |
| 2769,3 | 56,6 |
| 2770,2 | 56,4 |
| 2771,2 | 56,1 |
| 2772,2 | 55,9 |
| 2773,1 | 55,6 |
| 2774,1 | 55,4 |
| 2775,1 | 55,2 |
| 2776,0 | 54,9 |
| 2777,0 | 54,7 |
| 2778,0 | 54,4 |
| 2778,9 | 54,2 |
| 2779,9 | 54,0 |
| 2780,8 | 53,7 |
| 2781,8 | 53,5 |
| 2782,8 | 53,2 |
| 2783,7 | 53,0 |
| 2784,7 | 52,8 |
| 2785,7 | 52,5 |
| 2786,6 | 52,3 |
| 2787,6 | 52,1 |
| 2788,6 | 51,8 |
| 2789,5 | 51,6 |
| 2790,5 | 51,3 |
| 2791,5 | 51,1 |
| 2792,4 | 50,9 |
| 2793,4 | 50,7 |
| 2794,3 | 50,4 |
| 2795,3 | 50,2 |
| 2796,3 | 50,0 |
| 2797,2 | 49,7 |
| 2798,2 | 49,5 |
| 2799,2 | 49,3 |
| 2800,1 | 49,1 |
| 2801,1 | 48,8 |
| 2802,1 | 48,6 |
| 2803,0 | 48,4 |
| 2804,0 | 48,2 |
| 2805,0 | 47,9 |
| 2805,9 | 47,7 |
| 2806,9 | 47,5 |
| 2807,8 | 47,3 |
| 2808,8 | 47,1 |
| 2809,8 | 46,8 |
| 2810,7 | 46,6 |
| 2811,7 | 46,4 |
| 2812,7 | 46,2 |
| 2813,6 | 46,0 |
| 2814,6 | 45,7 |
| 2815,6 | 45,5 |
| 2816,5 | 45,3 |
| 2817,5 | 45,1 |
| 2818,5 | 44,9 |
| 2819,4 | 44,7 |
| 2820,4 | 44,4 |
| 2821,3 | 44,2 |
| 2822,3 | 44,0 |
| 2823,3 | 43,8 |
| 2824,2 | 43,6 |
| 2825,2 | 43,4 |
| 2826,2 | 43,2 |
| 2827,1 | 43,0 |
| 2828,1 | 42,7 |
| 2829,1 | 42,5 |
| 2830,0 | 42,3 |
| 2831,0 | 42,1 |
| 2832,0 | 41,9 |
| 2832,9 | 41,7 |
| 2833,9 | 41,5 |
| 2834,8 | 41,3 |
| 2835,8 | 41,0 |
| 2836,8 | 40,8 |
| 2837,7 | 40,6 |
| 2838,7 | 40,4 |
| 2839,7 | 40,2 |
| 2840,6 | 40,0 |
| 2841,6 | 39,7 |
| 2842,6 | 39,5 |
| 2843,5 | 39,3 |
| 2844,5 | 39,1 |
| 2845,5 | 38,9 |
| 2846,4 | 38,7 |
| 2847,4 | 38,5 |
| 2848,3 | 38,3 |
| 2849,3 | 38,1 |
| 2850,3 | 37,9 |
| 2851,2 | 37,7 |
| 2852,2 | 37,5 |
| 2853,2 | 37,3 |
| 2854,1 | 37,1 |
| 2855,1 | 36,9 |
| 2856,1 | 36,7 |
| 2857,0 | 36,5 |
| 2858,0 | 36,3 |
| 2859,0 | 36,1 |
| 2859,9 | 35,9 |
| 2860,9 | 35,7 |
| 2861,8 | 35,6 |
| 2862,8 | 35,4 |
| 2863,8 | 35,2 |
| 2864,7 | 35,0 |
| 2865,7 | 34,8 |
| 2866,7 | 34,7 |
| 2867,6 | 34,5 |
| 2868,6 | 34,3 |
| 2869,6 | 34,1 |
| 2870,5 | 34,0 |
| 2871,5 | 33,8 |
| 2872,5 | 33,6 |
| 2873,4 | 33,4 |
| 2874,4 | 33,3 |
| 2875,3 | 33,1 |
| 2876,3 | 32,9 |
| 2877,3 | 32,7 |
| 2878,2 | 32,5 |
| 2879,2 | 32,4 |
| 2880,2 | 32,2 |
| 2881,1 | 32,0 |
| 2882,1 | 31,8 |
| 2883,1 | 31,7 |
| 2884,0 | 31,5 |
| 2885,0 | 31,3 |
| 2886,0 | 31,1 |
| 2886,9 | 31,0 |
| 2887,9 | 30,8 |
| 2888,8 | 30,6 |
| 2889,8 | 30,5 |
| 2890,8 | 30,3 |
| 2891,7 | 30,1 |
| 2892,7 | 29,9 |
| 2893,7 | 29,8 |
| 2894,6 | 29,6 |
| 2895,6 | 29,4 |
| 2896,6 | 29,3 |
| 2897,5 | 29,1 |
| 2898,5 | 28,9 |
| 2899,5 | 28,7 |
| 2900,4 | 28,6 |
| 2901,4 | 28,4 |
| 2902,3 | 28,2 |
| 2903,3 | 28,1 |
| 2904,3 | 27,9 |
| 2905,2 | 27,7 |
| 2906,2 | 27,6 |
| 2907,2 | 27,4 |
| 2908,1 | 27,2 |
| 2909,1 | 27,1 |
| 2910,1 | 26,9 |
| 2911,0 | 26,7 |
| 2912,0 | 26,6 |
| 2912,9 | 26,4 |
| 2913,9 | 26,2 |
| 2914,9 | 26,1 |
| 2915,8 | 25,9 |
| 2916,8 | 25,8 |
| 2917,8 | 25,6 |
| 2918,7 | 25,5 |
| 2919,7 | 25,3 |
| 2920,7 | 25,1 |
| 2921,6 | 25,0 |
| 2922,6 | 24,8 |
| 2923,6 | 24,7 |
| 2924,5 | 24,5 |
| 2925,5 | 24,4 |
| 2926,4 | 24,2 |
| 2927,4 | 24,1 |
| 2928,4 | 24,0 |
| 2929,3 | 23,8 |
| 2930,3 | 23,7 |
| 2931,3 | 23,6 |
| 2932,2 | 23,4 |
| 2933,2 | 23,3 |
| 2934,2 | 23,1 |
| 2935,1 | 23,0 |
| 2936,1 | 22,9 |
| 2937,1 | 22,8 |
| 2938,0 | 22,6 |
| 2939,0 | 22,5 |
| 2939,9 | 22,4 |
| 2940,9 | 22,2 |
| 2941,9 | 22,1 |
| 2942,8 | 22,0 |
| 2943,8 | 21,8 |
| 2944,8 | 21,7 |
| 2945,7 | 21,6 |
| 2946,7 | 21,5 |
| 2947,7 | 21,3 |
| 2948,6 | 21,2 |
| 2949,6 | 21,1 |
| 2950,6 | 20,9 |
| 2951,5 | 20,8 |
| 2952,5 | 20,7 |
| 2953,4 | 20,5 |
| 2954,4 | 20,4 |
| 2955,4 | 20,3 |
| 2956,3 | 20,2 |
| 2957,3 | 20,0 |
| 2958,3 | 19,9 |
| 2959,2 | 19,8 |
| 2960,2 | 19,7 |
| 2961,2 | 19,5 |
| 2962,1 | 19,4 |
| 2963,1 | 19,3 |
| 2964,1 | 19,2 |
| 2965,0 | 19,1 |
| 2966,0 | 18,9 |
| 2966,9 | 18,8 |
| 2967,9 | 18,7 |
| 2968,9 | 18,6 |
| 2969,8 | 18,4 |
| 2970,8 | 18,3 |
| 2971,8 | 18,2 |
| 2972,7 | 18,1 |
| 2973,7 | 18,0 |
| 2974,7 | 17,9 |
| 2975,6 | 17,7 |
| 2976,6 | 17,6 |
| 2977,6 | 17,5 |
| 2978,5 | 17,4 |
| 2979,5 | 17,3 |
| 2980,4 | 17,2 |
| 2981,4 | 17,1 |
| 2982,4 | 17,0 |
| 2983,3 | 16,8 |
| 2984,3 | 16,7 |
| 2985,3 | 16,6 |
| 2986,2 | 16,5 |
| 2987,2 | 16,4 |
| 2988,2 | 16,3 |
| 2989,1 | 16,2 |
| 2990,1 | 16,1 |
| 2991,1 | 16,0 |
| 2992,0 | 15,8 |
| 2993,0 | 15,7 |
| 2993,9 | 15,6 |
| 2994,9 | 15,5 |
| 2995,9 | 15,4 |
| 2996,8 | 15,3 |
| 2997,8 | 15,2 |
| 2998,8 | 15,1 |
| 2999,7 | 15,0 |
| 3000,7 | 14,9 |
| 3001,7 | 14,8 |
| 3002,6 | 14,7 |
| 3003,6 | 14,6 |
| 3004,6 | 14,5 |
| 3005,5 | 14,4 |
| 3006,5 | 14,3 |
| 3007,4 | 14,2 |
| 3008,4 | 14,1 |
| 3009,4 | 14,0 |
| 3010,3 | 13,9 |
| 3011,3 | 13,8 |
| 3012,3 | 13,7 |
| 3013,2 | 13,6 |
| 3014,2 | 13,5 |
| 3015,2 | 13,4 |
| 3016,1 | 13,3 |
| 3017,1 | 13,2 |
| 3018,1 | 13,1 |
| 3019,0 | 13,0 |
| 3020,0 | 12,9 |
| 3020,9 | 12,8 |
| 3021,9 | 12,7 |
| 3022,9 | 12,6 |
| 3023,8 | 12,5 |
| 3024,8 | 12,4 |
| 3025,8 | 12,3 |
| 3026,7 | 12,2 |
| 3027,7 | 12,1 |
| 3028,7 | 12,1 |
| 3029,6 | 12,0 |
| 3030,6 | 11,9 |
| 3031,6 | 11,8 |
| 3032,5 | 11,7 |
| 3033,5 | 11,6 |
| 3034,4 | 11,5 |
| 3035,4 | 11,5 |
| 3036,4 | 11,4 |
| 3037,3 | 11,3 |
| 3038,3 | 11,2 |
| 3039,3 | 11,1 |
| 3040,2 | 11,0 |
| 3041,2 | 11,0 |
| 3042,2 | 10,9 |
| 3043,1 | 10,8 |
| 3044,1 | 10,7 |
| 3045,0 | 10,6 |
| 3046,0 | 10,6 |
| 3047,0 | 10,5 |
| 3047,9 | 10,4 |
| 3048,9 | 10,3 |
| 3049,9 | 10,3 |
| 3050,8 | 10,2 |
| 3051,8 | 10,1 |
| 3052,8 | 10,0 |
| 3053,7 | 9,9 |
| 3054,7 | 9,9 |
| 3055,7 | 9,8 |
| 3056,6 | 9,7 |
| 3057,6 | 9,7 |
| 3058,5 | 9,6 |
| 3059,5 | 9,5 |
| 3060,5 | 9,4 |
| 3061,4 | 9,4 |
| 3062,4 | 9,3 |
| 3063,4 | 9,2 |
| 3064,3 | 9,2 |
| 3065,3 | 9,1 |
| 3066,3 | 9,0 |
| 3067,2 | 9,0 |
| 3068,2 | 8,9 |
| 3069,2 | 8,9 |
| 3070,1 | 8,8 |
| 3071,1 | 8,7 |
| 3072,0 | 8,7 |
| 3073,0 | 8,6 |
| 3074,0 | 8,6 |
| 3074,9 | 8,5 |
| 3075,9 | 8,4 |
| 3076,9 | 8,4 |
| 3077,8 | 8,3 |
| 3078,8 | 8,3 |
| 3079,8 | 8,2 |
| 3080,7 | 8,2 |
| 3081,7 | 8,1 |
| 3082,7 | 8,1 |
| 3083,6 | 8,0 |
| 3084,6 | 8,0 |
| 3085,5 | 7,9 |
| 3086,5 | 7,9 |
| 3087,5 | 7,8 |
| 3088,4 | 7,8 |
| 3089,4 | 7,7 |
| 3090,4 | 7,7 |
| 3091,3 | 7,6 |
| 3092,3 | 7,6 |
| 3093,3 | 7,5 |
| 3094,2 | 7,5 |
| 3095,2 | 7,5 |
| 3096,2 | 7,4 |
| 3097,1 | 7,4 |
| 3098,1 | 7,3 |
| 3099,0 | 7,3 |
| 3100,0 | 7,2 |
| 3101,0 | 7,2 |
| 3101,9 | 7,2 |
| 3102,9 | 7,1 |
| 3103,9 | 7,1 |
| 3104,8 | 7,0 |
| 3105,8 | 7,0 |
| 3106,8 | 7,0 |
| 3107,7 | 6,9 |
| 3108,7 | 6,9 |
| 3109,7 | 6,8 |
| 3110,6 | 6,8 |
| 3111,6 | 6,8 |
| 3112,5 | 6,7 |
| 3113,5 | 6,7 |
| 3114,5 | 6,7 |
| 3115,4 | 6,6 |
| 3116,4 | 6,6 |
| 3117,4 | 6,5 |
| 3118,3 | 6,5 |
| 3119,3 | 6,5 |
| 3120,3 | 6,4 |
| 3121,2 | 6,4 |
| 3122,2 | 6,4 |
| 3123,2 | 6,3 |
| 3124,1 | 6,3 |
| 3125,1 | 6,2 |
| 3126,0 | 6,2 |
| 3127,0 | 6,2 |
| 3128,0 | 6,1 |
| 3128,9 | 6,1 |
| 3129,9 | 6,1 |
| 3130,9 | 6,0 |
| 3131,8 | 6,0 |
| 3132,8 | 6,0 |
| 3133,8 | 5,9 |
| 3134,7 | 5,9 |
| 3135,7 | 5,8 |
| 3136,7 | 5,8 |
| 3137,6 | 5,8 |
| 3138,6 | 5,7 |
| 3139,5 | 5,7 |
| 3140,5 | 5,7 |
| 3141,5 | 5,6 |
| 3142,4 | 5,6 |
| 3143,4 | 5,5 |
| 3144,4 | 5,5 |
| 3145,3 | 5,5 |
| 3146,3 | 5,4 |
| 3147,3 | 5,4 |
| 3148,2 | 5,4 |
| 3149,2 | 5,3 |
| 3150,2 | 5,3 |
| 3151,1 | 5,2 |
| 3152,1 | 5,2 |
| 3153,0 | 5,2 |
| 3154,0 | 5,1 |
| 3155,0 | 5,1 |
| 3155,9 | 5,0 |
| 3156,9 | 5,0 |
| 3157,9 | 5,0 |
| 3158,8 | 4,9 |
| 3159,8 | 4,9 |
| 3160,8 | 4,8 |
| 3161,7 | 4,8 |
| 3162,7 | 4,8 |
| 3163,6 | 4,7 |
| 3164,6 | 4,7 |
| 3165,6 | 4,7 |
| 3166,5 | 4,6 |
| 3167,5 | 4,6 |
| 3168,5 | 4,5 |
| 3169,4 | 4,5 |
| 3170,4 | 4,5 |
| 3171,4 | 4,4 |
| 3172,3 | 4,4 |
| 3173,3 | 4,3 |
| 3174,3 | 4,3 |
| 3175,2 | 4,3 |
| 3176,2 | 4,2 |
| 3177,1 | 4,2 |
| 3178,1 | 4,2 |
| 3179,1 | 4,1 |
| 3180,0 | 4,1 |
| 3181,0 | 4,0 |
| 3182,0 | 4,0 |
| 3182,9 | 4,0 |
| 3183,9 | 3,9 |
| 3184,9 | 3,9 |
| 3185,8 | 3,9 |
| 3186,8 | 3,8 |
| 3187,8 | 3,8 |
| 3188,7 | 3,7 |
| 3189,7 | 3,7 |
| 3190,6 | 3,7 |
| 3191,6 | 3,6 |
| 3192,6 | 3,6 |
| 3193,5 | 3,6 |
| 3194,5 | 3,5 |
| 3195,5 | 3,5 |
| 3196,4 | 3,5 |
| 3197,4 | 3,4 |
| 3198,4 | 3,4 |
| 3199,3 | 3,4 |
| 3200,3 | 3,3 |
| 3201,3 | 3,3 |
| 3202,2 | 3,3 |
| 3203,2 | 3,2 |
| 3204,1 | 3,2 |
| 3205,1 | 3,2 |
| 3206,1 | 3,1 |
| 3207,0 | 3,1 |
| 3208,0 | 3,1 |
| 3209,0 | 3,0 |
| 3209,9 | 3,0 |
| 3210,9 | 3,0 |
| 3211,9 | 2,9 |
| 3212,8 | 2,9 |
| 3213,8 | 2,9 |
| 3214,8 | 2,8 |
| 3215,7 | 2,8 |
| 3216,7 | 2,8 |
| 3217,6 | 2,7 |
| 3218,6 | 2,7 |
| 3219,6 | 2,7 |
| 3220,5 | 2,6 |
| 3221,5 | 2,6 |
| 3222,5 | 2,6 |
| 3223,4 | 2,6 |
| 3224,4 | 2,5 |
| 3225,4 | 2,5 |
| 3226,3 | 2,5 |
| 3227,3 | 2,5 |
| 3228,3 | 2,4 |
| 3229,2 | 2,4 |
| 3230,2 | 2,4 |
| 3231,1 | 2,4 |
| 3232,1 | 2,3 |
| 3233,1 | 2,3 |
| 3234,0 | 2,3 |
| 3235,0 | 2,2 |
| 3236,0 | 2,2 |
| 3236,9 | 2,2 |
| 3237,9 | 2,2 |
| 3238,9 | 2,2 |
| 3239,8 | 2,1 |
| 3240,8 | 2,1 |
| 3241,8 | 2,1 |
| 3242,7 | 2,1 |
| 3243,7 | 2,0 |
| 3244,6 | 2,0 |
| 3245,6 | 2,0 |
| 3246,6 | 2,0 |
| 3247,5 | 2,0 |
| 3248,5 | 1,9 |
| 3249,5 | 1,9 |
| 3250,4 | 1,9 |
| 3251,4 | 1,9 |
| 3252,4 | 1,8 |
| 3253,3 | 1,8 |
| 3254,3 | 1,8 |
| 3255,3 | 1,8 |
| 3256,2 | 1,8 |
| 3257,2 | 1,7 |
| 3258,1 | 1,7 |
| 3259,1 | 1,7 |
| 3260,1 | 1,7 |
| 3261,0 | 1,7 |
| 3262,0 | 1,6 |
| 3263,0 | 1,6 |
| 3263,9 | 1,6 |
| 3264,9 | 1,6 |
| 3265,9 | 1,6 |
| 3266,8 | 1,5 |
| 3267,8 | 1,5 |
| 3268,8 | 1,5 |
| 3269,7 | 1,5 |
| 3270,7 | 1,5 |
| 3271,6 | 1,4 |
| 3272,6 | 1,4 |
| 3273,6 | 1,4 |
| 3274,5 | 1,4 |
| 3275,5 | 1,4 |
| 3276,5 | 1,4 |
| 3277,4 | 1,3 |
| 3278,4 | 1,3 |
| 3279,4 | 1,3 |
| 3280,3 | 1,3 |
| 3281,3 | 1,3 |
| 3282,3 | 1,3 |
| 3283,2 | 1,2 |
| 3284,2 | 1,2 |
| 3285,1 | 1,2 |
| 3286,1 | 1,2 |
| 3287,1 | 1,2 |
| 3288,0 | 1,2 |
| 3289,0 | 1,2 |
| 3290,0 | 1,1 |
| 3290,9 | 1,1 |
| 3291,9 | 1,1 |
| 3292,9 | 1,1 |
| 3293,8 | 1,1 |
| 3294,8 | 1,1 |
| 3295,8 | 1,1 |
| 3296,7 | 1,0 |
| 3297,7 | 1,0 |
| 3298,6 | 1,0 |
| 3299,6 | 1,0 |
| 3300,6 | 1,0 |
| 3301,5 | 1,0 |
| 3302,5 | 1,0 |
| 3303,5 | 1,0 |
| 3304,4 | 1,0 |
| 3305,4 | 0,9 |
| 3306,4 | 0,9 |
| 3307,3 | 0,9 |
| 3308,3 | 0,9 |
| 3309,2 | 0,9 |
| 3310,2 | 0,9 |
| 3311,2 | 0,9 |
| 3312,1 | 0,9 |
| 3313,1 | 0,8 |
| 3314,1 | 0,8 |
| 3315,0 | 0,8 |
| 3316,0 | 0,8 |
| 3317,0 | 0,8 |
| 3317,9 | 0,8 |
| 3318,9 | 0,8 |
| 3319,9 | 0,8 |
| 3320,8 | 0,7 |
| 3321,8 | 0,7 |
| 3322,7 | 0,7 |
| 3323,7 | 0,7 |
| 3324,7 | 0,7 |
| 3325,6 | 0,7 |
| 3326,6 | 0,7 |
| 3327,6 | 0,7 |
| 3328,5 | 0,7 |
| 3329,5 | 0,7 |
| 3330,5 | 0,7 |
| 3331,4 | 0,6 |
| 3332,4 | 0,6 |
| 3333,4 | 0,6 |
| 3334,3 | 0,6 |
| 3335,3 | 0,6 |
| 3336,2 | 0,6 |
| 3337,2 | 0,6 |
| 3338,2 | 0,6 |
| 3339,1 | 0,6 |
| 3340,1 | 0,6 |
| 3341,1 | 0,6 |
| 3342,0 | 0,6 |
| 3343,0 | 0,5 |
| 3344,0 | 0,5 |
| 3344,9 | 0,5 |
| 3345,9 | 0,5 |
| 3346,9 | 0,5 |
| 3347,8 | 0,5 |
| 3348,8 | 0,5 |
| 3349,7 | 0,5 |
| 3350,7 | 0,5 |
| 3351,7 | 0,5 |
| 3352,6 | 0,5 |
| 3353,6 | 0,5 |
| 3354,6 | 0,5 |
| 3355,5 | 0,5 |
| 3356,5 | 0,5 |
| 3357,5 | 0,5 |
| 3358,4 | 0,5 |
| 3359,4 | 0,4 |
| 3360,4 | 0,4 |
| 3361,3 | 0,4 |
| 3362,3 | 0,4 |
| 3363,2 | 0,4 |
| 3364,2 | 0,4 |
| 3365,2 | 0,4 |
| 3366,1 | 0,4 |
| 3367,1 | 0,4 |
| 3368,1 | 0,4 |
| 3369,0 | 0,4 |
| 3370,0 | 0,4 |
| 3371,0 | 0,4 |
| 3371,9 | 0,4 |
| 3372,9 | 0,4 |
| 3373,9 | 0,4 |
| 3374,8 | 0,4 |
| 3375,8 | 0,4 |
| 3376,7 | 0,4 |
| 3377,7 | 0,4 |
| 3378,7 | 0,4 |
| 3379,6 | 0,4 |
| 3380,6 | 0,3 |
| 3381,6 | 0,3 |
| 3382,5 | 0,3 |
| 3383,5 | 0,3 |
| 3384,5 | 0,3 |
| 3385,4 | 0,3 |
| 3386,4 | 0,3 |
| 3387,4 | 0,3 |
| 3388,3 | 0,3 |
| 3389,3 | 0,3 |
| 3390,2 | 0,3 |
| 3391,2 | 0,3 |
| 3392,2 | 0,3 |
| 3393,1 | 0,3 |
| 3394,1 | 0,3 |
| 3395,1 | 0,3 |
| 3396,0 | 0,3 |
| 3397,0 | 0,3 |
| 3398,0 | 0,3 |
| 3398,9 | 0,3 |
| 3399,9 | 0,3 |
| 3400,9 | 0,3 |
| 3401,8 | 0,3 |
| 3402,8 | 0,3 |
| 3403,7 | 0,3 |
| 3404,7 | 0,3 |
| 3405,7 | 0,3 |
| 3406,6 | 0,3 |
| 3407,6 | 0,3 |
| 3408,6 | 0,3 |
| 3409,5 | 0,3 |
| 3410,5 | 0,3 |
| 3411,5 | 0,3 |
| 3412,4 | 0,3 |
| 3413,4 | 0,3 |
| 3414,4 | 0,3 |
| 3415,3 | 0,3 |
| 3416,3 | 0,3 |
| 3417,2 | 0,3 |
| 3418,2 | 0,3 |
| 3419,2 | 0,3 |
| 3420,1 | 0,3 |
| 3421,1 | 0,3 |
| 3422,1 | 0,3 |
| 3423,0 | 0,2 |
| 3424,0 | 0,2 |
| 3425,0 | 0,3 |
| 3425,9 | 0,3 |
| 3426,9 | 0,3 |
| 3427,8 | 0,3 |
| 3428,8 | 0,3 |
| 3429,8 | 0,3 |
| 3430,7 | 0,2 |
| 3431,7 | 0,2 |
| 3432,7 | 0,2 |
| 3433,6 | 0,2 |
| 3434,6 | 0,2 |
| 3435,6 | 0,2 |
| 3436,5 | 0,2 |
| 3437,5 | 0,2 |
| 3438,5 | 0,2 |
| 3439,4 | 0,2 |
| 3440,4 | 0,2 |
| 3441,3 | 0,2 |
| 3442,3 | 0,2 |
| 3443,3 | 0,2 |
| 3444,2 | 0,2 |
| 3445,2 | 0,2 |
| 3446,2 | 0,2 |
| 3447,1 | 0,2 |
| 3448,1 | 0,2 |
| 3449,1 | 0,2 |
| 3450,0 | 0,2 |
| 3451,0 | 0,2 |
| 3452,0 | 0,2 |
| 3452,9 | 0,2 |
| 3453,9 | 0,2 |
| 3454,8 | 0,2 |
| 3455,8 | 0,2 |
| 3456,8 | 0,2 |
| 3457,7 | 0,2 |
| 3458,7 | 0,2 |
| 3459,7 | 0,2 |
| 3460,6 | 0,2 |
| 3461,6 | 0,2 |
| 3462,6 | 0,2 |
| 3463,5 | 0,2 |
| 3464,5 | 0,2 |
| 3465,5 | 0,2 |
| 3466,4 | 0,2 |
| 3467,4 | 0,2 |
| 3468,3 | 0,2 |
| 3469,3 | 0,2 |
| 3470,3 | 0,2 |
| 3471,2 | 0,2 |
| 3472,2 | 0,2 |
| 3473,2 | 0,2 |
| 3474,1 | 0,2 |
| 3475,1 | 0,3 |
| 3476,1 | 0,3 |
| 3477,0 | 0,3 |
| 3478,0 | 0,3 |
| 3479,0 | 0,3 |
| 3479,9 | 0,3 |
| 3480,9 | 0,3 |
| 3481,8 | 0,3 |
| 3482,8 | 0,3 |
| 3483,8 | 0,3 |
| 3484,7 | 0,3 |
| 3485,7 | 0,3 |
| 3486,7 | 0,3 |
| 3487,6 | 0,3 |
| 3488,6 | 0,3 |
| 3489,6 | 0,3 |
| 3490,5 | 0,3 |
| 3491,5 | 0,3 |
| 3492,5 | 0,3 |
| 3493,4 | 0,3 |
| 3494,4 | 0,3 |
| 3495,3 | 0,3 |
| 3496,3 | 0,3 |
| 3497,3 | 0,3 |
| 3498,2 | 0,3 |
| 3499,2 | 0,3 |
| 3500,2 | 0,3 |
| 3501,1 | 0,3 |
| 3502,1 | 0,3 |
| 3503,1 | 0,3 |
| 3504,0 | 0,3 |
| 3505,0 | 0,3 |
| 3506,0 | 0,3 |
| 3506,9 | 0,3 |
| 3507,9 | 0,3 |
| 3508,8 | 0,3 |
| 3509,8 | 0,3 |
| 3510,8 | 0,3 |
| 3511,7 | 0,3 |
| 3512,7 | 0,3 |
| 3513,7 | 0,4 |
| 3514,6 | 0,4 |
| 3515,6 | 0,4 |
| 3516,6 | 0,4 |
| 3517,5 | 0,4 |
| 3518,5 | 0,4 |
| 3519,5 | 0,4 |
| 3520,4 | 0,4 |
| 3521,4 | 0,4 |
| 3522,3 | 0,4 |
| 3523,3 | 0,4 |
| 3524,3 | 0,4 |
| 3525,2 | 0,4 |
| 3526,2 | 0,4 |
| 3527,2 | 0,4 |
| 3528,1 | 0,4 |
| 3529,1 | 0,4 |
| 3530,1 | 0,4 |
| 3531,0 | 0,4 |
| 3532,0 | 0,4 |
| 3533,0 | 0,4 |
| 3533,9 | 0,4 |
| 3534,9 | 0,4 |
| 3535,8 | 0,5 |
| 3536,8 | 0,5 |
| 3537,8 | 0,5 |
| 3538,7 | 0,5 |
| 3539,7 | 0,5 |
| 3540,7 | 0,5 |
| 3541,6 | 0,5 |
| 3542,6 | 0,5 |
| 3543,6 | 0,5 |
| 3544,5 | 0,5 |
| 3545,5 | 0,5 |
| 3546,5 | 0,5 |
| 3547,4 | 0,5 |
| 3548,4 | 0,5 |
| 3549,3 | 0,5 |
| 3550,3 | 0,5 |
| 3551,3 | 0,5 |
| 3552,2 | 0,5 |
| 3553,2 | 0,5 |
| 3554,2 | 0,6 |
| 3555,1 | 0,6 |
| 3556,1 | 0,6 |
| 3557,1 | 0,6 |
| 3558,0 | 0,6 |
| 3559,0 | 0,6 |
| 3560,0 | 0,6 |
| 3560,9 | 0,6 |
| 3561,9 | 0,6 |
| 3562,8 | 0,6 |
| 3563,8 | 0,6 |
| 3564,8 | 0,6 |
| 3565,7 | 0,7 |
| 3566,7 | 0,7 |
| 3567,7 | 0,7 |
| 3568,6 | 0,7 |
| 3569,6 | 0,7 |
| 3570,6 | 0,7 |
| 3571,5 | 0,7 |
| 3572,5 | 0,7 |
| 3573,4 | 0,7 |
| 3574,4 | 0,8 |
| 3575,4 | 0,8 |
| 3576,3 | 0,8 |
| 3577,3 | 0,8 |
| 3578,3 | 0,8 |
| 3579,2 | 0,8 |
| 3580,2 | 0,9 |
| 3581,2 | 0,9 |
| 3582,1 | 0,9 |
| 3583,1 | 0,9 |
| 3584,1 | 0,9 |
| 3585,0 | 1,0 |
| 3586,0 | 1,0 |
| 3586,9 | 1,0 |
| 3587,9 | 1,0 |
| 3588,9 | 1,0 |
| 3589,8 | 1,1 |
| 3590,8 | 1,1 |
| 3591,8 | 1,1 |
| 3592,7 | 1,1 |
| 3593,7 | 1,2 |
| 3594,7 | 1,2 |
| 3595,6 | 1,2 |
| 3596,6 | 1,2 |
| 3597,6 | 1,3 |
| 3598,5 | 1,3 |
| 3599,5 | 1,3 |
| 3600,4 | 1,3 |
| 3601,4 | 1,4 |
| 3602,4 | 1,4 |
| 3603,3 | 1,4 |
| 3604,3 | 1,4 |
| 3605,3 | 1,5 |
| 3606,2 | 1,5 |
| 3607,2 | 1,5 |
| 3608,2 | 1,6 |
| 3609,1 | 1,6 |
| 3610,1 | 1,6 |
| 3611,1 | 1,7 |
| 3612,0 | 1,7 |
| 3613,0 | 1,7 |
| 3613,9 | 1,8 |
| 3614,9 | 1,8 |
| 3615,9 | 1,9 |
| 3616,8 | 1,9 |
| 3617,8 | 1,9 |
| 3618,8 | 2,0 |
| 3619,7 | 2,0 |
| 3620,7 | 2,1 |
| 3621,7 | 2,1 |
| 3622,6 | 2,2 |
| 3623,6 | 2,2 |
| 3624,6 | 2,3 |
| 3625,5 | 2,4 |
| 3626,5 | 2,4 |
| 3627,4 | 2,5 |
| 3628,4 | 2,6 |
| 3629,4 | 2,6 |
| 3630,3 | 2,7 |
| 3631,3 | 2,8 |
| 3632,3 | 2,9 |
| 3633,2 | 3,0 |
| 3634,2 | 3,0 |
| 3635,2 | 3,1 |
| 3636,1 | 3,2 |
| 3637,1 | 3,3 |
| 3638,1 | 3,4 |
| 3639,0 | 3,5 |
| 3640,0 | 3,6 |
| 3640,9 | 3,7 |
| 3641,9 | 3,8 |
| 3642,9 | 3,9 |
| 3643,8 | 4,0 |
| 3644,8 | 4,1 |
| 3645,8 | 4,2 |
| 3646,7 | 4,4 |
| 3647,7 | 4,5 |
| 3648,7 | 4,6 |
| 3649,6 | 4,8 |
| 3650,6 | 4,9 |
| 3651,6 | 5,1 |
| 3652,5 | 5,2 |
| 3653,5 | 5,4 |
| 3654,4 | 5,5 |
| 3655,4 | 5,7 |
| 3656,4 | 5,8 |
| 3657,3 | 6,0 |
| 3658,3 | 6,2 |
| 3659,3 | 6,4 |
| 3660,2 | 6,5 |
| 3661,2 | 6,7 |
| 3662,2 | 6,9 |
| 3663,1 | 7,1 |
| 3664,1 | 7,3 |
| 3665,1 | 7,6 |
| 3666,0 | 7,8 |
| 3667,0 | 8,0 |
| 3667,9 | 8,2 |
| 3668,9 | 8,5 |
| 3669,9 | 8,7 |
| 3670,8 | 8,9 |
| 3671,8 | 9,2 |
| 3672,8 | 9,4 |
| 3673,7 | 9,7 |
| 3674,7 | 9,9 |
| 3675,7 | 10,2 |
| 3676,6 | 10,4 |
| 3677,6 | 10,7 |
| 3678,6 | 10,9 |
| 3679,5 | 11,2 |
| 3680,5 | 11,4 |
| 3681,4 | 11,7 |
| 3682,4 | 11,9 |
| 3683,4 | 12,2 |
| 3684,3 | 12,4 |
| 3685,3 | 12,6 |
| 3686,3 | 12,8 |
| 3687,2 | 13,0 |
| 3688,2 | 13,1 |
| 3689,2 | 13,3 |
| 3690,1 | 13,5 |
| 3691,1 | 13,6 |
| 3692,0 | 13,8 |
| 3693,0 | 14,0 |
| 3694,0 | 14,2 |
| 3694,9 | 14,4 |
| 3695,9 | 14,6 |
| 3696,9 | 14,8 |
| 3697,8 | 15,1 |
| 3698,8 | 15,4 |
| 3699,8 | 15,7 |
| 3700,7 | 16,1 |
| 3701,7 | 16,5 |
| 3702,7 | 17,0 |
| 3703,6 | 17,4 |
| 3704,6 | 17,9 |
| 3705,5 | 18,4 |
| 3706,5 | 19,0 |
| 3707,5 | 19,6 |
| 3708,4 | 20,2 |
| 3709,4 | 20,9 |
| 3710,4 | 21,6 |
| 3711,3 | 22,4 |
| 3712,3 | 23,1 |
| 3713,3 | 24,0 |
| 3714,2 | 24,8 |
| 3715,2 | 25,7 |
| 3716,2 | 26,5 |
| 3717,1 | 27,4 |
| 3718,1 | 28,3 |
| 3719,0 | 29,2 |
| 3720,0 | 30,1 |
| 3721,0 | 30,9 |
| 3721,9 | 31,8 |
| 3722,9 | 32,6 |
| 3723,9 | 33,4 |
| 3724,8 | 34,1 |
| 3725,8 | 34,9 |
| 3726,8 | 35,6 |
| 3727,7 | 36,3 |
| 3728,7 | 37,1 |
| 3729,7 | 37,8 |
| 3730,6 | 38,4 |
| 3731,6 | 39,1 |
| 3732,5 | 39,7 |
| 3733,5 | 40,3 |
| 3734,5 | 40,9 |
| 3735,4 | 41,4 |
| 3736,4 | 42,0 |
| 3737,4 | 42,5 |
| 3738,3 | 43,0 |
| 3739,3 | 43,4 |
| 3740,3 | 43,9 |
| 3741,2 | 44,3 |
| 3742,2 | 44,7 |
| 3743,2 | 45,1 |
| 3744,1 | 45,6 |
| 3745,1 | 46,0 |
| 3746,0 | 46,4 |
| 3747,0 | 46,8 |
| 3748,0 | 47,2 |
| 3748,9 | 47,6 |
| 3749,9 | 48,0 |
| 3750,9 | 48,4 |
| 3751,8 | 48,8 |
| 3752,8 | 49,2 |
| 3753,8 | 49,5 |
| 3754,7 | 49,9 |
| 3755,7 | 50,2 |
| 3756,7 | 50,6 |
| 3757,6 | 50,9 |
| 3758,6 | 51,3 |
| 3759,5 | 51,6 |
| 3760,5 | 51,9 |
| 3761,5 | 52,3 |
| 3762,4 | 52,6 |
| 3763,4 | 53,0 |
| 3764,4 | 53,4 |
| 3765,3 | 53,7 |
| 3766,3 | 54,1 |
| 3767,3 | 54,4 |
| 3768,2 | 54,7 |
| 3769,2 | 54,9 |
| 3770,2 | 55,2 |
| 3771,1 | 55,5 |
| 3772,1 | 55,8 |
| 3773,0 | 56,1 |
| 3774,0 | 56,4 |
| 3775,0 | 56,7 |
| 3775,9 | 57,0 |
| 3776,9 | 57,2 |
| 3777,9 | 57,5 |
| 3778,8 | 57,8 |
| 3779,8 | 58,1 |
| 3780,8 | 58,4 |
| 3781,7 | 58,7 |
| 3782,7 | 59,0 |
| 3783,7 | 59,2 |
| 3784,6 | 59,5 |
| 3785,6 | 59,8 |
| 3786,5 | 60,0 |
| 3787,5 | 60,3 |
| 3788,5 | 60,5 |
| 3789,4 | 60,7 |
| 3790,4 | 60,9 |
| 3791,4 | 61,2 |
| 3792,3 | 61,4 |
| 3793,3 | 61,6 |
| 3794,3 | 61,9 |
| 3795,2 | 62,1 |
| 3796,2 | 62,3 |
| 3797,2 | 62,6 |
| 3798,1 | 62,8 |
| 3799,1 | 63,0 |
| 3800,0 | 63,3 |
| 3801,0 | 63,5 |
| 3802,0 | 63,8 |
| 3802,9 | 64,0 |
| 3803,9 | 64,2 |
| 3804,9 | 64,4 |
| 3805,8 | 64,6 |
| 3806,8 | 64,8 |
| 3807,8 | 65,0 |
| 3808,7 | 65,2 |
| 3809,7 | 65,4 |
| 3810,7 | 65,6 |
| 3811,6 | 65,8 |
| 3812,6 | 66,0 |
| 3813,5 | 66,2 |
| 3814,5 | 66,5 |
| 3815,5 | 66,7 |
| 3816,4 | 66,9 |
| 3817,4 | 67,2 |
| 3818,4 | 67,4 |
| 3819,3 | 67,6 |
| 3820,3 | 67,9 |
| 3821,3 | 68,1 |
| 3822,2 | 68,3 |
| 3823,2 | 68,5 |
| 3824,1 | 68,7 |
| 3825,1 | 68,9 |
| 3826,1 | 69,1 |
| 3827,0 | 69,3 |
| 3828,0 | 69,4 |
| 3829,0 | 69,6 |
| 3829,9 | 69,8 |
| 3830,9 | 70,0 |
| 3831,9 | 70,2 |
| 3832,8 | 70,4 |
| 3833,8 | 70,6 |
| 3834,8 | 70,9 |
| 3835,7 | 71,2 |
| 3836,7 | 71,4 |
| 3837,6 | 71,7 |
| 3838,6 | 71,9 |
| 3839,6 | 72,2 |
| 3840,5 | 72,3 |
| 3841,5 | 72,5 |
| 3842,5 | 72,6 |
| 3843,4 | 72,8 |
| 3844,4 | 72,9 |
| 3845,4 | 73,1 |
| 3846,3 | 73,2 |
| 3847,3 | 73,4 |
| 3848,3 | 73,6 |
| 3849,2 | 73,9 |
| 3850,2 | 74,1 |
| 3851,1 | 74,3 |
| 3852,1 | 74,6 |
| 3853,1 | 74,8 |
| 3854,0 | 75,0 |
| 3855,0 | 75,2 |
| 3856,0 | 75,5 |
| 3856,9 | 75,7 |
| 3857,9 | 75,9 |
| 3858,9 | 76,0 |
| 3859,8 | 76,2 |
| 3860,8 | 76,3 |
| 3861,8 | 76,4 |
| 3862,7 | 76,5 |
| 3863,7 | 76,7 |
| 3864,6 | 76,9 |
| 3865,6 | 77,1 |
| 3866,6 | 77,4 |
| 3867,5 | 77,7 |
| 3868,5 | 77,9 |
| 3869,5 | 78,1 |
| 3870,4 | 78,3 |
| 3871,4 | 78,5 |
| 3872,4 | 78,7 |
| 3873,3 | 78,8 |
| 3874,3 | 79,0 |
| 3875,3 | 79,1 |
| 3876,2 | 79,3 |
| 3877,2 | 79,5 |
| 3878,1 | 79,7 |
| 3879,1 | 79,9 |
| 3880,1 | 80,1 |
| 3881,0 | 80,2 |
| 3882,0 | 80,4 |
| 3883,0 | 80,6 |
| 3883,9 | 80,8 |
| 3884,9 | 81,1 |
| 3885,9 | 81,3 |
| 3886,8 | 81,5 |
| 3887,8 | 81,7 |
| 3888,8 | 81,9 |
| 3889,7 | 82,0 |
| 3890,7 | 82,2 |
| 3891,6 | 82,3 |
| 3892,6 | 82,4 |
| 3893,6 | 82,6 |
| 3894,5 | 82,7 |
| 3895,5 | 82,9 |
| 3896,5 | 83,0 |
| 3897,4 | 83,2 |
| 3898,4 | 83,4 |
| 3899,4 | 83,6 |
| 3900,3 | 83,8 |
| 3901,3 | 84,0 |
| 3902,3 | 84,2 |
| 3903,2 | 84,4 |
| 3904,2 | 84,6 |
| 3905,1 | 84,7 |
| 3906,1 | 84,9 |
| 3907,1 | 85,1 |
| 3908,0 | 85,2 |
| 3909,0 | 85,4 |
| 3910,0 | 85,5 |
| 3910,9 | 85,6 |
| 3911,9 | 85,8 |
| 3912,9 | 86,0 |
| 3913,8 | 86,1 |
| 3914,8 | 86,3 |
| 3915,8 | 86,5 |
| 3916,7 | 86,7 |
| 3917,7 | 87,0 |
| 3918,6 | 87,2 |
| 3919,6 | 87,3 |
| 3920,6 | 87,5 |
| 3921,5 | 87,6 |
| 3922,5 | 87,8 |
| 3923,5 | 87,9 |
| 3924,4 | 88,0 |
| 3925,4 | 88,2 |
| 3926,4 | 88,3 |
| 3927,3 | 88,5 |
| 3928,3 | 88,6 |
| 3929,3 | 88,8 |
| 3930,2 | 89,0 |
| 3931,2 | 89,1 |
| 3932,1 | 89,3 |
| 3933,1 | 89,5 |
| 3934,1 | 89,6 |
| 3935,0 | 89,8 |
| 3936,0 | 89,9 |
| 3937,0 | 90,1 |
| 3937,9 | 90,3 |
| 3938,9 | 90,4 |
| 3939,9 | 90,6 |
| 3940,8 | 90,7 |
| 3941,8 | 90,9 |
| 3942,8 | 91,0 |
| 3943,7 | 91,2 |
| 3944,7 | 91,4 |
| 3945,6 | 91,6 |
| 3946,6 | 91,7 |
| 3947,6 | 91,9 |
| 3948,5 | 92,1 |
| 3949,5 | 92,2 |
| 3950,5 | 92,4 |
| 3951,4 | 92,5 |
| 3952,4 | 92,7 |
| 3953,4 | 92,8 |
| 3954,3 | 93,0 |
| 3955,3 | 93,1 |
| 3956,3 | 93,3 |
| 3957,2 | 93,5 |
| 3958,2 | 93,6 |
| 3959,1 | 93,8 |
| 3960,1 | 93,9 |
| 3961,1 | 94,1 |
| 3962,0 | 94,3 |
| 3963,0 | 94,4 |
| 3964,0 | 94,6 |
| 3964,9 | 94,7 |
| 3965,9 | 94,9 |
| 3966,9 | 95,0 |
| 3967,8 | 95,2 |
| 3968,8 | 95,3 |
| 3969,7 | 95,5 |
| 3970,7 | 95,6 |
| 3971,7 | 95,8 |
| 3972,6 | 95,9 |
| 3973,6 | 96,1 |
| 3974,6 | 96,2 |
| 3975,5 | 96,4 |
| 3976,5 | 96,6 |
| 3977,5 | 96,7 |
| 3978,4 | 96,9 |
| 3979,4 | 97,0 |
| 3980,4 | 97,2 |
| 3981,3 | 97,3 |
| 3982,3 | 97,5 |
| 3983,2 | 97,6 |
| 3984,2 | 97,8 |
| 3985,2 | 97,9 |
| 3986,1 | 98,1 |
| 3987,1 | 98,2 |
| 3988,1 | 98,4 |
| 3989,0 | 98,5 |
| 3990,0 | 98,7 |
| 3991,0 | 98,8 |
| 3991,9 | 99,0 |
| 3992,9 | 99,1 |
| 3993,9 | 99,3 |
| 3994,8 | 99,4 |
| 3995,8 | 99,5 |
| 3996,7 | 99,6 |
| 3997,7 | 99,8 |
| 3998,7 | 99,9 |
| 3999,6 | 100,0 |

Table S2. Data XRD of LDH sample.

| 2Ɵ Degree | Intensity U.A. |
| --- | --- |
| 4,0 | 0,0 |
| 4,0 | 88,7 |
| 4,0 | 87,3 |
| 4,1 | 35,2 |
| 4,1 | 64,0 |
| 4,1 | 39,2 |
| 4,1 | 46,5 |
| 4,1 | 28,5 |
| 4,2 | 98,6 |
| 4,2 | 12,4 |
| 4,2 | 60,9 |
| 4,2 | 2,0 |
| 4,2 | 4,8 |
| 4,3 | 14,1 |
| 4,3 | 39,0 |
| 4,3 | 79,6 |
| 4,3 | 67,6 |
| 4,3 | 52,2 |
| 4,4 | 0,0 |
| 4,4 | 11,7 |
| 4,4 | 34,5 |
| 4,4 | 51,4 |
| 4,4 | 25,5 |
| 4,5 | 18,2 |
| 4,5 | 2,2 |
| 4,5 | -18,0 |
| 4,5 | 0,0 |
| 4,5 | 3,0 |
| 4,6 | 46,8 |
| 4,6 | 56,3 |
| 4,6 | 19,0 |
| 4,6 | 0,0 |
| 4,6 | -23,0 |
| 4,7 | -8,0 |
| 4,7 | 18,0 |
| 4,7 | 0,0 |
| 4,7 | 6,0 |
| 4,7 | 25,2 |
| 4,8 | 40,3 |
| 4,8 | 0,0 |
| 4,8 | -49,0 |
| 4,8 | -62,0 |
| 4,8 | -59,0 |
| 4,9 | -36,0 |
| 4,9 | -48,0 |
| 4,9 | -72,0 |
| 4,9 | -82,0 |
| 4,9 | -78,0 |
| 5,0 | -50,0 |
| 5,0 | -39,0 |
| 5,0 | 0,0 |
| 5,0 | -47,0 |
| 5,0 | -43,0 |
| 5,1 | -64,0 |
| 5,1 | -65,0 |
| 5,1 | -62,0 |
| 5,1 | -35,0 |
| 5,1 | 48,0 |
| 5,2 | 0,0 |
| 5,2 | -19,0 |
| 5,2 | -50,0 |
| 5,2 | -39,0 |
| 5,2 | -15,2 |
| 5,3 | 0,0 |
| 5,3 | -8,0 |
| 5,3 | 20,8 |
| 5,3 | 5,9 |
| 5,3 | 0,0 |
| 5,4 | -21,0 |
| 5,4 | -41,0 |
| 5,4 | -12,4 |
| 5,4 | -15,0 |
| 5,4 | 26,6 |
| 5,5 | 0,0 |
| 5,5 | -10,0 |
| 5,5 | -40,0 |
| 5,5 | -44,0 |
| 5,5 | -35,0 |
| 5,6 | -15,3 |
| 5,6 | 0,0 |
| 5,6 | 17,2 |
| 5,6 | 0,0 |
| 5,6 | -4,1 |
| 5,7 | -3,7 |
| 5,7 | -16,0 |
| 5,7 | -0,6 |
| 5,7 | -9,9 |
| 5,7 | -19,2 |
| 5,8 | 20,5 |
| 5,8 | 6,6 |
| 5,8 | 0,0 |
| 5,8 | -23,0 |
| 5,8 | -19,5 |
| 5,9 | -38,0 |
| 5,9 | -22,7 |
| 5,9 | -8,2 |
| 5,9 | 7,4 |
| 5,9 | 9,1 |
| 6,0 | 0,0 |
| 6,0 | -19,8 |
| 6,0 | 33,3 |
| 6,0 | 20,6 |
| 6,0 | 0,0 |
| 6,1 | -9,0 |
| 6,1 | 10,0 |
| 6,1 | 25,6 |
| 6,1 | 30,8 |
| 6,1 | 0,0 |
| 6,2 | 19,2 |
| 6,2 | 22,6 |
| 6,2 | -0,7 |
| 6,2 | 0,0 |
| 6,2 | -22,0 |
| 6,3 | 0,0 |
| 6,3 | -12,5 |
| 6,3 | 0,0 |
| 6,3 | -2,0 |
| 6,3 | 41,9 |
| 6,4 | 38,9 |
| 6,4 | 64,3 |
| 6,4 | 8,6 |
| 6,4 | 0,0 |
| 6,4 | -25,4 |
| 6,5 | -14,9 |
| 6,5 | -8,9 |
| 6,5 | 0,0 |
| 6,5 | -25,0 |
| 6,5 | -25,9 |
| 6,6 | 0,0 |
| 6,6 | 4,8 |
| 6,6 | -40,0 |
| 6,6 | -37,0 |
| 6,6 | -16,2 |
| 6,7 | 27,2 |
| 6,7 | 27,0 |
| 6,7 | 87,7 |
| 6,7 | 6,5 |
| 6,7 | -3,6 |
| 6,8 | 73,4 |
| 6,8 | 2,0 |
| 6,8 | -15,0 |
| 6,8 | 2,6 |
| 6,8 | -26,0 |
| 6,9 | -24,0 |
| 6,9 | 8,3 |
| 6,9 | -15,6 |
| 6,9 | 16,6 |
| 6,9 | 1,7 |
| 7,0 | 4,1 |
| 7,0 | 1,5 |
| 7,0 | -16,6 |
| 7,0 | 9,5 |
| 7,0 | 13,2 |
| 7,1 | 8,2 |
| 7,1 | 36,1 |
| 7,1 | -14,7 |
| 7,1 | 7,1 |
| 7,1 | 13,7 |
| 7,2 | 11,0 |
| 7,2 | 3,9 |
| 7,2 | 28,2 |
| 7,2 | 22,9 |
| 7,2 | 1,2 |
| 7,3 | -22,0 |
| 7,3 | -20,0 |
| 7,3 | -2,8 |
| 7,3 | 19,9 |
| 7,3 | 2,0 |
| 7,4 | 12,4 |
| 7,4 | -26,8 |
| 7,4 | -2,6 |
| 7,4 | 6,4 |
| 7,4 | 14,9 |
| 7,5 | 32,3 |
| 7,5 | -18,2 |
| 7,5 | -24,0 |
| 7,5 | -26,0 |
| 7,5 | 0,0 |
| 7,6 | 2,3 |
| 7,6 | -14,7 |
| 7,6 | -7,7 |
| 7,6 | -11,3 |
| 7,6 | -7,9 |
| 7,7 | 9,9 |
| 7,7 | -10,0 |
| 7,7 | 17,5 |
| 7,7 | 30,6 |
| 7,7 | 23,0 |
| 7,8 | 44,4 |
| 7,8 | 27,3 |
| 7,8 | 0,0 |
| 7,8 | -6,1 |
| 7,8 | 4,6 |
| 7,9 | -5,2 |
| 7,9 | -8,8 |
| 7,9 | -19,5 |
| 7,9 | 56,5 |
| 7,9 | 10,0 |
| 8,0 | 62,0 |
| 8,0 | 2,0 |
| 8,0 | 53,6 |
| 8,0 | -30,0 |
| 8,0 | -9,0 |
| 8,1 | 2,4 |
| 8,1 | 41,2 |
| 8,1 | 8,1 |
| 8,1 | 16,8 |
| 8,1 | -6,2 |
| 8,2 | -30,0 |
| 8,2 | -27,0 |
| 8,2 | 12,7 |
| 8,2 | 28,9 |
| 8,2 | 37,3 |
| 8,3 | 31,5 |
| 8,3 | -1,8 |
| 8,3 | 1,5 |
| 8,3 | 23,5 |
| 8,3 | 31,3 |
| 8,4 | -14,9 |
| 8,4 | 24,2 |
| 8,4 | -10,0 |
| 8,4 | -17,3 |
| 8,4 | 24,9 |
| 8,5 | -21,6 |
| 8,5 | 9,2 |
| 8,5 | 10,9 |
| 8,5 | 35,1 |
| 8,5 | 49,6 |
| 8,6 | -8,6 |
| 8,6 | 1,0 |
| 8,6 | 28,0 |
| 8,6 | 18,0 |
| 8,6 | -4,1 |
| 8,7 | -19,0 |
| 8,7 | -23,3 |
| 8,7 | 5,6 |
| 8,7 | 20,9 |
| 8,7 | -8,1 |
| 8,8 | 25,9 |
| 8,8 | -23,8 |
| 8,8 | -27,0 |
| 8,8 | -30,0 |
| 8,8 | -18,8 |
| 8,9 | 14,5 |
| 8,9 | 12,9 |
| 8,9 | 29,1 |
| 8,9 | -5,0 |
| 8,9 | 15,0 |
| 9,0 | 3,7 |
| 9,0 | 47,8 |
| 9,0 | 25,0 |
| 9,0 | 7,0 |
| 9,0 | 13,6 |
| 9,1 | 22,8 |
| 9,1 | 52,6 |
| 9,1 | 14,0 |
| 9,1 | 15,2 |
| 9,1 | -1,9 |
| 9,2 | -8,0 |
| 9,2 | 17,7 |
| 9,2 | -11,0 |
| 9,2 | -15,8 |
| 9,2 | 74,0 |
| 9,3 | 8,0 |
| 9,3 | -4,2 |
| 9,3 | 11,3 |
| 9,3 | 57,4 |
| 9,3 | 33,0 |
| 9,4 | 94,9 |
| 9,4 | -10,6 |
| 9,4 | 4,7 |
| 9,4 | 36,4 |
| 9,4 | 7,7 |
| 9,5 | 20,7 |
| 9,5 | 81,9 |
| 9,5 | 20,4 |
| 9,5 | 5,5 |
| 9,5 | 11,3 |
| 9,6 | -22,5 |
| 9,6 | 22,4 |
| 9,6 | 56,2 |
| 9,6 | -2,0 |
| 9,6 | 4,0 |
| 9,7 | 58,3 |
| 9,7 | 86,8 |
| 9,7 | 78,5 |
| 9,7 | 27,6 |
| 9,7 | 74,0 |
| 9,8 | 55,4 |
| 9,8 | 55,6 |
| 9,8 | 44,7 |
| 9,8 | 93,7 |
| 9,8 | 118,6 |
| 9,9 | 69,3 |
| 9,9 | 75,8 |
| 9,9 | 60,7 |
| 9,9 | 81,5 |
| 9,9 | 126,6 |
| 10,0 | 126,0 |
| 10,0 | 167,6 |
| 10,0 | 152,9 |
| 10,0 | 102,6 |
| 10,0 | 146,1 |
| 10,1 | 137,5 |
| 10,1 | 110,8 |
| 10,1 | 207,2 |
| 10,1 | 161,9 |
| 10,1 | 166,3 |
| 10,2 | 190,2 |
| 10,2 | 197,0 |
| 10,2 | 217,4 |
| 10,2 | 168,4 |
| 10,2 | 280,1 |
| 10,3 | 173,0 |
| 10,3 | 199,7 |
| 10,3 | 223,1 |
| 10,3 | 227,7 |
| 10,3 | 239,5 |
| 10,4 | 284,4 |
| 10,4 | 306,2 |
| 10,4 | 284,6 |
| 10,4 | 243,6 |
| 10,4 | 300,3 |
| 10,5 | 316,9 |
| 10,5 | 348,3 |
| 10,5 | 331,1 |
| 10,5 | 422,0 |
| 10,5 | 274,7 |
| 10,6 | 356,7 |
| 10,6 | 383,9 |
| 10,6 | 496,9 |
| 10,6 | 438,8 |
| 10,6 | 401,5 |
| 10,7 | 396,9 |
| 10,7 | 493,6 |
| 10,7 | 432,6 |
| 10,7 | 520,5 |
| 10,7 | 520,8 |
| 10,8 | 536,4 |
| 10,8 | 565,0 |
| 10,8 | 543,8 |
| 10,8 | 666,4 |
| 10,8 | 666,4 |
| 10,9 | 633,7 |
| 10,9 | 658,7 |
| 10,9 | 671,2 |
| 10,9 | 618,5 |
| 10,9 | 670,2 |
| 11,0 | 715,2 |
| 11,0 | 765,7 |
| 11,0 | 678,5 |
| 11,0 | 797,9 |
| 11,0 | 786,5 |
| 11,1 | 758,2 |
| 11,1 | 874,9 |
| 11,1 | 970,7 |
| 11,1 | 906,5 |
| 11,1 | 856,5 |
| 11,2 | 952,3 |
| 11,2 | 978,5 |
| 11,2 | 964,9 |
| 11,2 | 996,8 |
| 11,2 | 980,9 |
| 11,3 | 999,8 |
| 11,3 | 1089,5 |
| 11,3 | 1099,9 |
| 11,3 | 1073,6 |
| 11,3 | 1028,3 |
| 11,4 | 1075,4 |
| 11,4 | 1085,3 |
| 11,4 | 1132,2 |
| 11,4 | 1158,4 |
| 11,4 | 1102,5 |
| 11,5 | 1064,9 |
| 11,5 | 1041,7 |
| 11,5 | 1106,2 |
| 11,5 | 1046,5 |
| 11,5 | 961,1 |
| 11,6 | 1047,6 |
| 11,6 | 1040,0 |
| 11,6 | 968,6 |
| 11,6 | 904,1 |
| 11,6 | 903,3 |
| 11,7 | 836,7 |
| 11,7 | 878,2 |
| 11,7 | 702,4 |
| 11,7 | 822,4 |
| 11,7 | 711,6 |
| 11,8 | 754,6 |
| 11,8 | 653,0 |
| 11,8 | 703,6 |
| 11,8 | 602,6 |
| 11,8 | 528,9 |
| 11,9 | 611,2 |
| 11,9 | 611,4 |
| 11,9 | 484,9 |
| 11,9 | 481,2 |
| 11,9 | 483,2 |
| 12,0 | 458,6 |
| 12,0 | 395,4 |
| 12,0 | 411,1 |
| 12,0 | 397,7 |
| 12,0 | 394,9 |
| 12,1 | 394,7 |
| 12,1 | 264,3 |
| 12,1 | 293,6 |
| 12,1 | 361,8 |
| 12,1 | 316,7 |
| 12,2 | 291,2 |
| 12,2 | 298,3 |
| 12,2 | 251,7 |
| 12,2 | 223,1 |
| 12,2 | 251,9 |
| 12,3 | 225,5 |
| 12,3 | 227,4 |
| 12,3 | 208,7 |
| 12,3 | 194,0 |
| 12,3 | 193,3 |
| 12,4 | 183,1 |
| 12,4 | 179,3 |
| 12,4 | 171,0 |
| 12,4 | 158,1 |
| 12,4 | 139,7 |
| 12,5 | 115,8 |
| 12,5 | 132,4 |
| 12,5 | 172,0 |
| 12,5 | 122,8 |
| 12,5 | 105,3 |
| 12,6 | 143,1 |
| 12,6 | 152,3 |
| 12,6 | 129,6 |
| 12,6 | 115,5 |
| 12,6 | 54,7 |
| 12,7 | 97,3 |
| 12,7 | 85,1 |
| 12,7 | 125,7 |
| 12,7 | 129,5 |
| 12,7 | 132,0 |
| 12,8 | 81,3 |
| 12,8 | 119,4 |
| 12,8 | 90,2 |
| 12,8 | 38,5 |
| 12,8 | 74,9 |
| 12,9 | 118,5 |
| 12,9 | 54,0 |
| 12,9 | 45,6 |
| 12,9 | 98,0 |
| 12,9 | 93,8 |
| 13,0 | 54,4 |
| 13,0 | 79,1 |
| 13,0 | 30,9 |
| 13,0 | 60,0 |
| 13,0 | 57,3 |
| 13,1 | 107,2 |
| 13,1 | 67,4 |
| 13,1 | 35,0 |
| 13,1 | 45,8 |
| 13,1 | 79,1 |
| 13,2 | 119,9 |
| 13,2 | 0,2 |
| 13,2 | 31,5 |
| 13,2 | 88,2 |
| 13,2 | 4,9 |
| 13,3 | 11,8 |
| 13,3 | 11,3 |
| 13,3 | 67,9 |
| 13,3 | 106,2 |
| 13,3 | 49,3 |
| 13,4 | -5,7 |
| 13,4 | 9,6 |
| 13,4 | 125,2 |
| 13,4 | 81,8 |
| 13,4 | 38,4 |
| 13,5 | 1,5 |
| 13,5 | 10,3 |
| 13,5 | 43,0 |
| 13,5 | 58,3 |
| 13,5 | 51,8 |
| 13,6 | 35,7 |
| 13,6 | 44,0 |
| 13,6 | 17,2 |
| 13,6 | 37,2 |
| 13,6 | 32,8 |
| 13,7 | 16,0 |
| 13,7 | 27,1 |
| 13,7 | 82,1 |
| 13,7 | 66,8 |
| 13,7 | -7,0 |
| 13,8 | -9,4 |
| 13,8 | 41,0 |
| 13,8 | 59,7 |
| 13,8 | 45,2 |
| 13,8 | -18,9 |
| 13,9 | 31,2 |
| 13,9 | 13,9 |
| 13,9 | 16,0 |
| 13,9 | 37,3 |
| 13,9 | 76,2 |
| 14,0 | 7,7 |
| 14,0 | 28,2 |
| 14,0 | 18,6 |
| 14,0 | 21,1 |
| 14,0 | 26,9 |
| 14,1 | 31,8 |
| 14,1 | 23,5 |
| 14,1 | 19,9 |
| 14,1 | -10,6 |
| 14,1 | 17,2 |
| 14,2 | 23,6 |
| 14,2 | 46,0 |
| 14,2 | 17,2 |
| 14,2 | 114,7 |
| 14,2 | 48,6 |
| 14,3 | -2,9 |
| 14,3 | -33,5 |
| 14,3 | 49,7 |
| 14,3 | 44,0 |
| 14,3 | 118,7 |
| 14,4 | 40,1 |
| 14,4 | -30,7 |
| 14,4 | 3,9 |
| 14,4 | -14,4 |
| 14,4 | 72,4 |
| 14,5 | 41,8 |
| 14,5 | -24,8 |
| 14,5 | -14,4 |
| 14,5 | -4,2 |
| 14,5 | 26,1 |
| 14,6 | 27,2 |
| 14,6 | 4,2 |
| 14,6 | -7,3 |
| 14,6 | 72,3 |
| 14,6 | -1,8 |
| 14,7 | -20,0 |
| 14,7 | -11,1 |
| 14,7 | -12,4 |
| 14,7 | 9,3 |
| 14,7 | 19,2 |
| 14,8 | -0,6 |
| 14,8 | -3,1 |
| 14,8 | 25,3 |
| 14,8 | -8,0 |
| 14,8 | -31,4 |
| 14,9 | -9,9 |
| 14,9 | 15,5 |
| 14,9 | -6,2 |
| 14,9 | -35,1 |
| 14,9 | -26,2 |
| 15,0 | -18,2 |
| 15,0 | 0,9 |
| 15,0 | 20,7 |
| 15,0 | -9,3 |
| 15,0 | -26,1 |
| 15,1 | 11,2 |
| 15,1 | 0,9 |
| 15,1 | -27,4 |
| 15,1 | -20,7 |
| 15,1 | -14,9 |
| 15,2 | -28,8 |
| 15,2 | -20,3 |
| 15,2 | -30,2 |
| 15,2 | -18,9 |
| 15,2 | -3,4 |
| 15,3 | -3,8 |
| 15,3 | -27,0 |
| 15,3 | -30,0 |
| 15,3 | -2,2 |
| 15,3 | -26,8 |
| 15,4 | -21,0 |
| 15,4 | -0,4 |
| 15,4 | -32,8 |
| 15,4 | -29,0 |
| 15,4 | -19,8 |
| 15,5 | -3,4 |
| 15,5 | 33,7 |
| 15,5 | 28,0 |
| 15,5 | -17,0 |
| 15,5 | -14,9 |
| 15,6 | -39,0 |
| 15,6 | 5,5 |
| 15,6 | -27,9 |
| 15,6 | 31,7 |
| 15,6 | 47,7 |
| 15,7 | -18,0 |
| 15,7 | -21,0 |
| 15,7 | 18,8 |
| 15,7 | 32,0 |
| 15,7 | -36,7 |
| 15,8 | -18,0 |
| 15,8 | 16,3 |
| 15,8 | -2,7 |
| 15,8 | -1,5 |
| 15,8 | -14,0 |
| 15,9 | -46,0 |
| 15,9 | -43,0 |
| 15,9 | -23,2 |
| 15,9 | -1,6 |
| 15,9 | -43,4 |
| 16,0 | -34,0 |
| 16,0 | -21,9 |
| 16,0 | -42,5 |
| 16,0 | -40,0 |
| 16,0 | -19,6 |
| 16,1 | -28,4 |
| 16,1 | -36,0 |
| 16,1 | -37,2 |
| 16,1 | -30,3 |
| 16,1 | 14,4 |
| 16,2 | 9,3 |
| 16,2 | 1,5 |
| 16,2 | 13,3 |
| 16,2 | 3,2 |
| 16,2 | -16,0 |
| 16,3 | -11,6 |
| 16,3 | 13,1 |
| 16,3 | -32,7 |
| 16,3 | -41,6 |
| 16,3 | -25,2 |
| 16,4 | -7,8 |
| 16,4 | -3,1 |
| 16,4 | -3,0 |
| 16,4 | 36,4 |
| 16,4 | -23,4 |
| 16,5 | -27,9 |
| 16,5 | -12,4 |
| 16,5 | -19,6 |
| 16,5 | -19,9 |
| 16,5 | 0,1 |
| 16,6 | -19,6 |
| 16,6 | -13,3 |
| 16,6 | -0,3 |
| 16,6 | 3,4 |
| 16,6 | -20,6 |
| 16,7 | 44,9 |
| 16,7 | 6,0 |
| 16,7 | -19,0 |
| 16,7 | 56,0 |
| 16,7 | 38,6 |
| 16,8 | -12,0 |
| 16,8 | -29,0 |
| 16,8 | 29,4 |
| 16,8 | -17,1 |
| 16,8 | -16,0 |
| 16,9 | -37,5 |
| 16,9 | 20,1 |
| 16,9 | -42,8 |
| 16,9 | 15,0 |
| 16,9 | -42,2 |
| 17,0 | 12,7 |
| 17,0 | 47,8 |
| 17,0 | 18,8 |
| 17,0 | 41,7 |
| 17,0 | -21,0 |
| 17,1 | -26,6 |
| 17,1 | 1,4 |
| 17,1 | 32,7 |
| 17,1 | 1,9 |
| 17,1 | -10,6 |
| 17,2 | 20,1 |
| 17,2 | -8,4 |
| 17,2 | -21,0 |
| 17,2 | 5,5 |
| 17,2 | 7,4 |
| 17,3 | 9,3 |
| 17,3 | -24,3 |
| 17,3 | 6,2 |
| 17,3 | 54,4 |
| 17,3 | -2,0 |
| 17,4 | -20,0 |
| 17,4 | -30,6 |
| 17,4 | 40,4 |
| 17,4 | -4,5 |
| 17,4 | -28,0 |
| 17,5 | -43,6 |
| 17,5 | -4,3 |
| 17,5 | 8,2 |
| 17,5 | 26,9 |
| 17,5 | 18,3 |
| 17,6 | -42,0 |
| 17,6 | -35,3 |
| 17,6 | -37,6 |
| 17,6 | -31,8 |
| 17,6 | 16,8 |
| 17,7 | 18,0 |
| 17,7 | 3,3 |
| 17,7 | 40,5 |
| 17,7 | 25,3 |
| 17,7 | -8,4 |
| 17,8 | -23,6 |
| 17,8 | 65,4 |
| 17,8 | 70,3 |
| 17,8 | -11,2 |
| 17,8 | -31,5 |
| 17,9 | 22,9 |
| 17,9 | 38,0 |
| 17,9 | -6,9 |
| 17,9 | 14,6 |
| 17,9 | -18,0 |
| 18,0 | 42,8 |
| 18,0 | 46,2 |
| 18,0 | 92,6 |
| 18,0 | 71,0 |
| 18,0 | 55,9 |
| 18,1 | 63,3 |
| 18,1 | 70,0 |
| 18,1 | 92,9 |
| 18,1 | 91,6 |
| 18,1 | 136,9 |
| 18,2 | 143,3 |
| 18,2 | 152,0 |
| 18,2 | 163,5 |
| 18,2 | 154,4 |
| 18,2 | 193,7 |
| 18,3 | 213,3 |
| 18,3 | 227,1 |
| 18,3 | 277,9 |
| 18,3 | 309,6 |
| 18,3 | 261,4 |
| 18,4 | 306,4 |
| 18,4 | 353,2 |
| 18,4 | 298,0 |
| 18,4 | 261,4 |
| 18,4 | 210,2 |
| 18,5 | 256,6 |
| 18,5 | 200,2 |
| 18,5 | 125,7 |
| 18,5 | 111,8 |
| 18,5 | 144,0 |
| 18,6 | 140,8 |
| 18,6 | 140,8 |
| 18,6 | 88,6 |
| 18,6 | 110,4 |
| 18,6 | 153,7 |
| 18,7 | 101,5 |
| 18,7 | 49,4 |
| 18,7 | 88,2 |
| 18,7 | 120,8 |
| 18,7 | 65,6 |
| 18,8 | 66,4 |
| 18,8 | 58,3 |
| 18,8 | 29,3 |
| 18,8 | 15,8 |
| 18,8 | -8,4 |
| 18,9 | -18,0 |
| 18,9 | -10,5 |
| 18,9 | 42,2 |
| 18,9 | 59,0 |
| 18,9 | 21,3 |
| 19,0 | -19,8 |
| 19,0 | -28,0 |
| 19,0 | -15,4 |
| 19,0 | 3,3 |
| 19,0 | 20,9 |
| 19,1 | 19,5 |
| 19,1 | 5,8 |
| 19,1 | 7,1 |
| 19,1 | -21,9 |
| 19,1 | -30,1 |
| 19,2 | 17,0 |
| 19,2 | -5,2 |
| 19,2 | 15,0 |
| 19,2 | 15,4 |
| 19,2 | -29,0 |
| 19,3 | -31,4 |
| 19,3 | -36,0 |
| 19,3 | -5,8 |
| 19,3 | 4,5 |
| 19,3 | -9,7 |
| 19,4 | -12,8 |
| 19,4 | -11,9 |
| 19,4 | 4,0 |
| 19,4 | 32,2 |
| 19,4 | 87,6 |
| 19,5 | -29,3 |
| 19,5 | 11,1 |
| 19,5 | -14,2 |
| 19,5 | 49,9 |
| 19,5 | 4,8 |
| 19,6 | 34,5 |
| 19,6 | -35,0 |
| 19,6 | -19,0 |
| 19,6 | 41,6 |
| 19,6 | 28,0 |
| 19,7 | -8,0 |
| 19,7 | -23,0 |
| 19,7 | -9,3 |
| 19,7 | 28,0 |
| 19,7 | 13,4 |
| 19,8 | 24,9 |
| 19,8 | -30,1 |
| 19,8 | -20,4 |
| 19,8 | 5,2 |
| 19,8 | 25,3 |
| 19,9 | 3,5 |
| 19,9 | 18,5 |
| 19,9 | 37,2 |
| 19,9 | 10,6 |
| 19,9 | -27,2 |
| 20,0 | 21,0 |
| 20,0 | 25,4 |
| 20,0 | 9,3 |
| 20,0 | 6,3 |
| 20,0 | 17,1 |
| 20,1 | 16,5 |
| 20,1 | 18,2 |
| 20,1 | 48,1 |
| 20,1 | 57,3 |
| 20,1 | 71,3 |
| 20,2 | 78,5 |
| 20,2 | 69,1 |
| 20,2 | 57,1 |
| 20,2 | 74,4 |
| 20,2 | 52,3 |
| 20,3 | 28,7 |
| 20,3 | 16,4 |
| 20,3 | 39,7 |
| 20,3 | 115,0 |
| 20,3 | 120,3 |
| 20,4 | 44,4 |
| 20,4 | 21,1 |
| 20,4 | 21,7 |
| 20,4 | 56,0 |
| 20,4 | 84,8 |
| 20,5 | 47,4 |
| 20,5 | -8,2 |
| 20,5 | 14,7 |
| 20,5 | -15,9 |
| 20,5 | 41,4 |
| 20,6 | 46,0 |
| 20,6 | -5,2 |
| 20,6 | 36,8 |
| 20,6 | 17,3 |
| 20,6 | 4,2 |
| 20,7 | 89,4 |
| 20,7 | 40,8 |
| 20,7 | 3,4 |
| 20,7 | -2,2 |
| 20,7 | 3,1 |
| 20,8 | 37,4 |
| 20,8 | 87,9 |
| 20,8 | 17,5 |
| 20,8 | 42,1 |
| 20,8 | 27,6 |
| 20,9 | 36,6 |
| 20,9 | 43,4 |
| 20,9 | 55,7 |
| 20,9 | 30,5 |
| 20,9 | 11,7 |
| 21,0 | -12,3 |
| 21,0 | 21,7 |
| 21,0 | 41,1 |
| 21,0 | 38,0 |
| 21,0 | 53,3 |
| 21,1 | 34,2 |
| 21,1 | 29,9 |
| 21,1 | 54,4 |
| 21,1 | 101,5 |
| 21,1 | 68,2 |
| 21,2 | 21,1 |
| 21,2 | 38,1 |
| 21,2 | 83,9 |
| 21,2 | 102,6 |
| 21,2 | 17,4 |
| 21,3 | -4,5 |
| 21,3 | 26,0 |
| 21,3 | 68,6 |
| 21,3 | 67,9 |
| 21,3 | 58,2 |
| 21,4 | 41,9 |
| 21,4 | 48,9 |
| 21,4 | 62,1 |
| 21,4 | 100,0 |
| 21,4 | 84,7 |
| 21,5 | 12,5 |
| 21,5 | 54,9 |
| 21,5 | 83,8 |
| 21,5 | 88,9 |
| 21,5 | 69,5 |
| 21,6 | 48,4 |
| 21,6 | 98,5 |
| 21,6 | 137,8 |
| 21,6 | 83,4 |
| 21,6 | 64,2 |
| 21,7 | 100,1 |
| 21,7 | 81,6 |
| 21,7 | 56,1 |
| 21,7 | 91,3 |
| 21,7 | 99,6 |
| 21,8 | 98,2 |
| 21,8 | 61,0 |
| 21,8 | 0,1 |
| 21,8 | 31,7 |
| 21,8 | 151,4 |
| 21,9 | 170,8 |
| 21,9 | 180,0 |
| 21,9 | 104,3 |
| 21,9 | 21,7 |
| 21,9 | 98,7 |
| 22,0 | 97,5 |
| 22,0 | 124,3 |
| 22,0 | 164,2 |
| 22,0 | 129,0 |
| 22,0 | 105,5 |
| 22,1 | 84,9 |
| 22,1 | 106,4 |
| 22,1 | 93,7 |
| 22,1 | 156,3 |
| 22,1 | 99,8 |
| 22,2 | 104,5 |
| 22,2 | 87,2 |
| 22,2 | 161,0 |
| 22,2 | 137,5 |
| 22,2 | 190,2 |
| 22,3 | 120,2 |
| 22,3 | 112,3 |
| 22,3 | 134,6 |
| 22,3 | 126,9 |
| 22,3 | 151,9 |
| 22,4 | 124,3 |
| 22,4 | 183,8 |
| 22,4 | 172,1 |
| 22,4 | 166,5 |
| 22,4 | 140,2 |
| 22,5 | 160,8 |
| 22,5 | 152,1 |
| 22,5 | 174,2 |
| 22,5 | 162,8 |
| 22,5 | 172,4 |
| 22,6 | 207,0 |
| 22,6 | 208,0 |
| 22,6 | 248,6 |
| 22,6 | 229,3 |
| 22,6 | 215,0 |
| 22,7 | 223,5 |
| 22,7 | 247,7 |
| 22,7 | 265,6 |
| 22,7 | 230,1 |
| 22,7 | 224,5 |
| 22,8 | 259,1 |
| 22,8 | 295,0 |
| 22,8 | 265,4 |
| 22,8 | 284,2 |
| 22,8 | 305,5 |
| 22,9 | 327,4 |
| 22,9 | 356,3 |
| 22,9 | 280,0 |
| 22,9 | 298,6 |
| 22,9 | 322,8 |
| 23,0 | 393,1 |
| 23,0 | 337,5 |
| 23,0 | 353,6 |
| 23,0 | 311,9 |
| 23,0 | 334,9 |
| 23,1 | 349,9 |
| 23,1 | 364,7 |
| 23,1 | 411,6 |
| 23,1 | 400,2 |
| 23,1 | 384,3 |
| 23,2 | 308,5 |
| 23,2 | 382,2 |
| 23,2 | 375,1 |
| 23,2 | 395,4 |
| 23,2 | 374,0 |
| 23,3 | 338,9 |
| 23,3 | 375,3 |
| 23,3 | 333,1 |
| 23,3 | 331,0 |
| 23,3 | 230,5 |
| 23,4 | 297,4 |
| 23,4 | 317,5 |
| 23,4 | 350,9 |
| 23,4 | 268,1 |
| 23,4 | 223,5 |
| 23,5 | 255,2 |
| 23,5 | 276,5 |
| 23,5 | 297,2 |
| 23,5 | 245,5 |
| 23,5 | 269,7 |
| 23,6 | 183,4 |
| 23,6 | 205,7 |
| 23,6 | 152,1 |
| 23,6 | 151,7 |
| 23,6 | 156,8 |
| 23,7 | 159,6 |
| 23,7 | 191,1 |
| 23,7 | 138,6 |
| 23,7 | 122,0 |
| 23,7 | 108,5 |
| 23,8 | 129,9 |
| 23,8 | 131,8 |
| 23,8 | 86,7 |
| 23,8 | 83,3 |
| 23,8 | 47,6 |
| 23,9 | 63,9 |
| 23,9 | 87,1 |
| 23,9 | 106,5 |
| 23,9 | 107,7 |
| 23,9 | 78,2 |
| 24,0 | 54,8 |
| 24,0 | 37,4 |
| 24,0 | 48,4 |
| 24,0 | 42,2 |
| 24,0 | 22,7 |
| 24,1 | 48,6 |
| 24,1 | 68,6 |
| 24,1 | 53,2 |
| 24,1 | 46,1 |
| 24,1 | 15,5 |
| 24,2 | 10,8 |
| 24,2 | 60,7 |
| 24,2 | 72,9 |
| 24,2 | 77,1 |
| 24,2 | 39,6 |
| 24,3 | 12,0 |
| 24,3 | -1,3 |
| 24,3 | 17,0 |
| 24,3 | -5,5 |
| 24,3 | -3,2 |
| 24,4 | 9,5 |
| 24,4 | 15,2 |
| 24,4 | 12,0 |
| 24,4 | -14,1 |
| 24,4 | 58,5 |
| 24,5 | 34,4 |
| 24,5 | 15,9 |
| 24,5 | -4,0 |
| 24,5 | 2,5 |
| 24,5 | -22,0 |
| 24,6 | -11,0 |
| 24,6 | 9,2 |
| 24,6 | 9,9 |
| 24,6 | -9,2 |
| 24,6 | -21,0 |
| 24,7 | -22,0 |
| 24,7 | 5,7 |
| 24,7 | -0,3 |
| 24,7 | -1,7 |
| 24,7 | -9,0 |
| 24,8 | -2,4 |
| 24,8 | -24,0 |
| 24,8 | -25,0 |
| 24,8 | -11,2 |
| 24,8 | -12,0 |
| 24,9 | 4,0 |
| 24,9 | 5,0 |
| 24,9 | 2,1 |
| 24,9 | -19,0 |
| 24,9 | -24,0 |
| 25,0 | -24,0 |
| 25,0 | -3,0 |
| 25,0 | -16,0 |
| 25,0 | -26,0 |
| 25,0 | -33,0 |
| 25,1 | -30,0 |
| 25,1 | -8,2 |
| 25,1 | -12,0 |
| 25,1 | -7,7 |
| 25,1 | -10,7 |
| 25,2 | -38,0 |
| 25,2 | -52,0 |
| 25,2 | -51,0 |
| 25,2 | -32,0 |
| 25,2 | -11,3 |
| 25,3 | 0,3 |
| 25,3 | -16,0 |
| 25,3 | -20,0 |
| 25,3 | -10,9 |
| 25,3 | -3,0 |
| 25,4 | 5,9 |
| 25,4 | 9,6 |
| 25,4 | -2,4 |
| 25,4 | 5,0 |
| 25,4 | 7,5 |
| 25,5 | -5,1 |
| 25,5 | 9,8 |
| 25,5 | -10,0 |
| 25,5 | -18,0 |
| 25,5 | 9,7 |
| 25,6 | -6,0 |
| 25,6 | 14,1 |
| 25,6 | 6,2 |
| 25,6 | 8,5 |
| 25,6 | -1,9 |
| 25,7 | -10,0 |
| 25,7 | -10,0 |
| 25,7 | 25,3 |
| 25,7 | -12,7 |
| 25,7 | -8,5 |
| 25,8 | -17,0 |
| 25,8 | -2,8 |
| 25,8 | 15,5 |
| 25,8 | 10,1 |
| 25,8 | 13,0 |
| 25,9 | 36,5 |
| 25,9 | -6,5 |
| 25,9 | -11,0 |
| 25,9 | -6,3 |
| 25,9 | 2,6 |
| 26,0 | 16,2 |
| 26,0 | -14,0 |
| 26,0 | -9,0 |
| 26,0 | 26,3 |
| 26,0 | 8,7 |
| 26,1 | -13,8 |
| 26,1 | -14,0 |
| 26,1 | 17,4 |
| 26,1 | 7,9 |
| 26,1 | -13,2 |
| 26,2 | -10,0 |
| 26,2 | 4,7 |
| 26,2 | 6,6 |
| 26,2 | 28,4 |
| 26,2 | 25,3 |
| 26,3 | 42,9 |
| 26,3 | 35,7 |
| 26,3 | 9,8 |
| 26,3 | 10,5 |
| 26,3 | 21,7 |
| 26,4 | -6,0 |
| 26,4 | -4,0 |
| 26,4 | 33,6 |
| 26,4 | 35,3 |
| 26,4 | 62,6 |
| 26,5 | 56,8 |
| 26,5 | 16,4 |
| 26,5 | -1,1 |
| 26,5 | -2,1 |
| 26,5 | 32,1 |
| 26,6 | 68,3 |
| 26,6 | 17,6 |
| 26,6 | -8,0 |
| 26,6 | 21,9 |
| 26,6 | 36,0 |
| 26,7 | 46,6 |
| 26,7 | 65,7 |
| 26,7 | 49,2 |
| 26,7 | 28,0 |
| 26,7 | 34,2 |
| 26,8 | 35,4 |
| 26,8 | 45,8 |
| 26,8 | 35,9 |
| 26,8 | 46,5 |
| 26,8 | 63,3 |
| 26,9 | 68,7 |
| 26,9 | 51,0 |
| 26,9 | 46,7 |
| 26,9 | 31,0 |
| 26,9 | 41,4 |
| 27,0 | 22,1 |
| 27,0 | 42,3 |
| 27,0 | 45,2 |
| 27,0 | 29,3 |
| 27,0 | 116,7 |
| 27,1 | 48,5 |
| 27,1 | 71,9 |
| 27,1 | 68,1 |
| 27,1 | 52,9 |
| 27,1 | 106,5 |
| 27,2 | 112,2 |
| 27,2 | 70,1 |
| 27,2 | 94,9 |
| 27,2 | 100,8 |
| 27,2 | 113,4 |
| 27,3 | 121,1 |
| 27,3 | 87,4 |
| 27,3 | 72,5 |
| 27,3 | 95,7 |
| 27,3 | 83,6 |
| 27,4 | 143,5 |
| 27,4 | 114,6 |
| 27,4 | 108,3 |
| 27,4 | 67,7 |
| 27,4 | 47,6 |
| 27,5 | 97,4 |
| 27,5 | 96,4 |
| 27,5 | 155,1 |
| 27,5 | 121,0 |
| 27,5 | 96,7 |
| 27,6 | 95,7 |
| 27,6 | 87,6 |
| 27,6 | 117,9 |
| 27,6 | 108,0 |
| 27,6 | 80,5 |
| 27,7 | 74,0 |
| 27,7 | 118,8 |
| 27,7 | 118,6 |
| 27,7 | 134,5 |
| 27,7 | 149,2 |
| 27,8 | 165,6 |
| 27,8 | 117,0 |
| 27,8 | 104,2 |
| 27,8 | 119,9 |
| 27,8 | 116,9 |
| 27,9 | 128,6 |
| 27,9 | 118,9 |
| 27,9 | 94,5 |
| 27,9 | 98,0 |
| 27,9 | 105,1 |
| 28,0 | 128,5 |
| 28,0 | 125,5 |
| 28,0 | 119,5 |
| 28,0 | 114,3 |
| 28,0 | 88,4 |
| 28,1 | 89,5 |
| 28,1 | 81,6 |
| 28,1 | 105,1 |
| 28,1 | 104,4 |
| 28,1 | 156,1 |
| 28,2 | 138,7 |
| 28,2 | 147,8 |
| 28,2 | 118,4 |
| 28,2 | 92,2 |
| 28,2 | 92,1 |
| 28,3 | 77,6 |
| 28,3 | 101,0 |
| 28,3 | 113,3 |
| 28,3 | 129,5 |
| 28,3 | 118,5 |
| 28,4 | 97,2 |
| 28,4 | 91,5 |
| 28,4 | 102,8 |
| 28,4 | 69,6 |
| 28,4 | 96,8 |
| 28,5 | 109,6 |
| 28,5 | 98,3 |
| 28,5 | 109,9 |
| 28,5 | 95,2 |
| 28,5 | 108,3 |
| 28,6 | 75,7 |
| 28,6 | 80,1 |
| 28,6 | 96,6 |
| 28,6 | 58,2 |
| 28,6 | 100,8 |
| 28,7 | 112,1 |
| 28,7 | 77,3 |
| 28,7 | 81,7 |
| 28,7 | 131,9 |
| 28,7 | 86,1 |
| 28,8 | 78,8 |
| 28,8 | 71,2 |
| 28,8 | 70,8 |
| 28,8 | 78,7 |
| 28,8 | 66,1 |
| 28,9 | 104,0 |
| 28,9 | 64,2 |
| 28,9 | 75,6 |
| 28,9 | 65,1 |
| 28,9 | 84,0 |
| 29,0 | 90,4 |
| 29,0 | 41,8 |
| 29,0 | 43,8 |
| 29,0 | 59,1 |
| 29,0 | 73,0 |
| 29,1 | 91,7 |
| 29,1 | 89,6 |
| 29,1 | 26,2 |
| 29,1 | 20,7 |
| 29,1 | 16,6 |
| 29,2 | 59,5 |
| 29,2 | 87,0 |
| 29,2 | 43,6 |
| 29,2 | 35,6 |
| 29,2 | 18,7 |
| 29,3 | 27,2 |
| 29,3 | 42,4 |
| 29,3 | 46,7 |
| 29,3 | 90,3 |
| 29,3 | 82,4 |
| 29,4 | 61,8 |
| 29,4 | 53,4 |
| 29,4 | -2,7 |
| 29,4 | 0,8 |
| 29,4 | 23,7 |
| 29,5 | 16,0 |
| 29,5 | 72,6 |
| 29,5 | 69,2 |
| 29,5 | 39,6 |
| 29,5 | -6,9 |
| 29,6 | -20,0 |
| 29,6 | 1,1 |
| 29,6 | 35,1 |
| 29,6 | 44,8 |
| 29,6 | 28,1 |
| 29,7 | 44,4 |
| 29,7 | 41,5 |
| 29,7 | 1,3 |
| 29,7 | 16,5 |
| 29,7 | 33,9 |
| 29,8 | 14,9 |
| 29,8 | 22,3 |
| 29,8 | 34,6 |
| 29,8 | 9,4 |
| 29,8 | 62,9 |
| 29,9 | 34,5 |
| 29,9 | 36,3 |
| 29,9 | 4,7 |
| 29,9 | -17,0 |
| 29,9 | 7,9 |
| 30,0 | -18,2 |
| 30,0 | 34,4 |
| 30,0 | 42,7 |
| 30,0 | 22,0 |
| 30,0 | -10,7 |
| 30,1 | -16,2 |
| 30,1 | 14,3 |
| 30,1 | -4,0 |
| 30,1 | 21,7 |
| 30,1 | -4,5 |
| 30,2 | 28,1 |
| 30,2 | 2,3 |
| 30,2 | -5,6 |
| 30,2 | 24,3 |
| 30,2 | 36,7 |
| 30,3 | 31,3 |
| 30,3 | 9,0 |
| 30,3 | -6,1 |
| 30,3 | -22,0 |
| 30,3 | -17,0 |
| 30,4 | 10,6 |
| 30,4 | 10,7 |
| 30,4 | 6,6 |
| 30,4 | 27,5 |
| 30,4 | 2,3 |
| 30,5 | -10,0 |
| 30,5 | -5,7 |
| 30,5 | -13,6 |
| 30,5 | 22,5 |
| 30,5 | 39,7 |
| 30,6 | 14,4 |
| 30,6 | 1,0 |
| 30,6 | 4,7 |
| 30,6 | 8,0 |
| 30,6 | 16,3 |
| 30,7 | -5,0 |
| 30,7 | -9,5 |
| 30,7 | -1,0 |
| 30,7 | 33,7 |
| 30,7 | 16,7 |
| 30,8 | -4,4 |
| 30,8 | 7,1 |
| 30,8 | -1,5 |
| 30,8 | 31,1 |
| 30,8 | 27,5 |
| 30,9 | 24,6 |
| 30,9 | 14,9 |
| 30,9 | 46,7 |
| 30,9 | 33,7 |
| 30,9 | -7,1 |
| 31,0 | -28,0 |
| 31,0 | -35,0 |
| 31,0 | -23,0 |
| 31,0 | 8,1 |
| 31,0 | 5,2 |
| 31,1 | 2,1 |
| 31,1 | -0,7 |
| 31,1 | 9,1 |
| 31,1 | 20,3 |
| 31,1 | 21,5 |
| 31,2 | 14,8 |
| 31,2 | -13,4 |
| 31,2 | 40,4 |
| 31,2 | -2,0 |
| 31,2 | 3,0 |
| 31,3 | -13,0 |
| 31,3 | -9,4 |
| 31,3 | 28,4 |
| 31,3 | 2,5 |
| 31,3 | 8,7 |
| 31,4 | 25,7 |
| 31,4 | 10,1 |
| 31,4 | 19,4 |
| 31,4 | 6,5 |
| 31,4 | 17,4 |
| 31,5 | 42,4 |
| 31,5 | 10,6 |
| 31,5 | 25,1 |
| 31,5 | 42,1 |
| 31,5 | 77,8 |
| 31,6 | 126,2 |
| 31,6 | 170,8 |
| 31,6 | 211,3 |
| 31,6 | 177,0 |
| 31,6 | 153,0 |
| 31,7 | 68,8 |
| 31,7 | 4,3 |
| 31,7 | 19,8 |
| 31,7 | 22,8 |
| 31,7 | 23,4 |
| 31,8 | 29,0 |
| 31,8 | 1,8 |
| 31,8 | 3,0 |
| 31,8 | 14,2 |
| 31,8 | 37,2 |
| 31,9 | 19,2 |
| 31,9 | 11,1 |
| 31,9 | 27,3 |
| 31,9 | -10,0 |
| 31,9 | -21,0 |
| 32,0 | -24,0 |
| 32,0 | -1,1 |
| 32,0 | 1,4 |
| 32,0 | -1,5 |
| 32,0 | -2,0 |
| 32,1 | 1,0 |
| 32,1 | 9,7 |
| 32,1 | -2,0 |
| 32,1 | 7,7 |
| 32,1 | 4,4 |
| 32,2 | 8,6 |
| 32,2 | 15,3 |
| 32,2 | 24,1 |
| 32,2 | -10,2 |
| 32,2 | -11,7 |
| 32,3 | -9,0 |
| 32,3 | -4,9 |
| 32,3 | 22,4 |
| 32,3 | 5,0 |
| 32,3 | 3,1 |
| 32,4 | 11,9 |
| 32,4 | 3,0 |
| 32,4 | 16,8 |
| 32,4 | 0,1 |
| 32,4 | -9,9 |
| 32,5 | -8,0 |
| 32,5 | -15,0 |
| 32,5 | 10,3 |
| 32,5 | 5,8 |
| 32,5 | 30,1 |
| 32,6 | -7,7 |
| 32,6 | -13,0 |
| 32,6 | -12,0 |
| 32,6 | 19,2 |
| 32,6 | 17,5 |
| 32,7 | -2,2 |
| 32,7 | 4,9 |
| 32,7 | -18,0 |
| 32,7 | -21,0 |
| 32,7 | -6,3 |
| 32,8 | -12,0 |
| 32,8 | -9,8 |
| 32,8 | -18,0 |
| 32,8 | -24,0 |
| 32,8 | -28,0 |
| 32,9 | -16,0 |
| 32,9 | -0,5 |
| 32,9 | 18,1 |
| 32,9 | -6,9 |
| 32,9 | -8,0 |
| 33,0 | -5,0 |
| 33,0 | 20,9 |
| 33,0 | -1,7 |
| 33,0 | -2,0 |
| 33,0 | 5,7 |
| 33,1 | 2,8 |
| 33,1 | -5,0 |
| 33,1 | -15,0 |
| 33,1 | -19,0 |
| 33,1 | -21,0 |
| 33,2 | -33,0 |
| 33,2 | -43,0 |
| 33,2 | -42,0 |
| 33,2 | -29,0 |
| 33,2 | -0,7 |
| 33,3 | 12,6 |
| 33,3 | 1,2 |
| 33,3 | -8,1 |
| 33,3 | 31,3 |
| 33,3 | -2,1 |
| 33,4 | 5,8 |
| 33,4 | -23,0 |
| 33,4 | -21,0 |
| 33,4 | -0,1 |
| 33,4 | 2,8 |
| 33,5 | 13,0 |
| 33,5 | 27,0 |
| 33,5 | 11,3 |
| 33,5 | -10,0 |
| 33,5 | -9,0 |
| 33,6 | 13,9 |
| 33,6 | 21,0 |
| 33,6 | -6,6 |
| 33,6 | 1,0 |
| 33,6 | 6,0 |
| 33,7 | 20,1 |
| 33,7 | -6,0 |
| 33,7 | 29,7 |
| 33,7 | 11,0 |
| 33,7 | 28,1 |
| 33,8 | 12,8 |
| 33,8 | 16,8 |
| 33,8 | 45,2 |
| 33,8 | 57,9 |
| 33,8 | 38,3 |
| 33,9 | 24,4 |
| 33,9 | 8,0 |
| 33,9 | 9,2 |
| 33,9 | -1,3 |
| 33,9 | 20,4 |
| 34,0 | 55,9 |
| 34,0 | 45,9 |
| 34,0 | 75,0 |
| 34,0 | 89,9 |
| 34,0 | 75,6 |
| 34,1 | 74,0 |
| 34,1 | 37,1 |
| 34,1 | 101,7 |
| 34,1 | 101,3 |
| 34,1 | 129,1 |
| 34,2 | 110,3 |
| 34,2 | 130,0 |
| 34,2 | 146,1 |
| 34,2 | 151,3 |
| 34,2 | 157,2 |
| 34,3 | 155,1 |
| 34,3 | 135,9 |
| 34,3 | 173,3 |
| 34,3 | 163,2 |
| 34,3 | 181,2 |
| 34,4 | 213,6 |
| 34,4 | 197,4 |
| 34,4 | 213,2 |
| 34,4 | 203,4 |
| 34,4 | 211,2 |
| 34,5 | 242,1 |
| 34,5 | 254,4 |
| 34,5 | 280,5 |
| 34,5 | 292,5 |
| 34,5 | 276,2 |
| 34,6 | 244,4 |
| 34,6 | 242,6 |
| 34,6 | 271,4 |
| 34,6 | 286,5 |
| 34,6 | 326,7 |
| 34,7 | 364,1 |
| 34,7 | 398,1 |
| 34,7 | 367,2 |
| 34,7 | 342,3 |
| 34,7 | 321,0 |
| 34,8 | 293,8 |
| 34,8 | 317,0 |
| 34,8 | 316,3 |
| 34,8 | 395,2 |
| 34,8 | 461,6 |
| 34,9 | 398,8 |
| 34,9 | 366,5 |
| 34,9 | 372,6 |
| 34,9 | 312,8 |
| 34,9 | 304,5 |
| 35,0 | 373,9 |
| 35,0 | 319,8 |
| 35,0 | 357,8 |
| 35,0 | 407,4 |
| 35,0 | 326,8 |
| 35,1 | 273,2 |
| 35,1 | 302,0 |
| 35,1 | 252,2 |
| 35,1 | 280,9 |
| 35,1 | 219,3 |
| 35,2 | 175,0 |
| 35,2 | 232,7 |
| 35,2 | 254,8 |
| 35,2 | 217,0 |
| 35,2 | 229,6 |
| 35,3 | 205,8 |
| 35,3 | 190,0 |
| 35,3 | 166,8 |
| 35,3 | 196,4 |
| 35,3 | 127,3 |
| 35,4 | 178,4 |
| 35,4 | 160,5 |
| 35,4 | 164,4 |
| 35,4 | 147,5 |
| 35,4 | 138,9 |
| 35,5 | 167,9 |
| 35,5 | 144,0 |
| 35,5 | 102,3 |
| 35,5 | 139,7 |
| 35,5 | 94,6 |
| 35,6 | 85,9 |
| 35,6 | 119,2 |
| 35,6 | 123,8 |
| 35,6 | 104,8 |
| 35,6 | 141,0 |
| 35,7 | 129,7 |
| 35,7 | 59,0 |
| 35,7 | 52,4 |
| 35,7 | 99,6 |
| 35,7 | 79,8 |
| 35,8 | 78,4 |
| 35,8 | 82,4 |
| 35,8 | 74,9 |
| 35,8 | 70,3 |
| 35,8 | 76,1 |
| 35,9 | 93,9 |
| 35,9 | 99,0 |
| 35,9 | 64,6 |
| 35,9 | 57,3 |
| 35,9 | 56,7 |
| 36,0 | 48,8 |
| 36,0 | 58,3 |
| 36,0 | 65,3 |
| 36,0 | 54,6 |
| 36,0 | 66,0 |
| 36,1 | 109,0 |
| 36,1 | 127,9 |
| 36,1 | 53,5 |
| 36,1 | 50,3 |
| 36,1 | 54,0 |
| 36,2 | 53,9 |
| 36,2 | 50,1 |
| 36,2 | 37,5 |
| 36,2 | 80,4 |
| 36,2 | 52,0 |
| 36,3 | 74,7 |
| 36,3 | 71,7 |
| 36,3 | 50,4 |
| 36,3 | 7,1 |
| 36,3 | 66,0 |
| 36,4 | 35,7 |
| 36,4 | 71,5 |
| 36,4 | 63,4 |
| 36,4 | 83,8 |
| 36,4 | 74,0 |
| 36,5 | 54,4 |
| 36,5 | 59,7 |
| 36,5 | 64,3 |
| 36,5 | 88,7 |
| 36,5 | 66,7 |
| 36,6 | 53,8 |
| 36,6 | 82,1 |
| 36,6 | 109,9 |
| 36,6 | 95,1 |
| 36,6 | 113,9 |
| 36,7 | 107,6 |
| 36,7 | 81,0 |
| 36,7 | 43,2 |
| 36,7 | 54,7 |
| 36,7 | 95,1 |
| 36,8 | 82,4 |
| 36,8 | 90,8 |
| 36,8 | 80,8 |
| 36,8 | 69,7 |
| 36,8 | 51,0 |
| 36,9 | 54,3 |
| 36,9 | 73,2 |
| 36,9 | 90,7 |
| 36,9 | 97,0 |
| 36,9 | 118,7 |
| 37,0 | 134,0 |
| 37,0 | 96,8 |
| 37,0 | 65,8 |
| 37,0 | 71,8 |
| 37,0 | 61,8 |
| 37,1 | 74,1 |
| 37,1 | 130,0 |
| 37,1 | 146,5 |
| 37,1 | 119,8 |
| 37,1 | 128,9 |
| 37,2 | 83,7 |
| 37,2 | 53,9 |
| 37,2 | 79,9 |
| 37,2 | 86,9 |
| 37,2 | 85,4 |
| 37,3 | 172,1 |
| 37,3 | 143,6 |
| 37,3 | 152,4 |
| 37,3 | 135,9 |
| 37,3 | 119,8 |
| 37,4 | 60,7 |
| 37,4 | 107,8 |
| 37,4 | 140,2 |
| 37,4 | 154,3 |
| 37,4 | 123,7 |
| 37,5 | 145,0 |
| 37,5 | 205,0 |
| 37,5 | 137,7 |
| 37,5 | 105,7 |
| 37,5 | 143,6 |
| 37,6 | 123,3 |
| 37,6 | 154,7 |
| 37,6 | 176,5 |
| 37,6 | 225,5 |
| 37,6 | 180,9 |
| 37,7 | 135,2 |
| 37,7 | 175,4 |
| 37,7 | 148,3 |
| 37,7 | 114,9 |
| 37,7 | 139,0 |
| 37,8 | 186,1 |
| 37,8 | 196,0 |
| 37,8 | 205,5 |
| 37,8 | 191,9 |
| 37,8 | 196,2 |
| 37,9 | 162,8 |
| 37,9 | 88,7 |
| 37,9 | 158,5 |
| 37,9 | 135,7 |
| 37,9 | 182,8 |
| 38,0 | 191,3 |
| 38,0 | 212,5 |
| 38,0 | 226,5 |
| 38,0 | 217,7 |
| 38,0 | 191,2 |
| 38,1 | 155,3 |
| 38,1 | 191,2 |
| 38,1 | 201,0 |
| 38,1 | 204,8 |
| 38,1 | 171,1 |
| 38,2 | 169,8 |
| 38,2 | 190,5 |
| 38,2 | 192,1 |
| 38,2 | 217,3 |
| 38,2 | 191,8 |
| 38,3 | 203,0 |
| 38,3 | 225,9 |
| 38,3 | 226,8 |
| 38,3 | 209,1 |
| 38,3 | 196,8 |
| 38,4 | 220,3 |
| 38,4 | 211,2 |
| 38,4 | 189,6 |
| 38,4 | 197,2 |
| 38,4 | 211,7 |
| 38,5 | 181,3 |
| 38,5 | 223,6 |
| 38,5 | 182,5 |
| 38,5 | 226,7 |
| 38,5 | 207,5 |
| 38,6 | 230,3 |
| 38,6 | 244,1 |
| 38,6 | 232,6 |
| 38,6 | 214,8 |
| 38,6 | 197,7 |
| 38,7 | 176,5 |
| 38,7 | 203,1 |
| 38,7 | 152,7 |
| 38,7 | 207,1 |
| 38,7 | 176,6 |
| 38,8 | 205,6 |
| 38,8 | 158,7 |
| 38,8 | 217,8 |
| 38,8 | 208,8 |
| 38,8 | 250,8 |
| 38,9 | 210,9 |
| 38,9 | 203,9 |
| 38,9 | 233,6 |
| 38,9 | 245,7 |
| 38,9 | 228,8 |
| 39,0 | 195,8 |
| 39,0 | 273,3 |
| 39,0 | 158,0 |
| 39,0 | 191,0 |
| 39,0 | 195,6 |
| 39,1 | 237,2 |
| 39,1 | 197,2 |
| 39,1 | 279,2 |
| 39,1 | 184,7 |
| 39,1 | 238,4 |
| 39,2 | 192,3 |
| 39,2 | 145,0 |
| 39,2 | 213,1 |
| 39,2 | 261,8 |
| 39,2 | 214,3 |
| 39,3 | 233,2 |
| 39,3 | 245,6 |
| 39,3 | 214,5 |
| 39,3 | 199,4 |
| 39,3 | 226,5 |
| 39,4 | 195,9 |
| 39,4 | 178,7 |
| 39,4 | 202,2 |
| 39,4 | 208,3 |
| 39,4 | 210,6 |
| 39,5 | 228,3 |
| 39,5 | 230,3 |
| 39,5 | 216,1 |
| 39,5 | 232,5 |
| 39,5 | 211,7 |
| 39,6 | 202,5 |
| 39,6 | 178,5 |
| 39,6 | 182,0 |
| 39,6 | 192,0 |
| 39,6 | 201,7 |
| 39,7 | 162,5 |
| 39,7 | 162,1 |
| 39,7 | 155,7 |
| 39,7 | 145,3 |
| 39,7 | 129,3 |
| 39,8 | 158,3 |
| 39,8 | 144,5 |
| 39,8 | 149,4 |
| 39,8 | 168,6 |
| 39,8 | 177,9 |
| 39,9 | 140,5 |
| 39,9 | 150,5 |
| 39,9 | 100,7 |
| 39,9 | 104,7 |
| 39,9 | 113,9 |
| 40,0 | 106,6 |
| 40,0 | 87,0 |
| 40,0 | 107,7 |
| 40,0 | 139,7 |
| 40,0 | 116,8 |
| 40,1 | 141,4 |
| 40,1 | 116,3 |
| 40,1 | 106,7 |
| 40,1 | 58,2 |
| 40,1 | 95,5 |
| 40,2 | 56,6 |
| 40,2 | 65,5 |
| 40,2 | 61,4 |
| 40,2 | 64,6 |
| 40,2 | 119,3 |
| 40,3 | 112,2 |
| 40,3 | 44,5 |
| 40,3 | 109,9 |
| 40,3 | 102,7 |
| 40,3 | 62,1 |
| 40,4 | 105,7 |
| 40,4 | 112,5 |
| 40,4 | 82,2 |
| 40,4 | 54,3 |
| 40,4 | 73,7 |
| 40,5 | 39,8 |
| 40,5 | 71,3 |
| 40,5 | 63,5 |
| 40,5 | 56,4 |
| 40,5 | 76,0 |
| 40,6 | 88,1 |
| 40,6 | 103,8 |
| 40,6 | 62,4 |
| 40,6 | 67,9 |
| 40,6 | 62,4 |
| 40,7 | 54,1 |
| 40,7 | 20,7 |
| 40,7 | 24,1 |
| 40,7 | 40,8 |
| 40,7 | 29,2 |
| 40,8 | 17,1 |
| 40,8 | 4,8 |
| 40,8 | 31,8 |
| 40,8 | 60,5 |
| 40,8 | 28,0 |
| 40,9 | 23,1 |
| 40,9 | 38,1 |
| 40,9 | 42,0 |
| 40,9 | 35,6 |
| 40,9 | 31,2 |
| 41,0 | 28,5 |
| 41,0 | 9,8 |
| 41,0 | 3,0 |
| 41,0 | -1,8 |
| 41,0 | -0,5 |
| 41,1 | 8,0 |
| 41,1 | 20,3 |
| 41,1 | 25,7 |
| 41,1 | 5,0 |
| 41,1 | 4,0 |
| 41,2 | -3,5 |
| 41,2 | 18,3 |
| 41,2 | -5,0 |
| 41,2 | 5,0 |
| 41,2 | 39,8 |
| 41,3 | 31,7 |
| 41,3 | 5,0 |
| 41,3 | 4,0 |
| 41,3 | 41,1 |
| 41,3 | 20,2 |
| 41,4 | -9,0 |
| 41,4 | -29,0 |
| 41,4 | -26,0 |
| 41,4 | -16,0 |
| 41,4 | 2,0 |
| 41,5 | 10,0 |
| 41,5 | 36,1 |
| 41,5 | 7,8 |
| 41,5 | 1,0 |
| 41,5 | -11,0 |
| 41,6 | 2,4 |
| 41,6 | -6,0 |
| 41,6 | -18,0 |
| 41,6 | -15,0 |
| 41,6 | -18,0 |
| 41,7 | 16,7 |
| 41,7 | 6,0 |
| 41,7 | 41,3 |
| 41,7 | -9,0 |
| 41,7 | -15,0 |
| 41,8 | -31,0 |
| 41,8 | -23,0 |
| 41,8 | -19,0 |
| 41,8 | -14,0 |
| 41,8 | -15,0 |
| 41,9 | 4,7 |
| 41,9 | 2,0 |
| 41,9 | 13,2 |
| 41,9 | 47,0 |
| 41,9 | 11,0 |
| 42,0 | -2,0 |
| 42,0 | -9,0 |
| 42,0 | -9,0 |
| 42,0 | -15,0 |
| 42,0 | -13,0 |
| 42,1 | -5,0 |
| 42,1 | 14,3 |
| 42,1 | 4,9 |
| 42,1 | -5,0 |
| 42,1 | -11,0 |
| 42,2 | -7,0 |
| 42,2 | -3,0 |
| 42,2 | 6,5 |
| 42,2 | -2,0 |
| 42,2 | 2,9 |
| 42,3 | 3,0 |
| 42,3 | 4,6 |
| 42,3 | 38,5 |
| 42,3 | 0,0 |
| 42,3 | -11,0 |
| 42,4 | -10,0 |
| 42,4 | 3,3 |
| 42,4 | 19,5 |
| 42,4 | -6,0 |
| 42,4 | -26,0 |
| 42,5 | -23,0 |
| 42,5 | -7,0 |
| 42,5 | 39,7 |
| 42,5 | 5,0 |
| 42,5 | 11,0 |
| 42,6 | -14,0 |
| 42,6 | 4,0 |
| 42,6 | -27,0 |
| 42,6 | -16,0 |
| 42,6 | 17,7 |
| 42,7 | 23,7 |
| 42,7 | 15,3 |
| 42,7 | 3,0 |
| 42,7 | 2,0 |
| 42,7 | 30,7 |
| 42,8 | -10,0 |
| 42,8 | -27,0 |
| 42,8 | -41,0 |
| 42,8 | -16,0 |
| 42,8 | 5,4 |
| 42,9 | 52,9 |
| 42,9 | 18,0 |
| 42,9 | -1,0 |
| 42,9 | 42,2 |
| 42,9 | -18,0 |
| 43,0 | -14,0 |
| 43,0 | -20,0 |
| 43,0 | -3,0 |
| 43,0 | -12,0 |
| 43,0 | -22,0 |
| 43,1 | -21,0 |
| 43,1 | -19,0 |
| 43,1 | -12,0 |
| 43,1 | -12,0 |
| 43,1 | -6,0 |
| 43,2 | -4,0 |
| 43,2 | 25,2 |
| 43,2 | 12,0 |
| 43,2 | 26,7 |
| 43,2 | 27,7 |
| 43,3 | 43,0 |
| 43,3 | 13,3 |
| 43,3 | 2,0 |
| 43,3 | -3,0 |
| 43,3 | 1,0 |
| 43,4 | 14,0 |
| 43,4 | 7,0 |
| 43,4 | 9,0 |
| 43,4 | 18,3 |
| 43,4 | -2,0 |
| 43,5 | -9,0 |
| 43,5 | -8,0 |
| 43,5 | 13,5 |
| 43,5 | 23,2 |
| 43,5 | 13,0 |
| 43,6 | 11,0 |
| 43,6 | 16,6 |
| 43,6 | 22,5 |
| 43,6 | 7,0 |
| 43,6 | 8,0 |
| 43,7 | 21,0 |
| 43,7 | 21,4 |
| 43,7 | 1,0 |
| 43,7 | -4,0 |
| 43,7 | 2,0 |
| 43,8 | 21,3 |
| 43,8 | 25,7 |
| 43,8 | 29,9 |
| 43,8 | 43,7 |
| 43,8 | 62,2 |
| 43,9 | 14,0 |
| 43,9 | 0,0 |
| 43,9 | -6,0 |
| 43,9 | 30,9 |
| 43,9 | 12,7 |
| 44,0 | 20,1 |
| 44,0 | 26,0 |
| 44,0 | 37,7 |
| 44,0 | 24,0 |
| 44,0 | 28,9 |
| 44,1 | 22,2 |
| 44,1 | 23,9 |
| 44,1 | 60,7 |
| 44,1 | 28,0 |
| 44,1 | 31,3 |
| 44,2 | 4,0 |
| 44,2 | 10,0 |
| 44,2 | 13,0 |
| 44,2 | 19,7 |
| 44,2 | 30,2 |
| 44,3 | 18,0 |
| 44,3 | 29,9 |
| 44,3 | 42,5 |
| 44,3 | 38,0 |
| 44,3 | 19,0 |
| 44,4 | 18,0 |
| 44,4 | 22,3 |
| 44,4 | 39,1 |
| 44,4 | 16,0 |
| 44,4 | 27,4 |
| 44,5 | 59,6 |
| 44,5 | 53,3 |
| 44,5 | 59,7 |
| 44,5 | 37,6 |
| 44,5 | 38,7 |
| 44,6 | 21,4 |
| 44,6 | 27,3 |
| 44,6 | 33,5 |
| 44,6 | 13,0 |
| 44,6 | 13,0 |
| 44,7 | 32,9 |
| 44,7 | 30,9 |
| 44,7 | 31,7 |
| 44,7 | 43,5 |
| 44,7 | 69,6 |
| 44,8 | 32,6 |
| 44,8 | 32,1 |
| 44,8 | 61,9 |
| 44,8 | 27,5 |
| 44,8 | 24,5 |
| 44,9 | 22,4 |
| 44,9 | 29,4 |
| 44,9 | 37,6 |
| 44,9 | 41,7 |
| 44,9 | 56,5 |
| 45,0 | 25,8 |
| 45,0 | 17,4 |
| 45,0 | 32,3 |
| 45,0 | 47,3 |
| 45,0 | 72,7 |
| 45,1 | 45,4 |
| 45,1 | 118,5 |
| 45,1 | 101,4 |
| 45,1 | 31,3 |
| 45,1 | 65,8 |
| 45,2 | 58,6 |
| 45,2 | 30,1 |
| 45,2 | 49,1 |
| 45,2 | 56,3 |
| 45,2 | 95,6 |
| 45,3 | 137,2 |
| 45,3 | 118,1 |
| 45,3 | 166,9 |
| 45,3 | 142,6 |
| 45,3 | 167,8 |
| 45,4 | 120,8 |
| 45,4 | 96,2 |
| 45,4 | 163,6 |
| 45,4 | 48,9 |
| 45,4 | 121,1 |
| 45,5 | 68,1 |
| 45,5 | 74,9 |
| 45,5 | 71,2 |
| 45,5 | 62,0 |
| 45,5 | 91,1 |
| 45,6 | 100,8 |
| 45,6 | 87,8 |
| 45,6 | 93,4 |
| 45,6 | 34,9 |
| 45,6 | 107,7 |
| 45,7 | 87,1 |
| 45,7 | 72,4 |
| 45,7 | 84,9 |
| 45,7 | 72,8 |
| 45,7 | 112,5 |
| 45,8 | 52,4 |
| 45,8 | 76,2 |
| 45,8 | 106,0 |
| 45,8 | 98,2 |
| 45,8 | 108,9 |
| 45,9 | 84,3 |
| 45,9 | 94,8 |
| 45,9 | 105,7 |
| 45,9 | 96,7 |
| 45,9 | 100,8 |
| 46,0 | 94,4 |
| 46,0 | 105,0 |
| 46,0 | 107,7 |
| 46,0 | 100,8 |
| 46,0 | 90,8 |
| 46,1 | 81,8 |
| 46,1 | 68,1 |
| 46,1 | 92,0 |
| 46,1 | 123,5 |
| 46,1 | 119,8 |
| 46,2 | 141,8 |
| 46,2 | 125,3 |
| 46,2 | 108,2 |
| 46,2 | 111,4 |
| 46,2 | 112,4 |
| 46,3 | 108,4 |
| 46,3 | 122,4 |
| 46,3 | 145,4 |
| 46,3 | 149,6 |
| 46,3 | 94,0 |
| 46,4 | 125,5 |
| 46,4 | 176,9 |
| 46,4 | 89,7 |
| 46,4 | 138,5 |
| 46,4 | 169,9 |
| 46,5 | 131,5 |
| 46,5 | 149,5 |
| 46,5 | 130,3 |
| 46,5 | 193,6 |
| 46,5 | 181,1 |
| 46,6 | 161,1 |
| 46,6 | 141,1 |
| 46,6 | 157,7 |
| 46,6 | 202,2 |
| 46,6 | 135,6 |
| 46,7 | 120,3 |
| 46,7 | 228,6 |
| 46,7 | 154,1 |
| 46,7 | 114,6 |
| 46,7 | 148,6 |
| 46,8 | 176,3 |
| 46,8 | 213,8 |
| 46,8 | 128,0 |
| 46,8 | 172,9 |
| 46,8 | 171,1 |
| 46,9 | 194,3 |
| 46,9 | 163,6 |
| 46,9 | 145,5 |
| 46,9 | 149,4 |
| 46,9 | 173,5 |
| 47,0 | 150,4 |
| 47,0 | 129,8 |
| 47,0 | 188,9 |
| 47,0 | 178,2 |
| 47,0 | 169,8 |
| 47,1 | 193,8 |
| 47,1 | 209,9 |
| 47,1 | 179,3 |
| 47,1 | 141,2 |
| 47,1 | 159,9 |
| 47,2 | 152,7 |
| 47,2 | 153,4 |
| 47,2 | 162,9 |
| 47,2 | 144,6 |
| 47,2 | 143,3 |
| 47,3 | 165,6 |
| 47,3 | 179,3 |
| 47,3 | 128,2 |
| 47,3 | 131,5 |
| 47,3 | 153,7 |
| 47,4 | 141,1 |
| 47,4 | 145,6 |
| 47,4 | 148,0 |
| 47,4 | 171,9 |
| 47,4 | 175,8 |
| 47,5 | 167,5 |
| 47,5 | 151,3 |
| 47,5 | 141,1 |
| 47,5 | 150,2 |
| 47,5 | 131,2 |
| 47,6 | 128,5 |
| 47,6 | 144,5 |
| 47,6 | 123,7 |
| 47,6 | 104,5 |
| 47,6 | 127,9 |
| 47,7 | 103,2 |
| 47,7 | 176,0 |
| 47,7 | 135,6 |
| 47,7 | 99,5 |
| 47,7 | 155,9 |
| 47,8 | 142,8 |
| 47,8 | 174,5 |
| 47,8 | 126,6 |
| 47,8 | 82,4 |
| 47,8 | 110,8 |
| 47,9 | 154,2 |
| 47,9 | 76,9 |
| 47,9 | 143,3 |
| 47,9 | 142,7 |
| 47,9 | 135,4 |
| 48,0 | 183,8 |
| 48,0 | 147,1 |
| 48,0 | 127,0 |
| 48,0 | 125,9 |
| 48,0 | 134,8 |
| 48,1 | 135,3 |
| 48,1 | 91,4 |
| 48,1 | 130,6 |
| 48,1 | 116,6 |
| 48,1 | 111,9 |
| 48,2 | 112,8 |
| 48,2 | 151,2 |
| 48,2 | 118,3 |
| 48,2 | 132,1 |
| 48,2 | 135,4 |
| 48,3 | 157,8 |
| 48,3 | 153,7 |
| 48,3 | 129,3 |
| 48,3 | 156,4 |
| 48,3 | 157,9 |
| 48,4 | 185,9 |
| 48,4 | 108,9 |
| 48,4 | 116,5 |
| 48,4 | 128,2 |
| 48,4 | 134,4 |
| 48,5 | 131,1 |
| 48,5 | 121,8 |
| 48,5 | 149,5 |
| 48,5 | 168,4 |
| 48,5 | 162,9 |
| 48,6 | 134,9 |
| 48,6 | 150,1 |
| 48,6 | 157,0 |
| 48,6 | 153,3 |
| 48,6 | 141,1 |
| 48,7 | 127,7 |
| 48,7 | 114,2 |
| 48,7 | 149,9 |
| 48,7 | 169,1 |
| 48,7 | 167,1 |
| 48,8 | 171,4 |
| 48,8 | 147,8 |
| 48,8 | 154,7 |
| 48,8 | 145,1 |
| 48,8 | 147,3 |
| 48,9 | 161,4 |
| 48,9 | 145,3 |
| 48,9 | 110,1 |
| 48,9 | 172,2 |
| 48,9 | 170,3 |
| 49,0 | 164,9 |
| 49,0 | 215,6 |
| 49,0 | 171,6 |
| 49,0 | 131,6 |
| 49,0 | 120,2 |
| 49,1 | 126,7 |
| 49,1 | 136,9 |
| 49,1 | 124,7 |
| 49,1 | 120,2 |
| 49,1 | 156,4 |
| 49,2 | 111,5 |
| 49,2 | 154,5 |
| 49,2 | 138,0 |
| 49,2 | 104,3 |
| 49,2 | 164,5 |
| 49,3 | 150,4 |
| 49,3 | 158,3 |
| 49,3 | 54,5 |
| 49,3 | 96,8 |
| 49,3 | 74,4 |
| 49,4 | 103,8 |
| 49,4 | 71,7 |
| 49,4 | 54,2 |
| 49,4 | 112,0 |
| 49,4 | 83,3 |
| 49,5 | 57,1 |
| 49,5 | 86,6 |
| 49,5 | 95,0 |
| 49,5 | 78,5 |
| 49,5 | 95,1 |
| 49,6 | 79,8 |
| 49,6 | 86,2 |
| 49,6 | 45,3 |
| 49,6 | 61,8 |
| 49,6 | 47,8 |
| 49,7 | 19,3 |
| 49,7 | 55,9 |
| 49,7 | 53,8 |
| 49,7 | 65,2 |
| 49,7 | 70,8 |
| 49,8 | 81,1 |
| 49,8 | 64,2 |
| 49,8 | 35,1 |
| 49,8 | 38,7 |
| 49,8 | 67,2 |
| 49,9 | 47,7 |
| 49,9 | 11,3 |
| 49,9 | 16,2 |
| 49,9 | 42,3 |
| 49,9 | 42,1 |
| 50,0 | 38,7 |
| 50,0 | 22,0 |
| 50,0 | 19,6 |
| 50,0 | 58,0 |
| 50,0 | 64,6 |
| 50,1 | 39,2 |
| 50,1 | 55,1 |
| 50,1 | 87,6 |
| 50,1 | 73,4 |
| 50,1 | 42,5 |
| 50,2 | 45,3 |
| 50,2 | 6,2 |
| 50,2 | 11,7 |
| 50,2 | 43,9 |
| 50,2 | 31,2 |
| 50,3 | 24,0 |
| 50,3 | 24,4 |
| 50,3 | 21,1 |
| 50,3 | 16,1 |
| 50,3 | -5,0 |
| 50,4 | -7,0 |
| 50,4 | 17,7 |
| 50,4 | 2,0 |
| 50,4 | 8,9 |
| 50,4 | 3,0 |
| 50,5 | 47,0 |
| 50,5 | 13,0 |
| 50,5 | 22,2 |
| 50,5 | 53,5 |
| 50,5 | 37,2 |
| 50,6 | 24,0 |
| 50,6 | 47,3 |
| 50,6 | 26,0 |
| 50,6 | 64,2 |
| 50,6 | 10,0 |
| 50,7 | 16,0 |
| 50,7 | 38,0 |
| 50,7 | 22,1 |
| 50,7 | 12,0 |
| 50,7 | 1,0 |
| 50,8 | 9,0 |
| 50,8 | 21,2 |
| 50,8 | 35,9 |
| 50,8 | 15,9 |
| 50,8 | 27,2 |
| 50,9 | 31,2 |
| 50,9 | 10,0 |
| 50,9 | 12,3 |
| 50,9 | 11,8 |
| 50,9 | 18,0 |
| 51,0 | 28,4 |
| 51,0 | 2,4 |
| 51,0 | -5,0 |
| 51,0 | -11,0 |
| 51,0 | 5,0 |
| 51,1 | 1,0 |
| 51,1 | 8,3 |
| 51,1 | 1,0 |
| 51,1 | 10,8 |
| 51,1 | 37,9 |
| 51,2 | 16,4 |
| 51,2 | 10,7 |
| 51,2 | 20,2 |
| 51,2 | 2,0 |
| 51,2 | -10,0 |
| 51,3 | -15,0 |
| 51,3 | -3,0 |
| 51,3 | 16,4 |
| 51,3 | 24,6 |
| 51,3 | 7,0 |
| 51,4 | 6,0 |
| 51,4 | 9,4 |
| 51,4 | 26,4 |
| 51,4 | -1,0 |
| 51,4 | -7,0 |
| 51,5 | -19,0 |
| 51,5 | 2,1 |
| 51,5 | -15,0 |
| 51,5 | -19,0 |
| 51,5 | -16,0 |
| 51,6 | 0,0 |
| 51,6 | 14,9 |
| 51,6 | 19,2 |
| 51,6 | 5,3 |
| 51,6 | 21,0 |
| 51,7 | 5,3 |
| 51,7 | 7,0 |
| 51,7 | 10,0 |
| 51,7 | 34,3 |
| 51,7 | 6,6 |
| 51,8 | -6,0 |
| 51,8 | 3,2 |
| 51,8 | -10,0 |
| 51,8 | 4,1 |
| 51,8 | 5,7 |
| 51,9 | 7,0 |
| 51,9 | 29,5 |
| 51,9 | 7,8 |
| 51,9 | 22,0 |
| 51,9 | 48,9 |
| 52,0 | 9,0 |
| 52,0 | -2,0 |
| 52,0 | 16,4 |
| 52,0 | 11,0 |
| 52,0 | 28,7 |
| 52,1 | 7,1 |
| 52,1 | -19,0 |
| 52,1 | -25,0 |
| 52,1 | -17,0 |
| 52,1 | 8,2 |
| 52,2 | 3,0 |
| 52,2 | 4,2 |
| 52,2 | 18,5 |
| 52,2 | 17,5 |
| 52,2 | 0,0 |
| 52,3 | -8,0 |
| 52,3 | -8,0 |
| 52,3 | -0,4 |
| 52,3 | 6,0 |
| 52,3 | -0,2 |
| 52,4 | 2,0 |
| 52,4 | 12,6 |
| 52,4 | -7,0 |
| 52,4 | -12,0 |
| 52,4 | -21,0 |
| 52,5 | -20,0 |
| 52,5 | -18,0 |
| 52,5 | -14,0 |
| 52,5 | -9,0 |
| 52,5 | 6,1 |
| 52,6 | 8,7 |
| 52,6 | 24,7 |
| 52,6 | 17,0 |
| 52,6 | 30,5 |
| 52,6 | 13,1 |
| 52,7 | 12,0 |
| 52,7 | 17,4 |
| 52,7 | 22,0 |
| 52,7 | 13,0 |
| 52,7 | 5,8 |
| 52,8 | 6,4 |
| 52,8 | -3,0 |
| 52,8 | -7,0 |
| 52,8 | -0,2 |
| 52,8 | 8,0 |
| 52,9 | 12,4 |
| 52,9 | 36,2 |
| 52,9 | 6,5 |
| 52,9 | 14,0 |
| 52,9 | 30,6 |
| 53,0 | 49,0 |
| 53,0 | 71,3 |
| 53,0 | 31,5 |
| 53,0 | 30,1 |
| 53,0 | 44,5 |
| 53,1 | 35,9 |
| 53,1 | 7,0 |
| 53,1 | 3,0 |
| 53,1 | 0,0 |
| 53,1 | 15,6 |
| 53,2 | 0,0 |
| 53,2 | 2,0 |
| 53,2 | 12,8 |
| 53,2 | 10,5 |
| 53,2 | 0,6 |
| 53,3 | 9,2 |
| 53,3 | 12,0 |
| 53,3 | 33,0 |
| 53,3 | 18,7 |
| 53,3 | 4,0 |
| 53,4 | 1,7 |
| 53,4 | 23,2 |
| 53,4 | 1,4 |
| 53,4 | -12,0 |
| 53,4 | -22,0 |
| 53,5 | -17,0 |
| 53,5 | -16,0 |
| 53,5 | -9,0 |
| 53,5 | -6,0 |
| 53,5 | 14,9 |
| 53,6 | 0,2 |
| 53,6 | 8,8 |
| 53,6 | 15,1 |
| 53,6 | 11,4 |
| 53,6 | -7,0 |
| 53,7 | -17,0 |
| 53,7 | -19,0 |
| 53,7 | -13,0 |
| 53,7 | -9,0 |
| 53,7 | -5,0 |
| 53,8 | -8,0 |
| 53,8 | -7,0 |
| 53,8 | -1,0 |
| 53,8 | 7,5 |
| 53,8 | 11,8 |
| 53,9 | -1,3 |
| 53,9 | -1,0 |
| 53,9 | -3,0 |
| 53,9 | -4,0 |
| 53,9 | -1,6 |
| 54,0 | -12,0 |
| 54,0 | -12,0 |
| 54,0 | -11,0 |
| 54,0 | -4,0 |
| 54,0 | 1,0 |
| 54,1 | 7,5 |
| 54,1 | 3,2 |
| 54,1 | -8,0 |
| 54,1 | -9,0 |
| 54,1 | 14,7 |
| 54,2 | 1,3 |
| 54,2 | 0,0 |
| 54,2 | 0,4 |
| 54,2 | 5,5 |
| 54,2 | 17,7 |
| 54,3 | -3,0 |
| 54,3 | -10,0 |
| 54,3 | -15,0 |
| 54,3 | 9,0 |
| 54,3 | 14,7 |
| 54,4 | 53,1 |
| 54,4 | 13,0 |
| 54,4 | 9,0 |
| 54,4 | 38,9 |
| 54,4 | 0,7 |
| 54,5 | -4,0 |
| 54,5 | -16,0 |
| 54,5 | -18,0 |
| 54,5 | 0,5 |
| 54,5 | -13,0 |
| 54,6 | 0,5 |
| 54,6 | -11,0 |
| 54,6 | -7,0 |
| 54,6 | 5,2 |
| 54,6 | -21,0 |
| 54,7 | -24,0 |
| 54,7 | -21,0 |
| 54,7 | 12,2 |
| 54,7 | 6,3 |
| 54,7 | 20,0 |
| 54,8 | 10,0 |
| 54,8 | 16,5 |
| 54,8 | 10,7 |
| 54,8 | -3,0 |
| 54,8 | -17,0 |
| 54,9 | -18,0 |
| 54,9 | -7,0 |
| 54,9 | 8,8 |
| 54,9 | 15,9 |
| 54,9 | -1,5 |
| 55,0 | 15,2 |
| 55,0 | 11,4 |
| 55,0 | 12,0 |
| 55,0 | 13,5 |
| 55,0 | 17,4 |
| 55,1 | 16,8 |
| 55,1 | 2,0 |
| 55,1 | -1,0 |
| 55,1 | 10,7 |
| 55,1 | 5,0 |
| 55,2 | 13,1 |
| 55,2 | -5,0 |
| 55,2 | -6,0 |
| 55,2 | 2,8 |
| 55,2 | 9,2 |
| 55,3 | 15,6 |
| 55,3 | 10,0 |
| 55,3 | -0,9 |
| 55,3 | -11,0 |
| 55,3 | -5,0 |
| 55,4 | 18,5 |
| 55,4 | 18,4 |
| 55,4 | -1,1 |
| 55,4 | 8,7 |
| 55,4 | 27,8 |
| 55,5 | 24,7 |
| 55,5 | 5,6 |
| 55,5 | -16,0 |
| 55,5 | -20,0 |
| 55,5 | -7,0 |
| 55,6 | 8,9 |
| 55,6 | 20,8 |
| 55,6 | 7,0 |
| 55,6 | 17,8 |
| 55,6 | 3,0 |
| 55,7 | 18,1 |
| 55,7 | -4,0 |
| 55,7 | 0,0 |
| 55,7 | -5,0 |
| 55,7 | 10,4 |
| 55,8 | 0,0 |
| 55,8 | 4,0 |
| 55,8 | 18,3 |
| 55,8 | 1,9 |
| 55,8 | -2,0 |
| 55,9 | -8,0 |
| 55,9 | -2,0 |
| 55,9 | 2,6 |
| 55,9 | 6,4 |
| 55,9 | -3,0 |
| 56,0 | -1,1 |
| 56,0 | 0,4 |
| 56,0 | -4,0 |
| 56,0 | 5,7 |
| 56,0 | 11,0 |
| 56,1 | 35,3 |
| 56,1 | 17,2 |
| 56,1 | 0,9 |
| 56,1 | -0,2 |
| 56,1 | 6,4 |
| 56,2 | 22,4 |
| 56,2 | 21,4 |
| 56,2 | 18,1 |
| 56,2 | 6,0 |
| 56,2 | 4,4 |
| 56,3 | 30,6 |
| 56,3 | 31,6 |
| 56,3 | 19,8 |
| 56,3 | 12,0 |
| 56,3 | 14,1 |
| 56,4 | 23,1 |
| 56,4 | 9,7 |
| 56,4 | 25,0 |
| 56,4 | 19,9 |
| 56,4 | 10,0 |
| 56,5 | 6,3 |
| 56,5 | 4,3 |
| 56,5 | 25,9 |
| 56,5 | 16,2 |
| 56,5 | 4,0 |
| 56,6 | 3,9 |
| 56,6 | -4,0 |
| 56,6 | -1,0 |
| 56,6 | 14,2 |
| 56,6 | -7,0 |
| 56,7 | -14,0 |
| 56,7 | -22,0 |
| 56,7 | -15,0 |
| 56,7 | -6,0 |
| 56,7 | 9,4 |
| 56,8 | 3,9 |
| 56,8 | 16,0 |
| 56,8 | 21,6 |
| 56,8 | 4,4 |
| 56,8 | -1,0 |
| 56,9 | 18,0 |
| 56,9 | -6,0 |
| 56,9 | -13,0 |
| 56,9 | -16,0 |
| 56,9 | -1,4 |
| 57,0 | 10,7 |
| 57,0 | 24,0 |
| 57,0 | 12,6 |
| 57,0 | 8,9 |
| 57,0 | -9,0 |
| 57,1 | -21,0 |
| 57,1 | -19,0 |
| 57,1 | -13,0 |
| 57,1 | 10,1 |
| 57,1 | -9,0 |
| 57,2 | -5,0 |
| 57,2 | 7,7 |
| 57,2 | 12,8 |
| 57,2 | 16,9 |
| 57,2 | 12,3 |
| 57,3 | 4,4 |
| 57,3 | 3,0 |
| 57,3 | 2,0 |
| 57,3 | 11,4 |
| 57,3 | 23,0 |
| 57,4 | 10,0 |
| 57,4 | -2,7 |
| 57,4 | -10,0 |
| 57,4 | -8,0 |
| 57,4 | -2,7 |
| 57,5 | 7,2 |
| 57,5 | 0,2 |
| 57,5 | -2,0 |
| 57,5 | 14,0 |
| 57,5 | 9,4 |
| 57,6 | 4,7 |
| 57,6 | -3,0 |
| 57,6 | 8,0 |
| 57,6 | 25,2 |
| 57,6 | 37,2 |
| 57,7 | 3,6 |
| 57,7 | 3,0 |
| 57,7 | 3,2 |
| 57,7 | 10,8 |
| 57,7 | -1,9 |
| 57,8 | 15,2 |
| 57,8 | 14,3 |
| 57,8 | -3,0 |
| 57,8 | -1,1 |
| 57,8 | -6,0 |
| 57,9 | -5,7 |
| 57,9 | -4,1 |
| 57,9 | -15,0 |
| 57,9 | -13,0 |
| 57,9 | -5,0 |
| 58,0 | 14,7 |
| 58,0 | 7,6 |
| 58,0 | 0,9 |
| 58,0 | -6,7 |
| 58,0 | -5,0 |
| 58,1 | -1,8 |
| 58,1 | 9,1 |
| 58,1 | 1,0 |
| 58,1 | 1,0 |
| 58,1 | 31,1 |
| 58,2 | 4,0 |
| 58,2 | -0,5 |
| 58,2 | -13,0 |
| 58,2 | -5,0 |
| 58,2 | 11,9 |
| 58,3 | -4,0 |
| 58,3 | -8,0 |
| 58,3 | -11,0 |
| 58,3 | -11,0 |
| 58,3 | -1,2 |
| 58,4 | -17,0 |
| 58,4 | -8,0 |
| 58,4 | 4,0 |
| 58,4 | 43,3 |
| 58,4 | 16,4 |
| 58,5 | 2,6 |
| 58,5 | 18,6 |
| 58,5 | 17,4 |
| 58,5 | 30,5 |
| 58,5 | 5,4 |
| 58,6 | -10,0 |
| 58,6 | -23,0 |
| 58,6 | -14,0 |
| 58,6 | -1,0 |
| 58,6 | 19,0 |
| 58,7 | -8,0 |
| 58,7 | -11,0 |
| 58,7 | -3,5 |
| 58,7 | 15,0 |
| 58,7 | 8,7 |
| 58,8 | 26,8 |
| 58,8 | 12,3 |
| 58,8 | 17,4 |
| 58,8 | 27,9 |
| 58,8 | -2,9 |
| 58,9 | 15,2 |
| 58,9 | 12,4 |
| 58,9 | 4,8 |
| 58,9 | 12,6 |
| 58,9 | 11,4 |
| 59,0 | 7,1 |
| 59,0 | 6,0 |
| 59,0 | 0,5 |
| 59,0 | -16,0 |
| 59,0 | -27,0 |
| 59,1 | -21,0 |
| 59,1 | -5,2 |
| 59,1 | 25,3 |
| 59,1 | 12,3 |
| 59,1 | 1,0 |
| 59,2 | -2,9 |
| 59,2 | 7,6 |
| 59,2 | 31,9 |
| 59,2 | 4,3 |
| 59,2 | 1,0 |
| 59,3 | -8,5 |
| 59,3 | 10,0 |
| 59,3 | -2,9 |
| 59,3 | 12,5 |
| 59,3 | -7,8 |
| 59,4 | -18,0 |
| 59,4 | -22,0 |
| 59,4 | -14,0 |
| 59,4 | 1,0 |
| 59,4 | 5,2 |
| 59,5 | 8,8 |
| 59,5 | -9,3 |
| 59,5 | 29,4 |
| 59,5 | 46,1 |
| 59,5 | 25,9 |
| 59,6 | -0,7 |
| 59,6 | 24,7 |
| 59,6 | 57,9 |
| 59,6 | 24,8 |
| 59,6 | 1,4 |
| 59,7 | -1,3 |
| 59,7 | -15,0 |
| 59,7 | -15,0 |
| 59,7 | -24,0 |
| 59,7 | -8,5 |
| 59,8 | -14,0 |
| 59,8 | 8,0 |
| 59,8 | 11,9 |
| 59,8 | 17,7 |
| 59,8 | 69,8 |
| 59,9 | 52,7 |
| 59,9 | 35,8 |
| 59,9 | 16,9 |
| 59,9 | 33,4 |
| 59,9 | 56,9 |
| 60,0 | 18,8 |
| 60,0 | 25,0 |
| 60,0 | 30,3 |
| 60,0 | 18,2 |
| 60,0 | 17,2 |
| 60,1 | 27,3 |
| 60,1 | 21,4 |
| 60,1 | 19,9 |
| 60,1 | 44,0 |
| 60,1 | 41,3 |
| 60,2 | 34,7 |
| 60,2 | 44,6 |
| 60,2 | 36,5 |
| 60,2 | 69,8 |
| 60,2 | 74,8 |
| 60,3 | 70,8 |
| 60,3 | 56,6 |
| 60,3 | 60,0 |
| 60,3 | 91,5 |
| 60,3 | 72,7 |
| 60,4 | 47,8 |
| 60,4 | 51,6 |
| 60,4 | 75,0 |
| 60,4 | 99,2 |
| 60,4 | 122,1 |
| 60,5 | 102,3 |
| 60,5 | 94,8 |
| 60,5 | 106,5 |
| 60,5 | 142,6 |
| 60,5 | 159,9 |
| 60,6 | 124,1 |
| 60,6 | 141,2 |
| 60,6 | 124,3 |
| 60,6 | 140,6 |
| 60,6 | 170,9 |
| 60,7 | 159,9 |
| 60,7 | 200,1 |
| 60,7 | 195,5 |
| 60,7 | 196,9 |
| 60,7 | 180,5 |
| 60,8 | 176,5 |
| 60,8 | 252,4 |
| 60,8 | 187,2 |
| 60,8 | 227,8 |
| 60,8 | 193,7 |
| 60,9 | 253,5 |
| 60,9 | 230,5 |
| 60,9 | 170,2 |
| 60,9 | 168,0 |
| 60,9 | 156,0 |
| 61,0 | 169,6 |
| 61,0 | 153,1 |
| 61,0 | 150,6 |
| 61,0 | 168,9 |
| 61,0 | 141,2 |
| 61,1 | 147,5 |
| 61,1 | 159,3 |
| 61,1 | 179,8 |
| 61,1 | 140,8 |
| 61,1 | 147,0 |
| 61,2 | 141,0 |
| 61,2 | 144,2 |
| 61,2 | 128,1 |
| 61,2 | 104,4 |
| 61,2 | 110,6 |
| 61,3 | 91,6 |
| 61,3 | 115,7 |
| 61,3 | 108,7 |
| 61,3 | 54,7 |
| 61,3 | 49,2 |
| 61,4 | 69,8 |
| 61,4 | 85,3 |
| 61,4 | 82,5 |
| 61,4 | 76,3 |
| 61,4 | 90,2 |
| 61,5 | 51,5 |
| 61,5 | 74,1 |
| 61,5 | 106,1 |
| 61,5 | 84,7 |
| 61,5 | 74,7 |
| 61,6 | 87,6 |
| 61,6 | 58,5 |
| 61,6 | 70,4 |
| 61,6 | 105,6 |
| 61,6 | 89,4 |
| 61,7 | 60,7 |
| 61,7 | 69,4 |
| 61,7 | 80,8 |
| 61,7 | 60,9 |
| 61,7 | 101,4 |
| 61,8 | 138,7 |
| 61,8 | 120,4 |
| 61,8 | 114,0 |
| 61,8 | 115,3 |
| 61,8 | 123,4 |
| 61,9 | 117,7 |
| 61,9 | 130,3 |
| 61,9 | 157,9 |
| 61,9 | 110,9 |
| 61,9 | 98,0 |
| 62,0 | 104,7 |
| 62,0 | 60,3 |
| 62,0 | 60,5 |
| 62,0 | 121,3 |
| 62,0 | 141,7 |
| 62,1 | 115,9 |
| 62,1 | 122,4 |
| 62,1 | 142,3 |
| 62,1 | 149,3 |
| 62,1 | 181,5 |
| 62,2 | 174,5 |
| 62,2 | 147,7 |
| 62,2 | 122,1 |
| 62,2 | 89,5 |
| 62,2 | 134,1 |
| 62,3 | 109,6 |
| 62,3 | 135,2 |
| 62,3 | 68,2 |
| 62,3 | 133,6 |
| 62,3 | 124,6 |
| 62,4 | 101,3 |
| 62,4 | 83,0 |
| 62,4 | 109,2 |
| 62,4 | 92,6 |
| 62,4 | 91,5 |
| 62,5 | 109,4 |
| 62,5 | 94,7 |
| 62,5 | 29,8 |
| 62,5 | 89,7 |
| 62,5 | 89,4 |
| 62,6 | 73,9 |
| 62,6 | 52,4 |
| 62,6 | 50,4 |
| 62,6 | 48,8 |
| 62,6 | 82,1 |
| 62,7 | 63,7 |
| 62,7 | 44,4 |
| 62,7 | 22,0 |
| 62,7 | 20,3 |
| 62,7 | 28,2 |
| 62,8 | 47,6 |
| 62,8 | 29,2 |
| 62,8 | -1,2 |
| 62,8 | 14,2 |
| 62,8 | 29,8 |
| 62,9 | 22,4 |
| 62,9 | 48,9 |
| 62,9 | 49,8 |
| 62,9 | 48,5 |
| 62,9 | 34,0 |
| 63,0 | 10,4 |
| 63,0 | 19,0 |
| 63,0 | 8,8 |
| 63,0 | 22,1 |
| 63,0 | 7,8 |
| 63,1 | 0,0 |
| 63,1 | 4,8 |
| 63,1 | 3,5 |
| 63,1 | 22,6 |
| 63,1 | 17,4 |
| 63,2 | 27,3 |
| 63,2 | 26,6 |
| 63,2 | 12,0 |
| 63,2 | 22,4 |
| 63,2 | 16,4 |
| 63,3 | 13,3 |
| 63,3 | 5,0 |
| 63,3 | 18,0 |
| 63,3 | 33,8 |
| 63,3 | 25,0 |
| 63,4 | 28,6 |
| 63,4 | 18,9 |
| 63,4 | 25,3 |
| 63,4 | -4,8 |
| 63,4 | 17,1 |
| 63,5 | 31,1 |
| 63,5 | 40,6 |
| 63,5 | 21,5 |
| 63,5 | 16,9 |
| 63,5 | 24,6 |
| 63,6 | 32,5 |
| 63,6 | 47,6 |
| 63,6 | 66,1 |
| 63,6 | 43,9 |
| 63,6 | 29,6 |
| 63,7 | 54,9 |
| 63,7 | 45,1 |
| 63,7 | 12,7 |
| 63,7 | -2,0 |
| 63,7 | -5,0 |
| 63,8 | 19,9 |
| 63,8 | 25,2 |
| 63,8 | 53,4 |
| 63,8 | 39,2 |
| 63,8 | 14,7 |
| 63,9 | 28,7 |
| 63,9 | 35,4 |
| 63,9 | 34,3 |
| 63,9 | 87,5 |
| 63,9 | 48,3 |
| 64,0 | 24,6 |
| 64,0 | 18,0 |
| 64,0 | 21,4 |
| 64,0 | 32,7 |
| 64,0 | 41,3 |
| 64,1 | 33,5 |
| 64,1 | 30,8 |
| 64,1 | 40,0 |
| 64,1 | 29,5 |
| 64,1 | 42,3 |
| 64,2 | 41,0 |
| 64,2 | 26,4 |
| 64,2 | 60,1 |
| 64,2 | 75,3 |
| 64,2 | 43,2 |
| 64,3 | 34,7 |
| 64,3 | 28,6 |
| 64,3 | 35,2 |
| 64,3 | 33,4 |
| 64,3 | 18,1 |
| 64,4 | 31,5 |
| 64,4 | 41,6 |
| 64,4 | 45,7 |
| 64,4 | 58,1 |
| 64,4 | 58,4 |
| 64,5 | 52,7 |
| 64,5 | 33,8 |
| 64,5 | 33,2 |
| 64,5 | 60,5 |
| 64,5 | 55,7 |
| 64,6 | 28,2 |
| 64,6 | 40,0 |
| 64,6 | 60,2 |
| 64,6 | 36,9 |
| 64,6 | 55,7 |
| 64,7 | 59,9 |
| 64,7 | 63,0 |
| 64,7 | 55,9 |
| 64,7 | 4,7 |
| 64,7 | 63,2 |
| 64,8 | 47,4 |
| 64,8 | 20,7 |
| 64,8 | 33,6 |
| 64,8 | 36,4 |
| 64,8 | -0,4 |
| 64,9 | 38,3 |
| 64,9 | 25,6 |
| 64,9 | 56,1 |
| 64,9 | 32,6 |
| 64,9 | 8,8 |
| 65,0 | 23,0 |
| 65,0 | 41,7 |
| 65,0 | 27,4 |
| 65,0 | 28,7 |
| 65,0 | 16,1 |
| 65,1 | -7,0 |
| 65,1 | -4,0 |
| 65,1 | 32,0 |
| 65,1 | 24,8 |
| 65,1 | 17,3 |
| 65,2 | 21,2 |
| 65,2 | 14,1 |
| 65,2 | 7,5 |
| 65,2 | 32,4 |
| 65,2 | 37,1 |
| 65,3 | 24,7 |
| 65,3 | 18,7 |
| 65,3 | -2,0 |
| 65,3 | -1,0 |
| 65,3 | 20,9 |
| 65,4 | 13,5 |
| 65,4 | 2,5 |
| 65,4 | 7,2 |
| 65,4 | 0,5 |
| 65,4 | 14,1 |
| 65,5 | 17,1 |
| 65,5 | 23,0 |
| 65,5 | 22,1 |
| 65,5 | 30,3 |
| 65,5 | 15,6 |
| 65,6 | -2,5 |
| 65,6 | -9,0 |
| 65,6 | 4,4 |
| 65,6 | -1,5 |
| 65,6 | -0,5 |
| 65,7 | 18,3 |
| 65,7 | -5,6 |
| 65,7 | -13,0 |
| 65,7 | -5,0 |
| 65,7 | 28,3 |
| 65,8 | 30,2 |
| 65,8 | 4,0 |
| 65,8 | -2,0 |
| 65,8 | -10,5 |
| 65,8 | 4,2 |
| 65,9 | 21,3 |
| 65,9 | -11,5 |
| 65,9 | 13,3 |
| 65,9 | 14,7 |
| 65,9 | 44,5 |
| 66,0 | 9,2 |
| 66,0 | 54,1 |
| 66,0 | 39,1 |
| 66,0 | 49,0 |
| 66,0 | 20,8 |
| 66,1 | 28,7 |
| 66,1 | 37,7 |
| 66,1 | 33,7 |
| 66,1 | 42,9 |
| 66,1 | 11,8 |
| 66,2 | 12,0 |
| 66,2 | 32,2 |
| 66,2 | 28,1 |
| 66,2 | 18,8 |
| 66,2 | 26,9 |
| 66,3 | 17,9 |
| 66,3 | 39,2 |
| 66,3 | -3,9 |
| 66,3 | 2,2 |
| 66,3 | 32,5 |
| 66,4 | 27,6 |
| 66,4 | 11,8 |
| 66,4 | 11,4 |
| 66,4 | 31,2 |
| 66,4 | 42,5 |
| 66,5 | 7,1 |
| 66,5 | -4,6 |
| 66,5 | 2,9 |
| 66,5 | -1,7 |
| 66,5 | 6,3 |
| 66,6 | -11,8 |
| 66,6 | -12,8 |
| 66,6 | -1,0 |
| 66,6 | -9,8 |
| 66,6 | -12,1 |
| 66,7 | -7,0 |
| 66,7 | 2,2 |
| 66,7 | 17,6 |
| 66,7 | 3,3 |
| 66,7 | 4,4 |
| 66,8 | 20,6 |
| 66,8 | 33,2 |
| 66,8 | 24,7 |
| 66,8 | -2,9 |
| 66,8 | -6,0 |
| 66,9 | 1,2 |
| 66,9 | 23,5 |
| 66,9 | 31,5 |
| 66,9 | 20,1 |
| 66,9 | -8,6 |
| 67,0 | -25,0 |
| 67,0 | -28,0 |
| 67,0 | -8,8 |
| 67,0 | -0,9 |
| 67,0 | 10,7 |
| 67,1 | 19,8 |
| 67,1 | 34,6 |
| 67,1 | 9,0 |
| 67,1 | 16,1 |
| 67,1 | -8,5 |
| 67,2 | 6,1 |
| 67,2 | 4,9 |
| 67,2 | 1,8 |
| 67,2 | 39,7 |
| 67,2 | -2,5 |
| 67,3 | -10,1 |
| 67,3 | -17,0 |
| 67,3 | 8,2 |
| 67,3 | -14,5 |
| 67,3 | 34,6 |
| 67,4 | -4,2 |
| 67,4 | -6,8 |
| 67,4 | -12,6 |
| 67,4 | -6,4 |
| 67,4 | -21,0 |
| 67,5 | -15,0 |
| 67,5 | 13,2 |
| 67,5 | -1,7 |
| 67,5 | -7,0 |
| 67,5 | -10,4 |
| 67,6 | -17,2 |
| 67,6 | -14,7 |
| 67,6 | 6,6 |
| 67,6 | 34,3 |
| 67,6 | 14,7 |
| 67,7 | -8,6 |
| 67,7 | -4,3 |
| 67,7 | 2,3 |
| 67,7 | 1,1 |
| 67,7 | 5,3 |
| 67,8 | -7,0 |
| 67,8 | -4,1 |
| 67,8 | -3,6 |
| 67,8 | -11,3 |
| 67,8 | 0,9 |
| 67,9 | 14,8 |
| 67,9 | 7,0 |
| 67,9 | -15,1 |
| 67,9 | -11,9 |
| 67,9 | -19,5 |
| 68,0 | -9,9 |
| 68,0 | -3,8 |
| 68,0 | -12,8 |
| 68,0 | -1,1 |
| 68,0 | 14,9 |
| 68,1 | 7,5 |
| 68,1 | 6,4 |
| 68,1 | 2,9 |
| 68,1 | -3,0 |
| 68,1 | 6,6 |
| 68,2 | 21,0 |
| 68,2 | 8,2 |
| 68,2 | 21,3 |
| 68,2 | 25,0 |
| 68,2 | -0,3 |
| 68,3 | 8,4 |
| 68,3 | 23,4 |
| 68,3 | 29,7 |
| 68,3 | -11,8 |
| 68,3 | -16,0 |
| 68,4 | -5,4 |
| 68,4 | -16,4 |
| 68,4 | 6,8 |
| 68,4 | 4,3 |
| 68,4 | -4,2 |
| 68,5 | 5,0 |
| 68,5 | -11,0 |
| 68,5 | -1,0 |
| 68,5 | -4,8 |
| 68,5 | -19,9 |
| 68,6 | -6,7 |
| 68,6 | 22,6 |
| 68,6 | 6,4 |
| 68,6 | -9,2 |
| 68,6 | 0,5 |
| 68,7 | -17,0 |
| 68,7 | -11,4 |
| 68,7 | -2,2 |
| 68,7 | 12,5 |
| 68,7 | 14,0 |
| 68,8 | 20,2 |
| 68,8 | -9,3 |
| 68,8 | 9,5 |
| 68,8 | -8,1 |
| 68,8 | 5,1 |
| 68,9 | 1,4 |
| 68,9 | -21,3 |
| 68,9 | -0,7 |
| 68,9 | -11,4 |
| 68,9 | -17,4 |
| 69,0 | -8,0 |
| 69,0 | 10,3 |
| 69,0 | 45,1 |
| 69,0 | 6,6 |
| 69,0 | -20,5 |
| 69,1 | 2,4 |
| 69,1 | 14,4 |
| 69,1 | 19,9 |
| 69,1 | 23,2 |
| 69,1 | -2,1 |
| 69,2 | -8,8 |
| 69,2 | -6,0 |
| 69,2 | -15,0 |
| 69,2 | -14,1 |
| 69,2 | -12,8 |
| 69,3 | -2,0 |
| 69,3 | 11,4 |
| 69,3 | -4,6 |
| 69,3 | -6,0 |
| 69,3 | 9,2 |
| 69,4 | -4,6 |
| 69,4 | 12,4 |
| 69,4 | 6,8 |
| 69,4 | -19,7 |
| 69,4 | -12,1 |
| 69,5 | -8,4 |
| 69,5 | -10,9 |
| 69,5 | 14,9 |
| 69,5 | 23,0 |
| 69,5 | 19,0 |
| 69,6 | -1,3 |
| 69,6 | -14,6 |
| 69,6 | -11,4 |
| 69,6 | -2,9 |
| 69,6 | -1,4 |
| 69,7 | 6,8 |
| 69,7 | 16,5 |
| 69,7 | 10,0 |
| 69,7 | -21,3 |
| 69,7 | -15,7 |
| 69,8 | 0,7 |
| 69,8 | 6,9 |
| 69,8 | 18,5 |
| 69,8 | 17,2 |
| 69,8 | 41,3 |
| 69,9 | 17,1 |
| 69,9 | -23,5 |
| 69,9 | -13,8 |
| 69,9 | 7,8 |
| 69,9 | 2,5 |
| 70,0 | 20,9 |
| 70,0 | 7,9 |
| 70,0 | 14,9 |
| 70,0 | -2,7 |
| 70,0 | 5,1 |
| 70,1 | 4,8 |
| 70,1 | -8,8 |
| 70,1 | -13,2 |
| 70,1 | 8,3 |
| 70,1 | 6,9 |
| 70,2 | -17,9 |
| 70,2 | 11,5 |
| 70,2 | -2,1 |
| 70,2 | -12,0 |
| 70,2 | 12,0 |
| 70,3 | 10,6 |
| 70,3 | 5,8 |
| 70,3 | 6,9 |
| 70,3 | 12,9 |
| 70,3 | 20,6 |
| 70,4 | 12,2 |
| 70,4 | 1,6 |
| 70,4 | 12,9 |
| 70,4 | 8,9 |
| 70,4 | 7,2 |
| 70,5 | 23,1 |
| 70,5 | 22,0 |
| 70,5 | 14,0 |
| 70,5 | 10,0 |
| 70,5 | 13,7 |
| 70,6 | 24,7 |
| 70,6 | 38,9 |
| 70,6 | 26,3 |
| 70,6 | 23,9 |
| 70,6 | 27,8 |
| 70,7 | 30,2 |
| 70,7 | 30,5 |
| 70,7 | 30,3 |
| 70,7 | 17,5 |
| 70,7 | -0,3 |
| 70,8 | -5,2 |
| 70,8 | 7,2 |
| 70,8 | 29,4 |
| 70,8 | 35,4 |
| 70,8 | 30,2 |
| 70,9 | 37,2 |
| 70,9 | 17,5 |
| 70,9 | 15,5 |
| 70,9 | -9,7 |
| 70,9 | 17,7 |
| 71,0 | 35,1 |
| 71,0 | 42,7 |
| 71,0 | 29,3 |
| 71,0 | 18,1 |
| 71,0 | 30,6 |
| 71,1 | 30,0 |
| 71,1 | 12,1 |
| 71,1 | 28,7 |
| 71,1 | 36,2 |
| 71,1 | 24,9 |
| 71,2 | 16,9 |
| 71,2 | 3,8 |
| 71,2 | 8,4 |
| 71,2 | 14,0 |
| 71,2 | 35,6 |
| 71,3 | 25,0 |
| 71,3 | 32,1 |
| 71,3 | 18,0 |
| 71,3 | 54,5 |
| 71,3 | 20,1 |
| 71,4 | 38,8 |
| 71,4 | 19,2 |
| 71,4 | 12,4 |
| 71,4 | 41,6 |
| 71,4 | 49,7 |
| 71,5 | 22,4 |
| 71,5 | 31,8 |
| 71,5 | 32,3 |
| 71,5 | 0,7 |
| 71,5 | 24,9 |
| 71,6 | 39,3 |
| 71,6 | 44,4 |
| 71,6 | 44,0 |
| 71,6 | 32,7 |
| 71,6 | -7,8 |
| 71,7 | 36,8 |
| 71,7 | 41,3 |
| 71,7 | 18,1 |
| 71,7 | 29,9 |
| 71,7 | 37,9 |
| 71,8 | 37,2 |
| 71,8 | 34,5 |
| 71,8 | 42,9 |
| 71,8 | 44,9 |
| 71,8 | 61,9 |
| 71,9 | 40,9 |
| 71,9 | 22,7 |
| 71,9 | 34,9 |
| 71,9 | 33,8 |
| 71,9 | 45,7 |
| 72,0 | 57,9 |
| 72,0 | 55,8 |
| 72,0 | 17,0 |
| 72,0 | 17,8 |
| 72,0 | 40,4 |
| 72,1 | 37,1 |
| 72,1 | 53,0 |
| 72,1 | 41,4 |
| 72,1 | 47,2 |
| 72,1 | 31,6 |
| 72,2 | 43,6 |
| 72,2 | 36,8 |
| 72,2 | 48,8 |
| 72,2 | 52,4 |
| 72,2 | 50,8 |
| 72,3 | 30,2 |
| 72,3 | 23,2 |
| 72,3 | 23,3 |
| 72,3 | 20,8 |
| 72,3 | 25,1 |
| 72,4 | 71,6 |
| 72,4 | 55,0 |
| 72,4 | 34,9 |
| 72,4 | 31,7 |
| 72,4 | 24,3 |
| 72,5 | 36,0 |
| 72,5 | 68,3 |
| 72,5 | 44,4 |
| 72,5 | 47,1 |
| 72,5 | 33,8 |
| 72,6 | 11,4 |
| 72,6 | 0,4 |
| 72,6 | 28,0 |
| 72,6 | 50,7 |
| 72,6 | 35,7 |
| 72,7 | 23,6 |
| 72,7 | 7,7 |
| 72,7 | 22,6 |
| 72,7 | 16,0 |
| 72,7 | 30,1 |
| 72,8 | 3,6 |
| 72,8 | 36,2 |
| 72,8 | 3,3 |
| 72,8 | -10,4 |
| 72,8 | 14,2 |
| 72,9 | 27,7 |
| 72,9 | 24,8 |
| 72,9 | 8,4 |
| 72,9 | 19,6 |
| 72,9 | 25,1 |
| 73,0 | -9,0 |
| 73,0 | 2,5 |
| 73,0 | 9,3 |
| 73,0 | 12,9 |
| 73,0 | 22,6 |
| 73,1 | 15,4 |
| 73,1 | 17,7 |
| 73,1 | 30,8 |
| 73,1 | 9,7 |
| 73,1 | 12,2 |
| 73,2 | 17,0 |
| 73,2 | -1,8 |
| 73,2 | -7,1 |
| 73,2 | 0,9 |
| 73,2 | 15,3 |
| 73,3 | -4,6 |
| 73,3 | 1,0 |
| 73,3 | -0,5 |
| 73,3 | 0,4 |
| 73,3 | 17,6 |
| 73,4 | 4,9 |
| 73,4 | 24,2 |
| 73,4 | 30,8 |
| 73,4 | -5,1 |
| 73,4 | 23,2 |
| 73,5 | 11,9 |
| 73,5 | 17,7 |
| 73,5 | 36,4 |
| 73,5 | 28,9 |
| 73,5 | 6,9 |
| 73,6 | -3,8 |
| 73,6 | -0,5 |
| 73,6 | -10,0 |
| 73,6 | -3,1 |
| 73,6 | 9,2 |
| 73,7 | 4,6 |
| 73,7 | -8,9 |
| 73,7 | -3,7 |
| 73,7 | 11,3 |
| 73,7 | 12,1 |
| 73,8 | 28,1 |
| 73,8 | 7,1 |
| 73,8 | 31,1 |
| 73,8 | 31,0 |
| 73,8 | 12,4 |
| 73,9 | -3,4 |
| 73,9 | 30,9 |
| 73,9 | 16,3 |
| 73,9 | 24,5 |
| 73,9 | 22,8 |
| 74,0 | -15,5 |
| 74,0 | 12,0 |
| 74,0 | 16,6 |
| 74,0 | 10,9 |
| 74,0 | -10,4 |
| 74,1 | -16,1 |
| 74,1 | 11,8 |
| 74,1 | 2,1 |
| 74,1 | -14,2 |
| 74,1 | -2,1 |
| 74,2 | 0,9 |
| 74,2 | 17,9 |
| 74,2 | 14,3 |
| 74,2 | 10,8 |
| 74,2 | 29,9 |
| 74,3 | 8,0 |
| 74,3 | 20,1 |
| 74,3 | 22,9 |
| 74,3 | 24,4 |
| 74,3 | 17,7 |
| 74,4 | 5,5 |
| 74,4 | 8,5 |
| 74,4 | 29,5 |
| 74,4 | 37,0 |
| 74,4 | 17,1 |
| 74,5 | 0,2 |
| 74,5 | 15,6 |
| 74,5 | 33,5 |
| 74,5 | 35,4 |
| 74,5 | 18,6 |
| 74,6 | 10,2 |
| 74,6 | 28,1 |
| 74,6 | 49,0 |
| 74,6 | 18,9 |
| 74,6 | -8,0 |
| 74,7 | 12,0 |
| 74,7 | 19,0 |
| 74,7 | 12,3 |
| 74,7 | 8,6 |
| 74,7 | 23,6 |
| 74,8 | 39,7 |
| 74,8 | 24,7 |
| 74,8 | 8,3 |
| 74,8 | -8,6 |
| 74,8 | 29,9 |
| 74,9 | 41,2 |
| 74,9 | 30,7 |
| 74,9 | 18,2 |
| 74,9 | 24,6 |
| 74,9 | 34,0 |
| 75,0 | 1,2 |
| 75,0 | 15,6 |
| 75,0 | 73,4 |
| 75,0 | 43,5 |
| 75,0 | 41,0 |
| 75,1 | 20,5 |
| 75,1 | 27,2 |
| 75,1 | 36,0 |
| 75,1 | 63,6 |
| 75,1 | 72,5 |
| 75,2 | 49,4 |
| 75,2 | 17,2 |
| 75,2 | 32,5 |
| 75,2 | 31,5 |
| 75,2 | 22,7 |
| 75,3 | 17,3 |
| 75,3 | 34,1 |
| 75,3 | 10,0 |
| 75,3 | 15,3 |
| 75,3 | 1,9 |
| 75,4 | 0,3 |
| 75,4 | 36,8 |
| 75,4 | 21,8 |
| 75,4 | 22,2 |
| 75,4 | 6,7 |
| 75,5 | 21,2 |
| 75,5 | 46,4 |
| 75,5 | 40,6 |
| 75,5 | 62,1 |
| 75,5 | 47,0 |
| 75,6 | 31,6 |
| 75,6 | 22,1 |
| 75,6 | 14,5 |
| 75,6 | 29,8 |
| 75,6 | 12,1 |
| 75,7 | 10,9 |
| 75,7 | 18,2 |
| 75,7 | 1,7 |
| 75,7 | 10,4 |
| 75,7 | 16,0 |
| 75,8 | 1,3 |
| 75,8 | -7,8 |
| 75,8 | -0,5 |
| 75,8 | 9,5 |
| 75,8 | 18,6 |
| 75,9 | 39,3 |
| 75,9 | 20,9 |
| 75,9 | 13,3 |
| 75,9 | 6,9 |
| 75,9 | -10,3 |
| 76,0 | -8,6 |
| 76,0 | 15,9 |
| 76,0 | 22,4 |
| 76,0 | 32,5 |
| 76,0 | 15,1 |
| 76,1 | 2,4 |
| 76,1 | 0,6 |
| 76,1 | 27,5 |
| 76,1 | 18,4 |
| 76,1 | 6,0 |
| 76,2 | 9,0 |
| 76,2 | 7,9 |
| 76,2 | -8,5 |
| 76,2 | 13,8 |
| 76,2 | 17,0 |
| 76,3 | 1,8 |
| 76,3 | 18,7 |
| 76,3 | 26,7 |
| 76,3 | 3,4 |
| 76,3 | 11,7 |
| 76,4 | 9,0 |
| 76,4 | 4,5 |
| 76,4 | 7,8 |
| 76,4 | 33,3 |
| 76,4 | 13,5 |
| 76,5 | 7,8 |
| 76,5 | -5,8 |
| 76,5 | 4,2 |
| 76,5 | -16,0 |
| 76,5 | -9,0 |
| 76,6 | -7,3 |
| 76,6 | 30,7 |
| 76,6 | 6,2 |
| 76,6 | 1,8 |
| 76,6 | 2,1 |
| 76,7 | -7,0 |
| 76,7 | -3,2 |
| 76,7 | -0,2 |
| 76,7 | 3,3 |
| 76,7 | 12,7 |
| 76,8 | 20,4 |
| 76,8 | -5,9 |
| 76,8 | -1,0 |
| 76,8 | 22,1 |
| 76,8 | 13,1 |
| 76,9 | 16,4 |
| 76,9 | 34,6 |
| 76,9 | 12,5 |
| 76,9 | 13,8 |
| 76,9 | 7,1 |
| 77,0 | -3,7 |
| 77,0 | -13,6 |
| 77,0 | 5,9 |
| 77,0 | 8,5 |
| 77,0 | -0,1 |
| 77,1 | 11,9 |
| 77,1 | 5,6 |
| 77,1 | -3,2 |
| 77,1 | 6,7 |
| 77,1 | 0,8 |
| 77,2 | -3,1 |
| 77,2 | 14,7 |
| 77,2 | 23,8 |
| 77,2 | 5,6 |
| 77,2 | 0,2 |
| 77,3 | -11,4 |
| 77,3 | -7,6 |
| 77,3 | -14,0 |
| 77,3 | -9,1 |
| 77,3 | -23,0 |
| 77,4 | -21,0 |
| 77,4 | -13,0 |
| 77,4 | 3,4 |
| 77,4 | 15,5 |
| 77,4 | -3,1 |
| 77,5 | 30,7 |
| 77,5 | 17,7 |
| 77,5 | 15,2 |
| 77,5 | -6,4 |
| 77,5 | 6,2 |
| 77,6 | 0,0 |
| 77,6 | 18,6 |
| 77,6 | 5,4 |
| 77,6 | 4,9 |
| 77,6 | 16,8 |
| 77,7 | 3,0 |
| 77,7 | 4,5 |
| 77,7 | 1,7 |
| 77,7 | 6,0 |
| 77,7 | 27,6 |
| 77,8 | 8,0 |
| 77,8 | -1,0 |
| 77,8 | -10,0 |
| 77,8 | 0,5 |
| 77,8 | -2,4 |
| 77,9 | -4,0 |
| 77,9 | -8,5 |
| 77,9 | 23,2 |
| 77,9 | 2,6 |
| 77,9 | 7,0 |
| 78,0 | -18,0 |
| 78,0 | -16,0 |
| 78,0 | -0,1 |
| 78,0 | 6,2 |
| 78,0 | -8,9 |
| 78,1 | -8,0 |
| 78,1 | 1,1 |
| 78,1 | 14,7 |
| 78,1 | 5,8 |
| 78,1 | 3,6 |
| 78,2 | -3,0 |
| 78,2 | 1,2 |
| 78,2 | 11,5 |
| 78,2 | 0,6 |
| 78,2 | 0,6 |
| 78,3 | 0,6 |
| 78,3 | 4,2 |
| 78,3 | 9,5 |
| 78,3 | 3,5 |
| 78,3 | 2,5 |
| 78,4 | 5,4 |
| 78,4 | -9,6 |
| 78,4 | 2,2 |
| 78,4 | 11,8 |
| 78,4 | 7,0 |
| 78,5 | 1,4 |
| 78,5 | 17,4 |
| 78,5 | 23,2 |
| 78,5 | 4,8 |
| 78,5 | 8,5 |
| 78,6 | -3,8 |
| 78,6 | -9,6 |
| 78,6 | -7,0 |
| 78,6 | 9,2 |
| 78,6 | -4,2 |
| 78,7 | 5,2 |
| 78,7 | 0,7 |
| 78,7 | 8,9 |
| 78,7 | 8,4 |
| 78,7 | -1,8 |
| 78,8 | 10,3 |
| 78,8 | -2,0 |
| 78,8 | 8,2 |
| 78,8 | -5,8 |
| 78,8 | 11,1 |
| 78,9 | -5,0 |
| 78,9 | -8,8 |
| 78,9 | -2,8 |
| 78,9 | -2,0 |
| 78,9 | 20,0 |
| 79,0 | 1,5 |
| 79,0 | 27,3 |
| 79,0 | -14,0 |
| 79,0 | -18,0 |
| 79,0 | -15,0 |
| 79,1 | 14,4 |
| 79,1 | 7,9 |
| 79,1 | -8,0 |
| 79,1 | -6,6 |
| 79,1 | -8,0 |
| 79,2 | 7,5 |
| 79,2 | 8,9 |
| 79,2 | -13,0 |
| 79,2 | -20,0 |
| 79,2 | -14,0 |
| 79,3 | 9,7 |
| 79,3 | 16,3 |
| 79,3 | 21,1 |
| 79,3 | 16,5 |
| 79,3 | 5,1 |
| 79,4 | 17,3 |
| 79,4 | 11,9 |
| 79,4 | -3,1 |
| 79,4 | 3,7 |
| 79,4 | 13,8 |
| 79,5 | 4,4 |
| 79,5 | 18,5 |
| 79,5 | 34,3 |
| 79,5 | 2,7 |
| 79,5 | -3,6 |
| 79,6 | -7,6 |
| 79,6 | -2,8 |
| 79,6 | -7,9 |
| 79,6 | 12,1 |
| 79,6 | 6,0 |
| 79,7 | -2,4 |
| 79,7 | 2,1 |
| 79,7 | -3,5 |
| 79,7 | -6,0 |
| 79,7 | -4,0 |
| 79,8 | 9,5 |
| 79,8 | 9,2 |
| 79,8 | 5,2 |
| 79,8 | 3,4 |
| 79,8 | -3,8 |
| 79,9 | -9,0 |
| 79,9 | -10,5 |
| 79,9 | 3,0 |
| 79,9 | 10,0 |
| 79,9 | -5,4 |
| 80,0 | -4,0 |
| 80,0 | 11,9 |
| 80,0 | 25,6 |
| 80,0 | 24,7 |
| 80,0 | 7,0 |
| 80,1 | 12,8 |
| 80,1 | 0,2 |
| 80,1 | 13,5 |
| 80,1 | 17,6 |
| 80,1 | 2,1 |
| 80,2 | 7,8 |
| 80,2 | 26,5 |
| 80,2 | 10,2 |
| 80,2 | 55,0 |
| 80,2 | 23,8 |
| 80,3 | 3,0 |
| 80,3 | -1,8 |
| 80,3 | 7,9 |
| 80,3 | 16,9 |
| 80,3 | 8,1 |
| 80,4 | -10,3 |
| 80,4 | 0,7 |
| 80,4 | 16,7 |
| 80,4 | -7,0 |
| 80,4 | -1,6 |
| 80,5 | -21,0 |
| 80,5 | -6,1 |
| 80,5 | -2,6 |
| 80,5 | 8,8 |
| 80,5 | 4,1 |
| 80,6 | -6,2 |
| 80,6 | -3,4 |
| 80,6 | -11,0 |
| 80,6 | -0,7 |
| 80,6 | -8,4 |
| 80,7 | 6,8 |
| 80,7 | -4,6 |
| 80,7 | -2,0 |
| 80,7 | 1,1 |
| 80,7 | 22,1 |
| 80,8 | 11,1 |
| 80,8 | 1,8 |
| 80,8 | 3,3 |
| 80,8 | 2,4 |
| 80,8 | 14,5 |
| 80,9 | 3,6 |
| 80,9 | 11,3 |
| 80,9 | 32,5 |
| 80,9 | 15,5 |
| 80,9 | 1,7 |
| 81,0 | 7,9 |
| 81,0 | -8,7 |
| 81,0 | -17,0 |
| 81,0 | -21,0 |
| 81,0 | -19,0 |
| 81,1 | -6,9 |
| 81,1 | -5,0 |
| 81,1 | 0,6 |
| 81,1 | 2,4 |
| 81,1 | -9,0 |
| 81,2 | -9,0 |
| 81,2 | 0,4 |
| 81,2 | 4,1 |
| 81,2 | -9,9 |
| 81,2 | -3,1 |
| 81,3 | 18,1 |
| 81,3 | 11,9 |
| 81,3 | -4,9 |
| 81,3 | -7,2 |
| 81,3 | 21,2 |
| 81,4 | 1,3 |
| 81,4 | -3,5 |
| 81,4 | -8,0 |
| 81,4 | 0,4 |
| 81,4 | 7,6 |
| 81,5 | -2,7 |
| 81,5 | 1,8 |
| 81,5 | -2,0 |
| 81,5 | 16,9 |
| 81,5 | -3,0 |
| 81,6 | -3,5 |
| 81,6 | 6,7 |
| 81,6 | 15,0 |
| 81,6 | -1,0 |
| 81,6 | 0,4 |
| 81,7 | -0,7 |
| 81,7 | -8,8 |
| 81,7 | -2,0 |
| 81,7 | 2,8 |
| 81,7 | 23,1 |
| 81,8 | 0,0 |
| 81,8 | -1,0 |
| 81,8 | -2,1 |
| 81,8 | 12,7 |
| 81,8 | -2,0 |
| 81,9 | 5,3 |
| 81,9 | -4,0 |
| 81,9 | -1,5 |
| 81,9 | 15,8 |
| 81,9 | 3,4 |
| 82,0 | 0,3 |
| 82,0 | 1,2 |
| 82,0 | 6,7 |
| 82,0 | 24,1 |
| 82,0 | 11,7 |
| 82,1 | -2,0 |
| 82,1 | -2,5 |
| 82,1 | -7,0 |
| 82,1 | -0,1 |
| 82,1 | 13,6 |
| 82,2 | 12,5 |
| 82,2 | 9,7 |
| 82,2 | 13,4 |
| 82,2 | 4,2 |
| 82,2 | -5,6 |
| 82,3 | -7,0 |
| 82,3 | -2,1 |
| 82,3 | 1,7 |
| 82,3 | 2,6 |
| 82,3 | 19,1 |
| 82,4 | 16,7 |
| 82,4 | 2,6 |
| 82,4 | 2,1 |
| 82,4 | 10,5 |
| 82,4 | 16,9 |
| 82,5 | 3,9 |
| 82,5 | 2,4 |
| 82,5 | -4,0 |
| 82,5 | -3,0 |
| 82,5 | 9,6 |
| 82,6 | -1,7 |
| 82,6 | -3,5 |
| 82,6 | -4,0 |
| 82,6 | 8,5 |
| 82,6 | 21,3 |
| 82,7 | -5,9 |
| 82,7 | -4,9 |
| 82,7 | -18,0 |
| 82,7 | -21,0 |
| 82,7 | 2,1 |
| 82,8 | 3,0 |
| 82,8 | 23,1 |
| 82,8 | 8,2 |
| 82,8 | 1,0 |
| 82,8 | 3,4 |
| 82,9 | 1,8 |
| 82,9 | 7,6 |
| 82,9 | -3,0 |
| 82,9 | 1,5 |
| 82,9 | 30,3 |
| 83,0 | -3,0 |
| 83,0 | -5,0 |
| 83,0 | -18,0 |
| 83,0 | -9,0 |
| 83,0 | -4,0 |
| 83,1 | 20,7 |
| 83,1 | 5,0 |
| 83,1 | 4,4 |
| 83,1 | -10,0 |
| 83,1 | -6,0 |
| 83,2 | 13,8 |
| 83,2 | -5,0 |
| 83,2 | -5,0 |
| 83,2 | -7,0 |
| 83,2 | 5,8 |
| 83,3 | 1,9 |
| 83,3 | -0,6 |
| 83,3 | -18,0 |
| 83,3 | -19,0 |
| 83,3 | -1,4 |
| 83,4 | -1,1 |
| 83,4 | 14,8 |
| 83,4 | 15,1 |
| 83,4 | 1,8 |
| 83,4 | -3,0 |
| 83,5 | -2,8 |
| 83,5 | 6,8 |
| 83,5 | 16,0 |
| 83,5 | 0,6 |
| 83,5 | -2,6 |
| 83,6 | 9,8 |
| 83,6 | 1,0 |
| 83,6 | 0,0 |
| 83,6 | -3,6 |
| 83,6 | 6,8 |
| 83,7 | -1,0 |
| 83,7 | 1,5 |
| 83,7 | 9,7 |
| 83,7 | 8,2 |
| 83,7 | 8,2 |
| 83,8 | -2,0 |
| 83,8 | -13,0 |
| 83,8 | -2,1 |
| 83,8 | 0,0 |
| 83,8 | 25,9 |
| 83,9 | 19,2 |
| 83,9 | 4,5 |
| 83,9 | 25,4 |
| 83,9 | 0,0 |
| 83,9 | -13,0 |
| 84,0 | -19,0 |
| 84,0 | -8,0 |
| 84,0 | 15,8 |
| 84,0 | 15,7 |
| 84,0 | 2,0 |
| 84,1 | -2,3 |
| 84,1 | -12,0 |
| 84,1 | -18,0 |
| 84,1 | -1,0 |
| 84,1 | -8,0 |
| 84,2 | 0,6 |
| 84,2 | 11,6 |
| 84,2 | 13,0 |
| 84,2 | 28,3 |
| 84,2 | 10,1 |
| 84,3 | -3,0 |
| 84,3 | -14,0 |
| 84,3 | -0,8 |
| 84,3 | 2,0 |
| 84,3 | 2,7 |
| 84,4 | 19,0 |
| 84,4 | -3,1 |
| 84,4 | 14,8 |
| 84,4 | 1,0 |
| 84,4 | -1,1 |
| 84,5 | 5,1 |
| 84,5 | -4,0 |
| 84,5 | -11,0 |
| 84,5 | -16,0 |
| 84,5 | -5,0 |
| 84,6 | 3,5 |
| 84,6 | 35,4 |
| 84,6 | 18,9 |
| 84,6 | 3,0 |
| 84,6 | -4,0 |
| 84,7 | 3,1 |
| 84,7 | 28,9 |
| 84,7 | 33,4 |
| 84,7 | 27,0 |
| 84,7 | 16,8 |
| 84,8 | 14,0 |
| 84,8 | 9,1 |
| 84,8 | 1,1 |
| 84,8 | 0,0 |
| 84,8 | 0,2 |
| 84,9 | 27,1 |
| 84,9 | 16,1 |
| 84,9 | 6,0 |
| 84,9 | -12,0 |
| 84,9 | -19,0 |
| 85,0 | -13,0 |
| 85,0 | -9,0 |
| 85,0 | 15,9 |
| 85,0 | 11,0 |
| 85,0 | 11,1 |
| 85,1 | 36,5 |
| 85,1 | 18,8 |
| 85,1 | 10,0 |
| 85,1 | 2,0 |
| 85,1 | 8,1 |
| 85,2 | 5,8 |
| 85,2 | 8,5 |
| 85,2 | 13,6 |
| 85,2 | 44,3 |
| 85,2 | 19,1 |
| 85,3 | 4,4 |
| 85,3 | 0,0 |
| 85,3 | 14,6 |
| 85,3 | 1,0 |
| 85,3 | 15,0 |
| 85,4 | 30,2 |
| 85,4 | 36,4 |
| 85,4 | -0,7 |
| 85,4 | 20,8 |
| 85,4 | 3,5 |
| 85,5 | 18,4 |
| 85,5 | -1,0 |
| 85,5 | 3,0 |
| 85,5 | 23,7 |
| 85,5 | 4,1 |
| 85,6 | 5,0 |
| 85,6 | 7,0 |
| 85,6 | 29,6 |
| 85,6 | 8,0 |
| 85,6 | -4,0 |
| 85,7 | 5,5 |
| 85,7 | -1,0 |
| 85,7 | 5,0 |
| 85,7 | 37,7 |
| 85,7 | 23,8 |
| 85,8 | 3,0 |
| 85,8 | 9,6 |
| 85,8 | 19,4 |
| 85,8 | 6,4 |
| 85,8 | 0,0 |
| 85,9 | -2,5 |
| 85,9 | -4,0 |
| 85,9 | 2,1 |
| 85,9 | 20,5 |
| 85,9 | 20,8 |
| 86,0 | 29,8 |
| 86,0 | 8,0 |
| 86,0 | -2,0 |
| 86,0 | -0,1 |
| 86,0 | -7,0 |
| 86,1 | -3,0 |
| 86,1 | 8,4 |
| 86,1 | 4,5 |
| 86,1 | 4,0 |
| 86,1 | -2,8 |
| 86,2 | 3,7 |
| 86,2 | -19,0 |
| 86,2 | -19,0 |
| 86,2 | -12,0 |
| 86,2 | 7,2 |
| 86,3 | 7,8 |
| 86,3 | 7,9 |
| 86,3 | 22,5 |
| 86,3 | 16,9 |
| 86,3 | 3,0 |
| 86,4 | -1,4 |
| 86,4 | 0,8 |
| 86,4 | 11,3 |
| 86,4 | 10,5 |
| 86,4 | 3,5 |
| 86,5 | 9,4 |
| 86,5 | 17,2 |
| 86,5 | 12,9 |
| 86,5 | 1,8 |
| 86,5 | -8,0 |
| 86,6 | -17,0 |
| 86,6 | -11,0 |
| 86,6 | 0,0 |
| 86,6 | 25,9 |
| 86,6 | 8,7 |
| 86,7 | -3,1 |
| 86,7 | -12,0 |
| 86,7 | -13,0 |
| 86,7 | -11,0 |
| 86,7 | 10,9 |
| 86,8 | -15,0 |
| 86,8 | -9,0 |
| 86,8 | -10,0 |
| 86,8 | 12,4 |
| 86,8 | 11,3 |
| 86,9 | 2,1 |
| 86,9 | -5,0 |
| 86,9 | -2,6 |
| 86,9 | 27,7 |
| 86,9 | 27,8 |
| 87,0 | 2,0 |
| 87,0 | 2,0 |
| 87,0 | 7,1 |
| 87,0 | 49,6 |
| 87,0 | 24,7 |
| 87,1 | 18,4 |
| 87,1 | 16,3 |
| 87,1 | 17,1 |
| 87,1 | 30,4 |
| 87,1 | 31,6 |
| 87,2 | 10,7 |
| 87,2 | -3,0 |
| 87,2 | -5,0 |
| 87,2 | 16,2 |
| 87,2 | 12,1 |
| 87,3 | 10,6 |
| 87,3 | 1,8 |
| 87,3 | -3,7 |
| 87,3 | -1,1 |
| 87,3 | 22,6 |
| 87,4 | 13,1 |
| 87,4 | 11,7 |
| 87,4 | 11,9 |
| 87,4 | 19,7 |
| 87,4 | 27,8 |
| 87,5 | 9,7 |
| 87,5 | 0,3 |
| 87,5 | 1,3 |
| 87,5 | -5,0 |
| 87,5 | -4,0 |
| 87,6 | 3,6 |
| 87,6 | 12,9 |
| 87,6 | 26,8 |
| 87,6 | 22,8 |
| 87,6 | 23,5 |
| 87,7 | 8,8 |
| 87,7 | 0,0 |
| 87,7 | 0,0 |
| 87,7 | 15,7 |
| 87,7 | 24,8 |
| 87,8 | 32,6 |
| 87,8 | 9,5 |
| 87,8 | 6,0 |
| 87,8 | 14,6 |
| 87,8 | 24,2 |
| 87,9 | 10,2 |
| 87,9 | 1,0 |
| 87,9 | -2,8 |
| 87,9 | 33,3 |
| 87,9 | 30,0 |
| 88,0 | 20,3 |
| 88,0 | 34,7 |
| 88,0 | 11,8 |
| 88,0 | 7,0 |
| 88,0 | -0,3 |
| 88,1 | 22,2 |
| 88,1 | 7,3 |
| 88,1 | 7,0 |
| 88,1 | 20,1 |
| 88,1 | -1,0 |
| 88,2 | -3,0 |
| 88,2 | 6,1 |
| 88,2 | -2,7 |
| 88,2 | -14,0 |
| 88,2 | -15,0 |
| 88,3 | -8,0 |
| 88,3 | 8,1 |
| 88,3 | 2,5 |
| 88,3 | 0,6 |
| 88,3 | 13,3 |
| 88,4 | -3,0 |
| 88,4 | 0,5 |
| 88,4 | 7,2 |
| 88,4 | 10,4 |
| 88,4 | 9,0 |
| 88,5 | 10,2 |
| 88,5 | 21,4 |
| 88,5 | 2,0 |
| 88,5 | 2,0 |
| 88,5 | 10,4 |
| 88,6 | 13,9 |
| 88,6 | -3,0 |
| 88,6 | -12,0 |
| 88,6 | -6,0 |
| 88,6 | 5,7 |
| 88,7 | 26,5 |
| 88,7 | 10,3 |
| 88,7 | -2,0 |
| 88,7 | -13,0 |
| 88,7 | -13,0 |
| 88,8 | -11,0 |
| 88,8 | -14,0 |
| 88,8 | -10,0 |
| 88,8 | -5,0 |
| 88,8 | 4,0 |
| 88,9 | 0,4 |
| 88,9 | -4,0 |
| 88,9 | -7,0 |
| 88,9 | -13,0 |
| 88,9 | -20,0 |
| 89,0 | -19,0 |
| 89,0 | -13,0 |
| 89,0 | 6,9 |
| 89,0 | 6,1 |
| 89,0 | -3,0 |
| 89,1 | -5,0 |
| 89,1 | 1,0 |
| 89,1 | 12,8 |
| 89,1 | 10,9 |
| 89,1 | -5,0 |
| 89,2 | -9,0 |
| 89,2 | -4,0 |
| 89,2 | 10,9 |
| 89,2 | 3,8 |
| 89,2 | -6,0 |
| 89,3 | -9,0 |
| 89,3 | 19,4 |
| 89,3 | -3,0 |
| 89,3 | -7,0 |
| 89,3 | -9,0 |
| 89,4 | -8,0 |
| 89,4 | 6,7 |
| 89,4 | -8,0 |
| 89,4 | -12,0 |
| 89,4 | -10,0 |
| 89,5 | -4,0 |
| 89,5 | 4,0 |
| 89,5 | -2,0 |
| 89,5 | -10,0 |
| 89,5 | -14,0 |
| 89,6 | -15,0 |
| 89,6 | -17,0 |
| 89,6 | -15,0 |
| 89,6 | -12,0 |
| 89,6 | -16,0 |
| 89,7 | -14,0 |
| 89,7 | -13,0 |
| 89,7 | -5,0 |
| 89,7 | -5,0 |
| 89,7 | 6,8 |
| 89,8 | -6,0 |
| 89,8 | -9,0 |
| 89,8 | -9,0 |
| 89,8 | -5,0 |
| 89,8 | -9,0 |
| 89,9 | -7,0 |
| 89,9 | -7,0 |
| 89,9 | -0,8 |
| 89,9 | -16,2 |
| 89,9 | -23,3 |
| 90,0 | 0,0 |
